# Towards automated inclusion of autoxidation chemistry in models: from precursors to atmospheric implications†

**DOI:** 10.1039/d4ea00054d

**Published:** 2024-07-09

**Authors:** Lukas Pichelstorfer, Pontus Roldin, Matti Rissanen, Noora Hyttinen, Olga Garmash, Carlton Xavier, Putian Zhou, Petri Clusius, Benjamin Foreback, Thomas Golin Almeida, Chenjuan Deng, Metin Baykara, Theo Kurten, Michael Boy

**Affiliations:** a pi-numerics Neumarkt amW. 5202 Austria office@pi-numerics.com; b Chemistry and Physics of Materials, University of Salzburg A-5020 Austria; c Institute for Atmospheric and Earth System Research/Physics, University of Helsinki 00560 Helsinki Finland; d Division of Nuclear Physics, Department of Physics, Lund University P. O. Box 118 221 00 Lund Sweden; e Aerosol Physics Laboratory, Tampere University 33720 Tampere Finland; f Department of Chemistry, University of Helsinki 00014 Helsinki Finland; g Department of Chemistry, Nanoscience Center, University of Jyväskylä FI-40014 Jyväskylä Finland; h Department of Atmospheric Sciences, University of Washington Seattle WA USA; i SMHI/Swedish Meteorological and Hydrological Institute Research Department, Unit of Meteorology/Environment and Climate SE-601 76 Norrköping Sweden; j Atmospheric Modelling Centre Lahti Niemenkatu 73, Lahti University Campus 15140 Lahti Finland; k State Key Joint Laboratory of Environment Simulation and Pollution Control, School of Environment, Tsinghua University 100084 Beijing China; l Climate and Marine Sciences Department, Eurasia Institute of Earth Sciences, Istanbul Technical University Maslak Istanbul 34469 Turkey; m School of Engineering Science, Lappeenranta-Lahti University of Technology 53851 Lappeenranta Finland

## Abstract

In the last few decades, atmospheric formation of secondary organic aerosols (SOA) has gained increasing attention due to their impact on air quality and climate. However, methods to predict their abundance are mainly empirical and may fail under real atmospheric conditions. In this work, a close-to-mechanistic approach allowing SOA quantification is presented, with a focus on a chain-like chemical reaction called “autoxidation”. A novel framework is employed to (a) describe the gas-phase chemistry, (b) predict the products' molecular structures and (c) explore the contribution of autoxidation chemistry on SOA formation under various conditions. As a proof of concept, the method is applied to benzene, an important anthropogenic SOA precursor. Our results suggest autoxidation to explain up to 100% of the benzene-SOA formed under low-NO_*x*_ laboratory conditions. Under atmospheric-like day-time conditions, the calculated benzene-aerosol mass continuously forms, as expected based on prior work. Additionally, a prompt increase, driven by the NO_3_ radical, is predicted by the model at dawn. This increase has not yet been explored experimentally and stresses the potential for atmospheric SOA formation *via* secondary oxidation of benzene by O_3_ and NO_3_.

## Abstract 2

Demonstration of a novel framework producing autoxidation chemistry reaction schemes: an exemplary application for benzene.

## Graphical abstract

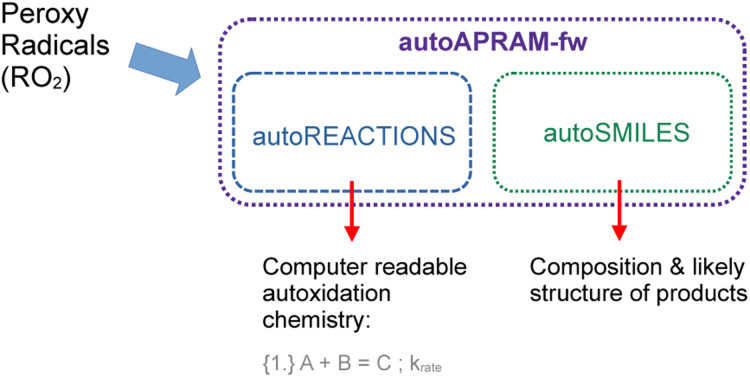

**Environmental significance**

Secondary organic aerosols (SOA) are an important factor in predicting air quality and climate forcing by seeding clouds. During SOA formation, organic oxidation products of sufficiently low volatility partition to the particle phase to form airborne mass. A molecular level mechanistic description of this process seems out of reach for the time being: after the oxidation initiation, the chemistry quickly becomes complex and results in a vast variety of progressively oxygenated reaction intermediates and products, which are impossible to selectively follow by any current experimental methodologies. Similarly, the huge number of potential reaction paths render high-level computational predictions too expensive to derive. We propose an alternative methodology, in which highly sensitive chemical ionisation mass-spectrometry (CIMS) detection is utilized for semi-empirical autoxidation model generation. However, CIMS misses information about the isomeric oxidation pathways. In the first step to overcome this, a collective behaviour of isomeric radical species of unknown structures is considered. This assumption allows us to semi-empirically create lumped chemical schemes.

## 1 Introduction

Adverse effects resulting from poor air quality nowadays represent one of the largest risks to health, causing several millions of premature deaths every year.^1^ This makes air pollution the top environmental mortality risk, posing a much higher risk than *e.g.* polluted water.^2^ In 2019, more than 99% of the global population lived in areas with outdoor PM2.5 (*i.e.* inhalable particles 2.5 μm or less in aerodynamic diameter^3^) levels not meeting WHO guidelines.^4^ In addition, while not being relevant in terms of total mass exposure, ultrafine particles (*i.e.*, PM0.1) pose a severe health risk. After entering the body mainly through the lungs, ultrafine particles can relocate within the body to accumulate in all organs, including the brain.^5^ Typical symptoms include systemic inflammation and can be severe.^6^ The metric to quantify the hazard of inhalable aerosols is, despite being studied intensely, not fully illuminated.^7^ Airborne particle mass, number, and surface, as well as oxidative potential, are considered to be drivers for chronic and acute effects. Recently, Daellenbach *et al.* have suggested secondary organic aerosols (SOA; *i.e.* aerosols formed in the air from gaseous precursors^8^) to play a major role in contributing to aerosol mass burden and, in particular, to its oxidative potential at the European level.^9^ Further, they found that ambient, respirable particulate pollutants' toxicity is dominated by SOA from precursors emitted by anthropogenic activities. While primary aerosol particles impact air quality more locally and formation can be identified comparatively simply, the sources and formation pathways of SOA are complex and transport of precursor molecules can occur over large distances.^10,11^

In the last decade, it has been shown, mainly by means of experiment, that highly oxygenated organic molecules (HOM, as defined by Bianchi *et al.*^12^) can form quickly upon oxidation of precursor volatile organic compounds (VOC) under atmospheric conditions.^12,13^ These, often short-lived, molecules deviate strongly from their parent VOC regarding their physical and chemical properties.^14^ This particularly impacts their SOA forming potential, as the saturation vapor pressure (*p*_sat_) which governs the partition to the particle phase, drops significantly with advancing functionalization of the molecules.^15^ The formation of HOM involves a key chemical process called autoxidation. It describes a chain-like process of intra-molecular H-abstraction and O_2_ addition.^16^ Besides the advances in experimental approaches, findings based on theoretical considerations are increasingly available.^13,17–19^ Yet, there are few studies on deriving a mechanistic concept to predict SOA quantitatively based on the description of autoxidation chemistry under atmospheric conditions and covered by both theory and experiment.^20,21^ The approach by Donahue *et al.*,^20^ due to its formal simplicity, serves well to be applied in large scale models with substantial success. However, as the method does not aim to involve individual species but rather computes average parameters describing the evolution of gas phase chemistry and the partition of condensable vapors to the particle phase, the authors consider it less appropriate to approach a mechanistic understanding of the oxidation process. Thus, in the present work, we follow the approach by Roldin *et al.*,^21^ aiming for a close-to-mechanistic description of the autoxidation chemistry governing the process of molecular rearrangements and oxygen enrichment. We introduce the novel automated alkoxy/peroxy radical autoxidation mechanism framework (“autoAPRAM-fw” – see Methods Section 2.1 The autoAPRAM-fw for details), capable of setting up autoxidation chemistry schemes for any VOC system. Rate coefficients, if available, can be provided as an input. Otherwise they need to be derived from data. In this work, the framework is applied to benzene, a highly abundant and structurally prototypical aromatic molecule, related mainly to anthropogenic activities.^11,22–25^ Lately, there have also been reports of the importance of the biogenic emission of benzene and other BTEX species (*i.e.*, toluene, ethylbenzene and xylenes).^26,27^ In this work, we constrain the rate coefficients of reactions related to benzene autoxidation chemistry. The approach is tested by reproducing pure gas-phase experiments and experiments investigating the SOA formation potential under high and low NO_*x*_ conditions. Further, atmospheric implications are predicted by carrying out a parameterized study computing SOA mass yields for a range of benzene and NO_*x*_ levels. Finally, atmospheric trajectory simulations are conducted to investigate the potential contribution of autoxidation chemistry and its impact on the benzene SOA evolution in the atmosphere.

## 2 Methods

In a first step, chemical reaction types describing benzene autoxidation chemistry are gathered from the literature (Section 2.2). Alongside^28,29^ a method describing the basic degradation of VOC in the atmosphere, we apply the autoAPRAM-fw to add, to data missing, the depiction of benzene autoxidation chemistry (Section 2.1). Simulation of pure gas-phase experiments allows missing information on reaction rate coefficients to be constrained (Section 2.3). In the next step, we assign potential molecular structures to the gas phase species formed (Section 2.4). This is done in order to compute their saturation vapor pressures by exploring several approaches such as state-of-the-art quantum chemistry calculations or group contribution methods (Section 2.5). In order to test the potential of the approach in predicting SOA formation, we replicate chamber experiments of benzene–OH oxidation in the presence of seed aerosol applying a detailed micro-physics and chemistry box model (Section 2.6). Potential atmospheric implications are illustrated by studying SOA yield under idealized atmospheric conditions (Section 2.6.4). This contrasts with findings from close-to-realistic Lagrangian-type atmospheric transport model calculations (Section 2.6.3).

### 2.1 The autoAPRAM-fw

**Fig. 1:**
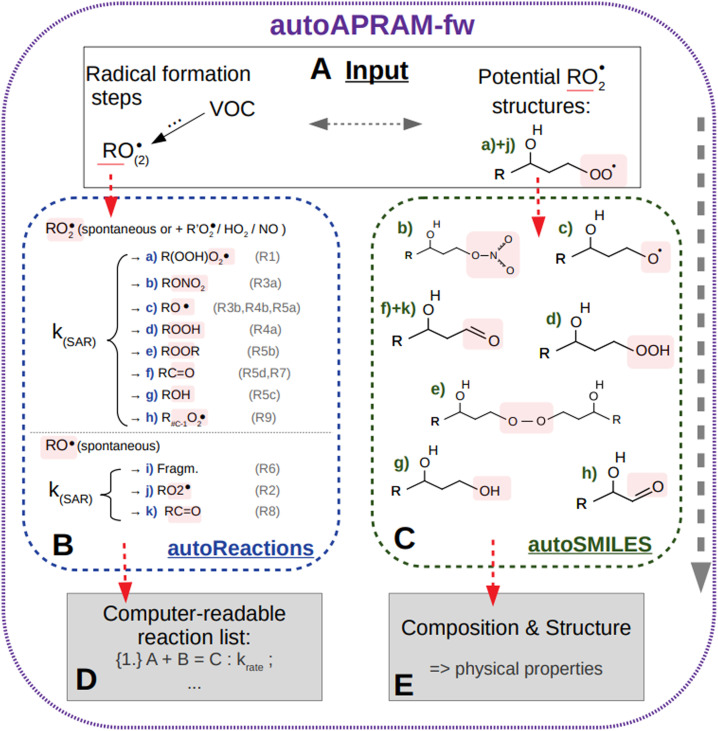
Overview of the autoAPRAM-fw. Sector (A) represents the input to submodules autoReactions (B) and autoSMILES (C), respectively. Outputs comprise a Fortran readable chemistry module from autoReactions (D), and molecular information on the species formed in autoSMILES (E). Note that the submodules may be run individually.

The automated alkoxy/peroxy radical autoxidation mechanism framework (autoAPRAM-fw) serves to generate a model describing autoxidation chemistry of VOCs in the gas phase. The chemistry scheme is based on MCM v3.3.1 describing the degradation of VOCs in the atmosphere.^28,29^ The autoAPRAM-fw consists of two modules: (a) autoReactions, which is applied to generate the differential equations describing autoxidation chemistry. It also defines product species names and creates a computer readable chemistry model. Further, it can be used to describe functional groups of product molecules; (b) autoSMILES, which creates potential structures of reaction products described by the SMILES convention.^30^ A graphical description of the framework is shown in Fig. 1.

As an input autoAPRAM-fw takes peroxy radical (RO_2_) names formed in MCM together with their atomic composition. Further, the RO_2_ composition of species formed by autoxidation chemistry which are not described in MCM needs to be provided by the user. They are not created by autoSMILES. After specification of the reaction types considered, autoReactions sets up a chemistry module. Reaction rate constants can be based on theory if available (*e.g.* structure–activity relationships – SAR) or can be set manually. Physical properties of the species formed can be determined from their probable structures, computed by autoSMILES, for the reaction types specified. In the present work we investigated the physical properties of product species using vapor pressure prediction methods “EVAPORATION”,^31^ “NANNOOLAL”^32^ and “MYRDAL/YALKOWSKI”^33^ for all species, and the selected species have been investigated using a high performance quantum chemistry method (COSMO-RS^34–36^).

The two modules autoReactions and autoSMILES can be run individually. The first one can be applied repeatedly to constrain the gas phase chemistry. Note that although the model can be used to produce close to explicit gas phase chemistry based on theory, there is still need for tuning the reaction rate constants such as branching ratios. AutoSMILES may be rerun to investigate the effect of varying structures of input-RO_2_ isomers. The framework itself is written in a way to describe any type of VOC undergoing autoxidation.

We are currently exploring the possibility of applying machine learning in the following way: a chemical scheme created by the autoAPRAM-fw is used together with MCM chemistry to represent a VOC system. Next, we optimise the newly developed chemistry schemes by performing an in-depth Bayesian analysis of the VOC system. To sample all model parameters, we use methods like Hamiltonian Monte Carlo with automatic differentiation and Markov Chain Monte Carlo (MCMC).^37,38^ Both techniques provide efficient solutions to high-dimensional problems. The goal is to sample and quantify model parameters to increase accuracy and reliability in chemical schemes.

### 2.2 Setting up the gas phase chemistry

The gas phase chemistry scheme generated is based on the master chemical mechanism (MCM^v3.3.1^), describing tropospheric degradation of hydrocarbons. However, it does not yet include autoxidation chemistry which is added by deploying the reaction types below:R1*
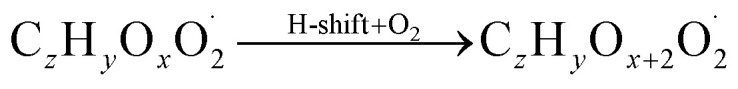
R2*
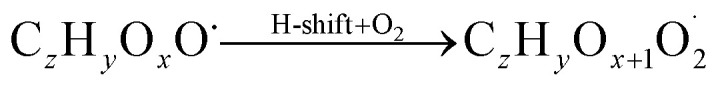
R3a
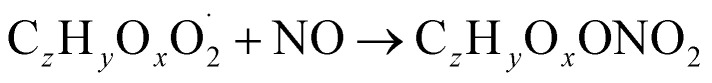
R3b
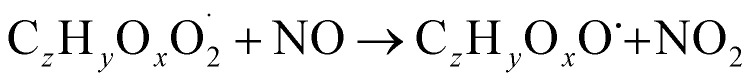
R3c
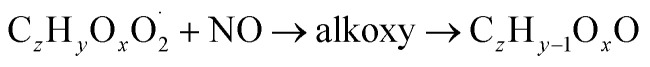
R4a
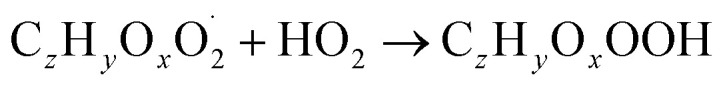
R4b
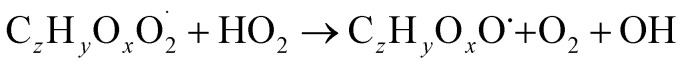
R5a
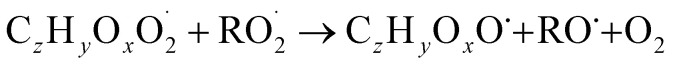
R5b
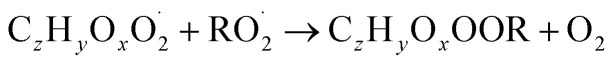
R5c
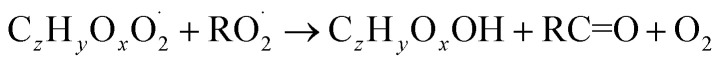
R5d
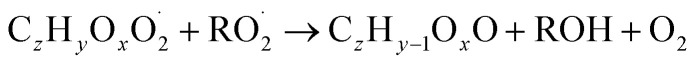

R6 C_*z*_H_*y*_O_*x*_O˙ → fragmentation

R7*
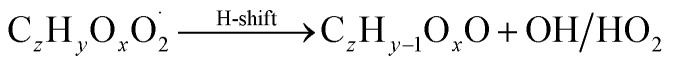
R8*
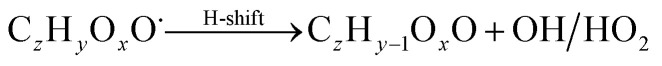
R9*
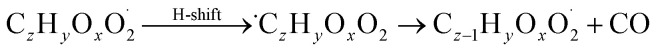


The considered reaction types are described in Bianchi *et al.*^12^ (reactions (R1*) and (R2*)), Orlando & Tyndall^39^ (reactions (R3)–(R8*)) and Crounse *et al.*^40^ (reaction (R9*)). In the current version, reactions marked with a “*” require constraining of the overall rate constants, while others require the distribution between product channels and consider a default overall rate (derived from MCM).

Upon choice of the reaction types considered, a computer readable chemistry model is created automatically by the autoAPRAM-fw. This allows new chemical systems to be quickly set up, various atmospherically relevant SARs to be considered or different RO_2_ isomers to be tested in a convenient way. The autoxidation chemistry connects to the MCM chemistry scheme mainly by using peroxy radicals formed in MCM.

### 2.3 Reaction rate coefficients

Constraining the reaction rate coefficients, and branching ratios in case of several product pathways, is neither trivial nor fully deterministic. Accordingly, potential solutions are to be expected from this method rather than the only “right answer”. In the present work, the following method has proven successful. It is based on the comparison of simulated mass spectra and experimentally determined high resolution mass spectra from nitrate (NO_3_^−^) chemical ionization mass spectrometry (CIMS) measurements. Note that these spectra need to be interpreted with care as discussed in the ESI section “Interpreting ion count data from CIMS measurements”.† Ideally, the comparison is done for an evolving chemistry. Experimental data should be taken from well-defined experimental setups (*i.e.*, negligible wall effects; well-defined precursor consumption; well-known chemical systems). In the present work, we deployed flow tube experimental data, where available, for this task. The individual steps described below refer to a single specified point in time of simulation and experiment for which the chemistry code is constrained.

Step 1 is for adjusting the hydrogen shift rates in order to reproduce observed peroxy radical levels (reaction (R1*)). This procedure has to be repeated after each of the other steps as H-shift rates interfere with the RO_2_ concentrations. Peroxy radicals with low oxygen number are not detected at full efficiency in NO_3_^−^-CIMS.^41^ Thus, their simulated concentration may well exceed the measured value.

In step 2, the rate coefficients determining dimer formation are constrained as the dimers are likely to be detected with high efficiency. Note that the least oxidised dimers may, similar to the monomers, be detected with reduced efficiency. Further, the branching ratios for RC=O (R5d) and ROH (R5c) formation are assigned. The formation of an alkoxy radical is calculated based on the difference between the overall rate coefficient describing RO_2_ + RO_2_ and the individual closed shell forming rate coefficients determined before (reactions (R5b)–(R5d)). Computing these numbers can be done best in case the RO_2_ + RO_2_ reactions are dominating compared to RO_2_ + HO_2_ or RO_2_ + NO.

Step 3 comprises the determination of the branching ratio RO_2_ + HO_2_ → (a) ROOH or (b) RO + OH + O_2_ described in reactions (R4a) and (R4b). The overall rate coefficient for RO_2_ and HO_2_ is obtained from MCM chemistry for the given peroxy radical. Experimental conditions featuring a dominating HO_2_ sink for RO_2_ are favorable for taking this step. Otherwise, it is not possible to distinguish between species formed from RO_2_ + RO_2_ and RO_2_ + HO_2_ or RO_2_ + NO.

Step 4 requires the determination of isomeric closed shell species formation by intramolecular hydrogen abstraction from α-hydro(pero)xyl functional groups by O_2_. As a result a carbonyl functional group is formed (reaction (R7*)). This can be an important pathway under clean conditions, when RO_2_, HO_2_ and NO are not the dominating sinks of RO_2_.

Step 5 is the determination of RONO_2_ formation under high NO_*x*_ conditions (reactions (R3a) and (R3b)). Here, the NO_*x*_ level (relative to HO_2_ and RO_2_) is not too important as the nitrated products are distinctive (*i.e.*, RONO_2_ cannot be confused with ROOH or ROH). This step also includes formation of alkoxy radicals that can either fragment (reaction (R6)), undergo RO-autoxidation to form RO_2_ species (reaction (R2*)) or form closed shell species (reaction (R8*)). Under high NO conditions, the formation of RO_2_*via* RO seems to be an important path to reproduce the observed peroxy radical levels.

Elimination of CO after hydrogen abstraction from an aldehyde group is considered in step 6 (reaction (R9*)). Resulting peroxy radicals have one less carbon atom in the chain compared to their parent VOC. For those species, steps 2–5 have to be repeated.

In the present work, steps 1 to 6 (except for steps 4 and 5) have been carried out for data from a flow tube setup. Step 5 was done for data gathered from a steady state type chamber experiment applying high NO_*x*_ levels. Due to lack of experimental data from chamber experiments mimicking clean conditions rate coefficients to be constrained in step 4 were estimated (*i.e.* low VOC reacted, low NO_*x*_). Further, higher generation RO_2_ species are considered. Note that rate coefficients constrained from CIMS data to a great extent coincide with predictions from SARs for RO_2_ + RO_2_/HO_2_/NO:^18,42^

(1) ROOR: based on observed benzene ROOR formation and RO_2_ concentrations,^43^ we found the following relationship between the molecular mass of the RO_2_ species and the rate coefficient:1
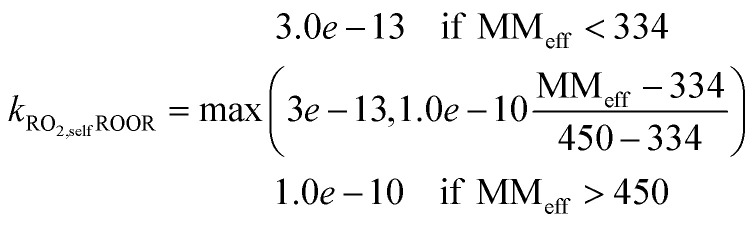
where MM_eff_ is the effective molecular mass of the reacting species (*e.g.*, the summed mass of both species) which is similar to the molecular mass for C6 RO_2_ species. For C5 RO_2_, it is the molar mass plus the mass of a carbon atom for C5 peroxy radicals. Rate coefficients for ROOR formation for the self-reaction of key RO_2_ species applied are plotted against *n*CON (*i.e.* the number of C, O, and N atoms in the RO_2_ structure – see Jenkin *et al.*^18^ for more information) in ESI Fig. S10.† While the rate coefficients for RO_2_ self reaction from SAR and the dimer formation rates in autoAPRAM show a similar trend (increasing rate with increasing *n*CON and leveling off at the maximum value), the upper limit in the present work is higher by roughly a factor of 10. However, ROOR formation rates well above 10^−10^ cm^3^ s^−1^ were reported recently (*e.g.*, Berndt *et al.*^44^ or Molteni *et al.*^45^). Further, the rates from autoAPRAM show a stronger dependence on *n*CON. A potential reason might be a changing and unknown degree of RO_2_ (primary, secondary or tertiary) and substitution, respectively.

(2) ROOH yield: while the overall rate coefficient for RO_2_ + HO_2_ species is adopted from MCM, the branching ratios (see reactions (R4a) and (R4b)) were constrained against experimental data. To reproduce the experimental findings (by means of modelling), ROOH yields between 0.5% and 60%, with an increasing yield-trend towards higher oxidized species, are applied. For comparison, the bicyclic peroxy radical (named BZBIPERO2 in the present work), features an estimated ROOH yield of roughly 1%.^42^

(3) RONO_2_ yield: similar to reaction (R4), for the reaction RO_2_ + NO (reaction (R3)), overall rate coefficients are obtained from MCM generic rate coefficients and branching ratios towards RONO_2_ and RO are constrained against experimental data. Again, the alkoxy branch is dominating. RONO_2_ yields range from 0.2% to 4% which is in line with rate coefficients reported for similar molecular structures (0.3–2%).^18,42^

### 2.4 The molecular structures

The autoSMILES sub-module of the autoAPRAM-fw relies on SMILES-format inputs of potential peroxy radical structures in order to compute closed shell monomer and dimer structures. However, this information is currently not available for the vast majority of the considered RO_2_ species. As a consequence, an approach mainly based on theory is applied. For a few decades already a specific structure, the bicyclic peroxy radical (see ESI Fig. S11†) has been suggested to be a key RO_2_ structure that forms upon a single OH attack on benzene followed by O_2_ addition.^23,25,42,46^ This is in line with CIMS data showing C_6_H_7_O_5_ as the peroxy radical with the smallest oxygen number (note: an odd oxygen number and containing six carbon atoms; *e.g.* Molteni *et al.*^43^ or Wang *et al.*^25^). Accordingly, we base our considerations on this structure. To predict potential structures of other RO_2_ species, we considered unimolecular rearrangements, bimolecular reactions and multiple OH attacks. The aim is to provide at least one potential structure and its formation *via* plausible reaction paths for each peroxy radical composition detected by the mass spectrometer. The resulting formation scheme is shown in the ESI (Fig. S12†). Note that the focus of this scheme is to predict potential peroxy radical structures rather than suggesting an explicit reaction scheme. Further, radical termination pathways have been ignored in this illustration as the focus is put on radical retaining reactions. The resulting list of peroxy radical structures most likely represents only a fraction of RO_2_ species involved in the autoxidation chemistry.^47^

We assume that the absence of information on RO_2_ structures, from either experiment or theoretical considerations, will most likely not change in the near future. However, due to the fact that theoretical considerations are applicable for suggesting structures at least for early-stage reaction products and beyond, while new technologies in mass spectrometry, such as versatile, structure specific charging techniques, are emerging,^41,48^ we expect that this gap in knowledge will continuously decrease.

### 2.5 Deriving the saturation vapor pressure

The saturation vapor pressure is an important quantity to derive the partition of a species between the vapor and condensed phase.^49^ Ideally, it is determined experimentally. However, as the present work involves hundreds of different species, this approach is not feasible. The vapor phase molecules formed upon oxidation of precursor VOC are characterized by their potential structures described by the SMILES convention. Corresponding *p*_sat_ are computed by means of two different approaches: for all species, *p*_sat_ is derived by applying group contribution methods, namely EVAPORATION,^31^ NANNOOLAL^32^ and MYRDAL/YALKOWSKI.^33^ ESI Fig. S13† graphically provides an overview of the volatility distribution. More information on the methods and how they compare to high-level quantum chemical calculations is provided in the ESI text.†

All SOA simulations in this work are employing the group contribution methods to describe the species' vapor pressures. Variation of the SOA is spanned by methods NANNOOLAL and MYRDAL/YALKOWSKI, while EVAPORATION forms the center-line of the results. In case a single SOA result is reported (*i.e.* for atmospheric simulations), the *p*_sat_ is based on EVAPORATION.^31^ However, note that there is no evidence on which method computes best the *p*_sat_ of the unknown variety of structures.

### 2.6 Numerical simulations: ADCHAM & ADCHEM applications

The aerosol dynamics gas and particle phase chemistry model for laboratory CHAMber studies (ADCHAM^50^) and the trajectory model for aerosol dynamics, gas and particle phase CHEMistry and radiative transfer (ADCHEM^21,51^) are deployed in the present work. ADCHAM serves to compute phase change of inorganics (H_2_SO_4_, NH_3_, HNO_3_) and organics (MCM and autoAPRAM species) by considering Brownian coagulation, condensation, evaporation and dissolution. To ease computation, only organic species with a *p*_sat_ lower than 1 Pa (which corresponds to a saturation mass concentration C* or roughly 0.1 g m^−3^) are considered to potentially partition to the particle phase. Diffusion in the carrier gas is described by Fuller's method. Detailed model inputs can be found in the ESI (section “Model input specifications”).†

#### 2.6.1 Flow tube

**Fig. 2:**
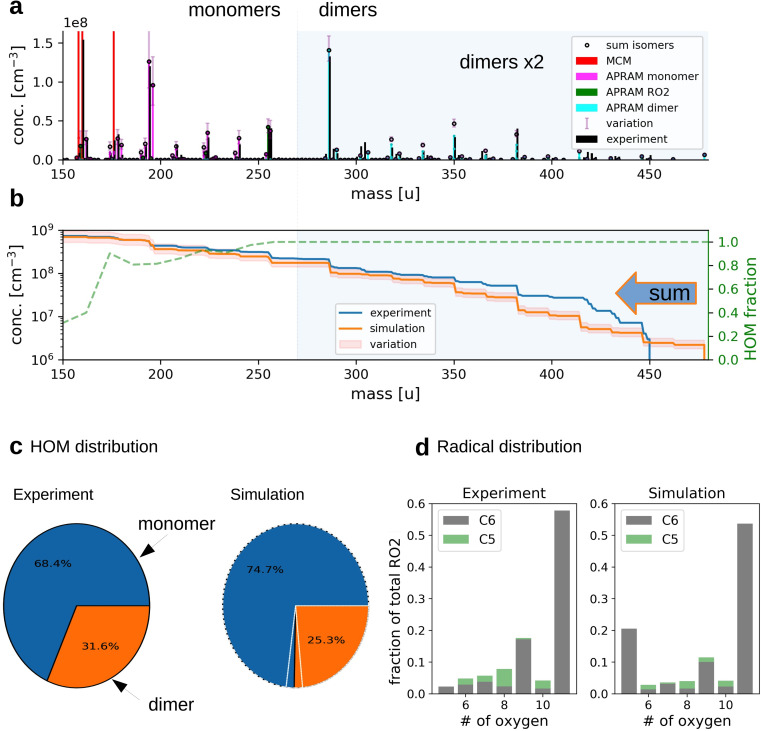
The simulation of OH oxidation of benzene in the presence of UV light. Panel (a) depicts the gas-phase molecular mass distribution from flow tube experiment^43^ (black bars) and the simulation results (colored bars show different subsets of species from one simulation; “sum isomers” represents the sum of simulated isomers; note that dimer concentrations are raised by a factor of 2 to increase readability). Panel (b) shows cumulative representation of the model results, starting at the upper observed molecular mass limit. The secondary axis indicates the computed, unitless HOM fraction. Panel (c) depicts the distribution of HOM species between the monomers and dimers (variation due to wall loss assumptions is indicated by white lines for simulated data). Panel (d) illustrates the frequency of peroxy radicals of different oxygenation states and carbon number. The insets indicate radicals with 6 (“C6”) and 5 (“C5”) carbon atoms.

In the flow tube runs, potential loss of condensable vapors to the walls is considered to lie between (a) zero influence and (b) cross section averaged deposition to the walls for a fully developed laminar flow as described by Ingham.^52^ These scenarios form the variation in species concentration as shown in Fig. 2 panels (a)–(c), while the center line is calculated assuming reduced loss by 50% compared to applying formulations suggested by Ingham. This is done as the flow tube comprises a laminar flow field and because reactive species are added at the center line of the flow tube to avoid the influence of the tube walls.

#### 2.6.2 Chamber runs

**Fig. 3:**
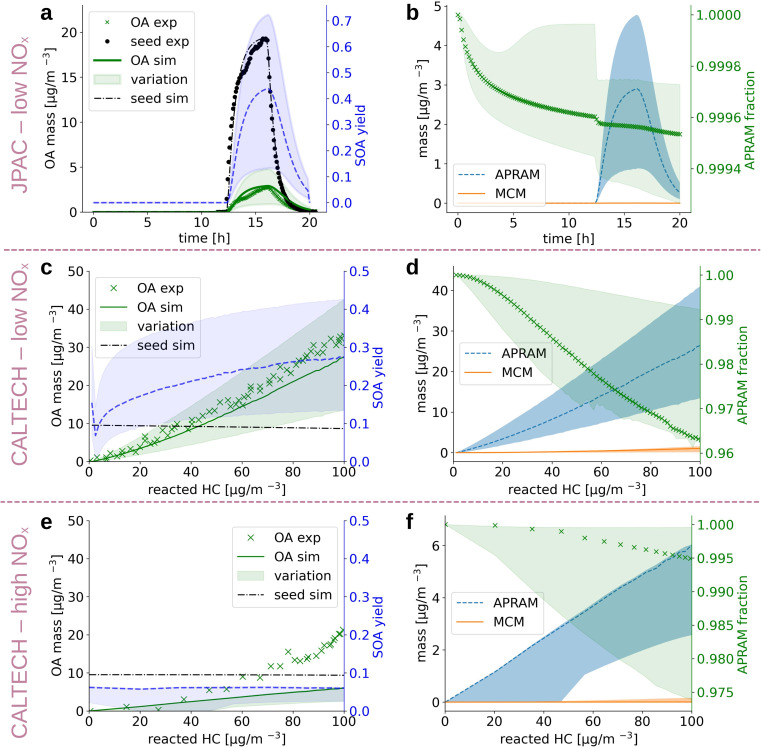
The simulation results when reproducing experimental chamber runs (panels (a and b) and (c–f) refer to the JPAC chamber and CALTECH chamber, respectively). Panels on the left hand side (a, c and e) depict the organic aerosol formed (simulation: “OA sim”; experiment: “OA exp”) as well as the seed aerosol present (simulation: “seed sim”; experiment: “seed exp”), respectively, shown on the left hand ordinate. The unitless, simulated SOA yield (dashed line; shaded area denotes the variation) is illustrated by the right hand ordinate. Results are shown as a function of reaction time (panels (a and b)) and reacted hydrocarbon (panels (c–f)). Panels (b, d, and f) contain computed information on the distribution of mass formed by MCM chemistry and by the autoxidation chemistry (APRAM), respectively. The variation of modeled data results from the different *p*_sat_-determination methods applied.

ADCHAM is set up to reproduce experiments in the JPAC^53^ and the Caltech^54^ chamber, respectively. In JPAC, gas phase oxidation of benzene in the presence of NO_*x*_ is simulated (ESI Fig. S15†) as well as under low NO_*x*_ conditions with additional seed aerosol (Fig. 3, panel (a)). Simulations in the Caltech chamber are made for high and low NO_*x*_ conditions in the presence of the seed aerosol. Note that the JPAC chamber features a constant flow (into and out of the chamber) of precursors that are oxidised and removed while in the Caltech chamber, the precursor concentration is highest at the start and is depleted throughout the experiment. Partition of condensable molecules between the gas phase and the chamber walls is described by considering the loss of gas phase species i to the chamber wall, based on first order wall loss rates, and the evaporation of volatiles (*i.e.* species with *p*_sat_ higher than 10^−7^ Pa are considered in this work) from the reservoir back to the gas phase. A detailed description of the partition formulations can be found in Roldin *et al.* (2019).^21^ Note that for both, the Caltech and JPAC chambers, the first order loss rates are based on experimental data. For Caltech, it is determined by Zhang *et al.*^55^ to be roughly 10^−4^ s^−1^ for the benzene system; in JPAC, the first order loss rates are determined for C_10_H_16_O_8_ (1/75 s^−1^; see Ehn *et al.*^14^) and extrapolated to the species i based on the ratio of diffusion coefficient *D*_i_/*D*_C10H_16_O_8__.

#### 2.6.3 ADCHEM & atmospheric simulations

We implemented the APRAM benzene mechanism in the Lagrangian chemistry transport model ADCHEM^21,51^ and simulated the atmospheric chemistry, aerosol dynamics and secondary organic aerosol formation downwind the Copenhagen/Malmö urban region. For a detailed description of the implemented ADCHEM version the reader is referred to Roldin *et al.*^21^ and references therein. Specifically for the present work, ADCHEM was run along pre-calculated HYSPLIT^56^ air mass trajectories that started 7 days upwind Malmö and then continued 3 days downwind. Analogous to Xavier *et al.*^57^ the anthropogenic, biogenic and oceanic emissions of trace gases and primary particles were considered using CAMs global emission inventories^58^ and the sea spray aerosol parameterization by Sofiev *et al.*^59^ The chemical mechanism, which is based on the master chemical mechanism,^60–62^ also includes the monoterpene PRAM mechanism,^21^ a novel dimethyl sulfide (DMS) multiphase chemistry mechanism,^63^ and the APRAM benzene reaction scheme from the present work. We selected three air mass trajectories which arrived in Malmö before noon on April 28, 2021 (“9 am”, “10 am” or “11 am” local wintertime (UTC + 1 hour)), continued over Copenhagen, passed over the Northern Danish agricultural region in the afternoon/night and then spent >2 days over the North Sea before the air mass was transported inland over the harbor and urban regions of Antwerp and Brussels. The selected cases demonstrate how the benzene radical chemistry and SOA formation changes upon several consecutive daytime and night-time cycles with surface layer NO_*x*_ levels ranging from ∼30 ppbv over Copenhagen to ∼70 ppt over the North Sea.

In this work, the third case, “11 am”, is discussed in detail. Plots showing respective results for the cases “9 am” and “10 am” can be found in the ESI.† Data are discussed from 1 day upwind to 3 days downwind Malmö, except for the airmass trajectories (full trajectories over the 10 day simulation period are given in ESI Fig. S1†). The simulation days 7 to 1 day upwind are applied to allow a build-up of the gas-phase species.

#### 2.6.4 Parametric yield study

Aiming to provide an overview of possible benzene mass yield values, we conducted a series of numerical simulations under atmospheric-like, daytime conditions. Mass yield, in this context, refers to the maximum observed mass yield under predefined conditions as is typically reported in the literature (*e.g.*, Ng *et al.*^64^). Briefly, OH attacks benzene, leading to the formation of peroxy radicals, which can undergo autoxidation to form condensable materials that accumulate on the seed aerosol. The presence of other VOC species, represented by methane and NO, interferes with the autoxidation process as recently shown by McFiggans *et al.*^65^

Detailed information on the approach can be found in the ESI section “Description of parametric yield calculations”.† ESI Table ST2† provides an overview of the inputs.

## 3 Results

### 3.1 Pure gas-phase simulations

The applied chemistry scheme is set up by coupling the basic description of tropospheric benzene degradation, obtained from the master chemical mechanism (MCM; see the Methods section for details), to the autoxidation chemistry scheme created by the autoAPRAM-fw. Missing information on reaction rate coefficients is constrained based on flow tube and chamber experiments. Abridged, MCM describes the degradation of VOC species including the formation of peroxy radicals which produce oxygenated closed shell species by unimolecular or bimolecular reactions. Multiple generations of oxidations are considered. Thermodynamically more stable fragmentation products build up *via* the alkoxy radical paths, ultimately forming CO and CO_2_.^28,29^ Formally, the autoAPRAM-fw adds the formation of high oxygen containing species by considering autoxidation chemistry in addition to the MCM chemistry scheme. Besides various closed shell product forming reactions, autoxidation (consisting of an intramolecular hydrogen abstraction followed by the addition of O_2_) of peroxy-(RO_2_) and alkoxy-(RO) radicals is the key process. Implementing the combined chemistry scheme into the aerosol dynamics model ADCHAM^50^ enables computational reproduction of pure gas-phase experiments focusing on autoxidation and competing reactions.

Computer simulations of a flow tube setup employed by Molteni *et al.*^43^ show the applicability of the approach (Fig. 2, panels (a)–(d); dimer concentrations were raised by a factor of 2 for better visibility). Note that monomers refer to reaction products having the same or smaller carbon number compared to the VOC precursor. Dimers form by the accretion reaction of monomer-radicals. Variation of the results originates from diffusive losses and is described in detail in the Methods section (see Section 2.6). As depicted by panel (a), the model is able to reproduce the atomic mass distribution measured using a nitrate chemical ionization mass spectrometer (NO_3_^−^-CIMS). Further, the monomer to dimer ratio and the radical distribution are reproduced (Fig. 2, panels (c) and (d)). Deviation of modeled and experimental concentrations is observed for the least oxygenated peroxy radicals and their closed shell derivatives (*i.e.*, species with molecular mass below 180 amu; without reagent ions), excluding dimers (panel (a)). This, however, is not surprising as the detection efficiency of these species is lowered due to their structural specifications.^41^

Slight underestimation of the formation of dimers in the range above 400 amu is observed (see Fig. 2b). However, no sound explanation is available yet as the precursor molecules, highly oxidized RO_2_, are assumed to be detected with the highest efficiency and the formation of their dimeric products is considered to proceed close to the kinetic limit (∼10^−10^ cm^3^ s^−1^). Potentially, the underestimation of simulated dimer formation featuring high atomic mass might originate from ignored oxidation of closed shell dimers (of lower oxygen content), followed by autoxidation steps or by general underrepresentation of RO_2_ concentration.

Since no flow tube data is available for benzene under elevated NO_*x*_ ([NO_*x*_] > 1 ppbv) conditions, we used results from a steady state chamber setup to constrain the model.^66^ Note that we consider the retrieval of potential rate coefficients from the flow tube setup to be more accurate. The reason is the degree of complexity in mixing reactors featuring comparatively long residence times (processes to consider: chemical reactions, mixing, wall partitioning, nucleation, aerosol formation, in- and out-flux). In comparison, the flow tube represents a “simple” setup (processes to consider: chemical reactions and limited wall interaction). Accordingly, we consider the chamber-derived rate coefficients to be less accurate. The only rate coefficients derived from the elevated-NO_*x*_ experiments are reaction types (R3) and (R8*) (see Section 2.2 “Setting up the gas-phase chemistry” for more information on the reaction types).

While the monomer distribution is largely reproduced with regard to nitrate species formation, the dimer concentrations are underestimated by the model due to the scavenging of their precursor species, the peroxy radicals, by NO (see ESI Fig. S15†). The approach of stable closed shell species (*i.e.*, closed shell products are not further oxidised) formation in the autoAPRAM-fw produces, compared to experiment, dimers of similar mass and composition. However, their abundance is considerably underestimated (∼90%) under high NO_*x*_ conditions. Note that, in contrast to low NO_*x*_ conditions, the simulated HOM dimer fraction is much lower (dimer fraction ∼ 2%). Detailed results under high NO_*x*_ conditions are discussed in the ESI (section “High NO_*x*_ conditions”).†

### 3.2 Partition to the particle phase

Alkoxy and peroxy radicals form upon oxidation of the parent VOC. The radicals can undergo autoxidation and, as a consequence, isomerize to multi-functionalized molecules. The properties of these species deviate substantially from the parent VOCs. In order to compute their properties, we derived the potential molecular structures. Due to the fact that these molecules are often short lived, diverse (with regard to chemical composition and, most likely, isomeric variation), and only comprise a small fraction of the pool of reaction products, their structures have not yet been approached experimentally, besides a few exceptions.^67^ Thus, we base the structural suggestions on theoretical work and available knowledge on similar reaction classes (see Sections 2.2 and 2.4). The autoSMILES sub-module of the autoAPRAM-fw computes likely structures for all closed shell species. Potential structures of the peroxy radicals serve as an input. The applied approach is discussed in detail in the Methods section. Partitioning of oxidation products between the gaseous and the condensed phases, in the presence of a condensation sink, is strongly affected by the species saturation vapor pressure. The *p*_sat_ is obtained by applying group contribution methods (*e.g.*, O'Meara *et al.*^68^) to all structures. A quantum chemistry based statistical thermodynamics method, conductor-like screening model for real solvents (COSMO-RS^34–36^), is applied exclusively to a few species as it requires detailed analysis and extensive computational resources (see Section 2.5 Deriving the saturation vapor pressure).

Despite being an intensely studied molecule only a few benzene oxidation experiments conducted under well defined, atmospherically relevant conditions which report SOA information can be found in the literature.^64,66^ In the present work we set up the ADCHAM model for the CALTECH^54^ and JPAC^53^ chambers to simulate seeded OH oxidation in the presence of UV light. Fig. 3 depicts simulation results and experimental data. Panels (a), (c) and (e) compare reported^64,66^ and simulated aerosol mass formed and show calculated SOA mass yield based on the simulated data. Note that SOA mass yield is defined as SOA formed divided by benzene mass reacted. Values for aerosol mass formed agree well with the observations for panels (a) and (c), representing low NO_*x*_ conditions ([NO] and [NO_2_] < 1 ppt). Accordingly, the mass yields reported in the literature were reproduced under reported conditions. Garmash *et al.* derived a maximum SOA mass yield of 40%, while we found 42% (12% to 71%), and Ng *et al.* observed a maximum mass yield of 37% which compares to 28% (14% to 42%) in the simulation in the CALTECH chamber. For the high NO_*x*_ case shown in panel (e), the SOA formation potential is underestimated (simulated mass yield of roughly 7% (3% to 7%) *vs.* experimentally observed 26%). Ranges in simulated SOA yield result from different *p*_sat_-estimation methods applied (see Section 2.5 Deriving the saturation vapor pressure). Fig. 3, panels (b), (d) and (f) represent the contribution of MCM and autoAPRAM species, respectively, to the organic aerosol formed. In all three cases, the model suggests clear dominance (>94%) of the species formed *via* autoxidation. In the steady state experiment from the JPAC chamber, the fraction of autoAPRAM species is highest (>99%). Notably, the heterogeneous nucleation at the seed particle surface is initiated by species formed by autoxidation in all simulations (*i.e.*, the autoAPRAM fraction approaches 100% at *t* → 0 s after seed aerosol addition which corresponds to low reacted hydrocarbon in the CALTECH case). Further, note a conceptional difference, “steady state” *vs.* “evolving chemistry” between experimental setups related to Fig. 3 panels (a) and (b) (*i.e.*, resembling the JPAC chamber) and (c)–(f) (*i.e.*, resembling the CALTECH chamber), respectively, which is highlighted by the evolution of the seed aerosol: while panel (a) shows an increase in seed aerosol upon production, followed by its decay, panels (c) and (e) represent an almost constant seed aerosol concentration present in the chamber.

### 3.3 Atmospheric implications

VOC concentrations have been reported almost all around the globe. Benzene is a prototypical aromatic molecule whose emission is related to mainly anthropogenic activities and is detected at high levels in many environments.^26,27,69^ Besides benzene being toxic itself, its basic risk to health is *via* the oxidation in the atmosphere to contribute to the formation of nitrogen-containing species and secondary organic aerosols.^70^ If emitted by a combustion process, benzene is mixed with NO_*x*_. While NO_*x*_, and in particular NO, concentrations decrease relatively quickly upon transport away from its source, benzene has an atmospheric lifetime of roughly 12 days.^71^ As a consequence, benzene is oxidized in the presence of varying NO_*x*_ mixing ratios. To demonstrate this process, we selected airmass trajectories (a) originating from the clean air above the Arctic Ocean between Greenland and Svalbard; (b) traveling over an urban European region (Malmö/Copenhagen); and (c) are characterized by changing NO_*x*_ conditions during benzene-SOA formation.

**Fig. 4:**
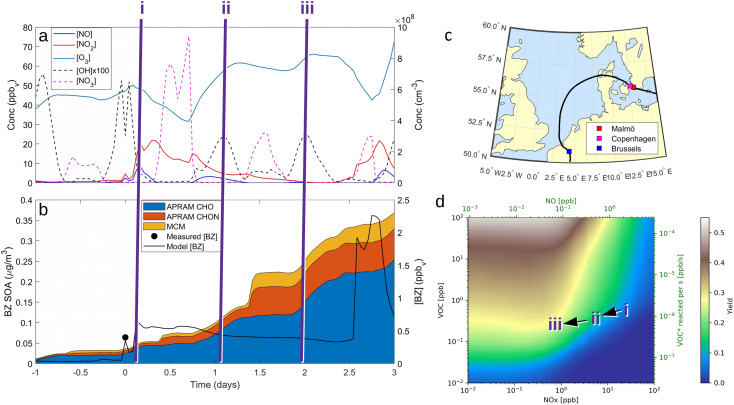
ADCHEM results along the case 3 (“11 am”) air mass trajectory. Panel (a) shows the modelled gas-phase concentrations of NO, NO_2_, O_3_, OH and 
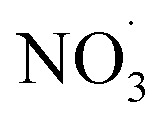
 from 1 day upwind (−1 day) to 3 days downwind Malmö. Note that OH concentration is scaled up by a factor of 100 to increase readability. Panel (b) shows the modelled benzene gas-phase concentrations (model [BZ]) and the modelled benzene SOA mass concentrations of non-nitrate APRAM species (APRAM CHO – species of atomic composition C_*x*_H_*y*_O_*z*_), APRAM organonitrates (APRAM CHON – nitrogen-containing species) and MCM species. The modelled benzene is also compared with the observed benzene concentrations at the measurement station Dalaplan in Malmö (measured [BZ]). The air mass trajectory path is displayed in panel (c). Panel (d) illustrates the computed SOA mass yield (unitless) from OH oxidation of benzene in the presence of different levels of NO_*x*_ and VOC. Bottom and top abscissas depict NO_*x*_ and NO concentrations. VOC mixing ratio and VOC turnover are shown by the left and the right hand ordinate, respectively. The color code indicates the mass yield value. Note the highlighted points in chemical space (“i”, “ii” & “iii”) in all panels.

To showcase the effects of autoxidation chemistry on benzene atmospheric oxidation and SOA formation, the Lagrangian atmospheric chemistry transport and aerosol dynamics model ADCHEM was employed.^51^ A more detailed description of the model setup is provided in the Methods section (2.6 Numerical simulations: ADCHAM & ADCHEM applications) and references therein. It was configured to simulate three air-mass trajectories arriving at Malmö, southern Sweden in the end of April, 2021. The simulations are named by the time the air-masses pass the city of Malmö: “9 am”, “10 am” and “11am” (UTC + 1). For the selected cases, air masses from the Arctic Ocean between Greenland and Svalbard are transported over Scandinavia and the Baltic Sea before they arrive in Malmö 7 days later. Downwind Malmö the air masses are transported over Copenhagen and spend ∼48 hours over the North Sea before they arrive near Brussels (see ESI Fig. S1†). Results are analyzed in detail from one day upwind Malmö to the end of the simulation (Fig. 4). Based on emission inventories, the benzene concentrations remain low (<0.1 ppbv) until the calculated trajectories move over southern Sweden, and peak over Copenhagen at ∼0.7 ppbv (Fig. 4, panel (b); see also Fig. S2 and S3†). Oxidant evolution, summarized in panel (a), shows a typical diurnal behaviour: OH level rises during the day. NO and NO_2_, if emission is low, are converted to the NO_3_ radical by O_3_. This process is suppressed during the daytime by photo-reactions: 
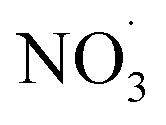
 and NO_2_ are photolyzed to NO_2_ and NO, respectively, which explains the source-independent increase in NO during daytime.

Benzene, due to its aromatic structure, is oxidized only by OH at a considerable rate.^42^ The resulting peroxy RO_2_ species drives the SOA formation during daytime *via* autoxidation chemistry (see “APRAM CHO” in Fig. 4b). Over Malmö and Copenhagen, the high NO concentrations partially suppress the autoxidation of peroxy radicals formed by OH oxidation of benzene, which results in less highly oxygenated organic molecules. The impact on aerosol mass yield and SOA composition is shown in the ESI (Fig. S4–S6).† As the air masses are transported away from the source (emissions of benzene and NO_*x*_), the chemical system shifts to less NO_*x*_-mediated autoxidation chemistry. The effect on SOA mass formed can be seen when comparing the situations “i”, “ii” and “iii” in Fig. 4, panel (b). Further, this is graphically illustrated in panel (d) which aims to depict the dependence of SOA mass yield on VOC (*i.e.*, benzene), OH and NO_*x*_.

There is a distinct increase in modeled SOA mass after sunset which appears low as benzene levels are low; however, the ΔSOA gets more apparent after the air parcels have passed over the Oresund region (*i.e. t* = 0 days in Fig. 4, panel (b)). During the first few hours after the sunset during days 1 and 2 downwind Malmö/Copenhagen, rapid 
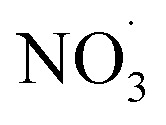
 oxidation of the intermediate MCM benzene oxidation product 4-nitrocatechol (C_6_H_5_O_4_N), which is formed and accumulates in the gas-phase during the daytime, contributes to the formation of a highly oxygenated di-nitrate RO_2_ species (C_6_H_5_O_11_N_2_). This results in substantial organonitrate SOA mass formation, composed mainly of organonitrate dimers (“CHON” Fig. 4, panel (b) and ESI Fig. S2 and S3, panel (b)†). Those species, although highly oxygenated, are not considered HOM (according to definition by Bianchi *et al.*^12^) as the formation does not include autoxidation steps. Note that this formation pathway remains to be investigated experimentally. Yet, it is entirely based on modeling results.

The trajectory model runs are contrasted by the more general picture of daytime benzene-SOA formation potential (see Fig. 4, panel (d)). The SOA mass yield is calculated applying a box model (ADCHAM, see Section 2.6 Numerical simulations: ADCHAM & ADCHEM applications for details) mimicking the daytime atmospheric conditions and a range of VOC and NO_*x*_ mixing ratios. Generally, predicted yields vary between less than 1% for high NO_*x*_ and low VOC conditions and up to 50% under extreme conditions (high VOC and low NO_*x*_). The computed mass yields show a slight increase with increasing NO as long as NO is low ([NO] < 0.1 ppbv). Above roughly 0.1 ppbv, the mass yield shows a distinct negative correlation with increasing NO. On the other hand, there is a positive correlation between the benzene turnover and yield. Note that these computed yields are highly dependent on OH concentration, despite being relatively insensitive regarding variations in seed aerosol properties, sunlight (neglecting the strong indirect effect *via* OH formation), methane concentration or O_3_ (ESI Fig. S7†). Further, note that the OH and O_3_ concentrations were set to constant values in these simulations in order to allow investigation of mass yields under various VOC/NO_*x*_ concentrations at a typical daytime OH level. This reflects the assumption that the VOC of interest is not dominating the OH sink. A strong variation in oxidants due to different VOC or NO_*x*_ levels would complicate the interpretation of individual data points as well as the comparability of mass yield results.

## 4 Discussion

### 4.1 Limitations of the mechanism development

The presented methodology aims to investigate the potential impact of autoxidation chemistry on (a) the gas-phase chemistry, (b) SOA formation in seeded flow reactor experiments, and (c) atmospheric SOA formation. Since the approach gathers information and data from various sources, caution is in order. In the following, the main limiting factors are discussed together with the arising consequences for the presented results.

#### 4.1.1 Main assumptions: the autoAPRAM-fw, the chemical reaction types and structures formed

The autoAPRAM-fw creates chemical reaction schemes considering a set of predefined chemical reaction types. The products formed within the schemes are characterized by means of SMILES-strings. Since the list of reaction types is limited, the autoAPRAM-fw does not cover the full variability of isomers. Consequently, some isomers are represented by a single structure. The reason to assign SMILES structures is to estimate vapor pressures to derive the partitioning to the particle phase and to the chamber walls. To test the potential impact of this approximation on the SOA mass formed, we derived a variety of RO_2_ isomers that may form in the chemistry (see Fig. S12†). All RO_2_ species (including isomers) are passed to the autoAPRAM-fw and the resulting ensemble of isomers is sorted by their vapor pressures (lowest, average, highest) and the seeded experiment (Fig. 3a) is repeated (see Fig. S18†) to visualize the potential effect of isomeric variability on the vapor pressure.

Another consequence of the underrepresentation of isomeric variability is the fact that some reactions suggested may not be possible with regard to their chemical structure.

#### 4.1.2 Limitations regarding the rate coefficients assigned (see Section 2.3)

In this work we relate the timely evolution of the atomic mass spectrum to physical and chemical processes. This allows the parameters of the processes (*e.g.* chemical rate coefficients or first order wall loss of chemical species) to be quantified. Since several reaction products may contribute to a mass peak observed, the system is underconstrained when a mass spectrum at time *x* is compared to a simulated mass spectrum at the same model-time. For example, reactions (R3c), (R4a), (R5c), (R7*) and (R8*) may form isomers. Typically, a single reaction term is dominant (*e.g.* high HO_2_ regime) which allows its rate coefficient to be quantified. Meanwhile, the other contributing terms' rate coefficients are subject to considerable uncertainty. In the present case of benzene, the rate coefficients have been optimized for reproducing the flow tube experiments and reactions (R3a)–(R3c) are considered afterwards. As a result, we derived a potential solution for the ensemble of rate coefficients which satisfies the conditions in the flow tube and the elevated NO_*x*_ conditions in the JPAC chamber. However, reaction branches that may get dominant in a different environment may be ill-quantified (*e.g.* self-determination of autoxidation by reaction (R7*)).

Limitations regarding the saturation vapor pressures assigned can be found in the ESI.†

#### 4.1.3 Limitations regarding the saturation vapor pressures assigned (see Section 2.5)

Limitations regarding the choice of method to predict the saturation vapor pressure of a molecule are manyfold. Various methods to predict this quantity based on atomic composition, functional groups or the whole structure and its stereochemical distribution exist. However, since the isomeric distribution of highly oxygenated organic molecules is beyond our understanding for any chemical system, a standardized set of species to investigate is not available. As a result, we miss a clear idea to choose a representation. This is why several methods have been considered in simulations of all SOA-forming laboratory experiments (see Fig. 3).

### 4.2 Limitations of the numerical simulations

In this section focus is put on the limitations of SOA simulations arising in context with the chemical scheme proposed. General limitations of the numerical simulations of aerosol dynamics using ADCHAM or ADCHEM are discussed elsewhere.^21,50,51^

Limited knowledge on the partitioning of molecules to the chamber walls is a challenge. Since the benzene structures are covered by the autoAPRAM-fw feature 5 and more oxygen atoms, a first order loss term was applied (see Section 2.6.2). The wall partitioning is of great relevance in chamber simulations as it directly affects the production rates inferred: the higher the wall loss rate is, the higher is the reaction rate coefficient forming the depositing species in order to explain the observed mass-peak hight.

In the atmospheric simulations, the main limitations related to autoxidation chemistry are:

- The reaction type (R5) in which the peroxy radical of interest reacts with the pool of peroxy radicals. Rate coefficients for this reaction have been derived in the flow tube experiment which comprises “hotter” (*i.e.*, concentrations of OH and HO_2_ are much higher than atmospheric ones) chemistry than the atmosphere and very different timescales (*i.e.*, slow reactions underrepresented compared to the atmosphere).

- While the reaction type (R5) may be overrepresented in the atmospheric simulations, the reaction types (R7*)–(R9*) may gain more importance in the atmosphere compared to the chamber.

Another limitation is the temperature dependence, particularly of the RO_2_ autoxidation reaction, which is considered exponential. In this work the temperature dependence is neglected due to lack of data.

### 4.3 Utility of the autoAPRAM-fw

Despite the limitations listed in previous sections, there is broad utility of the approach drafted. The autoAPRAM-fw may be used to:

(1) Produce atomic peak lists for a set of predefined (autoxidation) reactions, *e.g.*, to be used in the analysis of CIMS spectra. The automated nature of the framework allows large numbers of peaks to be provided quickly. The product description allows isomers and reaction pathways to be considered.

(2) Create, within seconds, sets of chemical reactions governing autoxidation based on selection of the parent VOC and reaction types chosen. Rate coefficients need to be provided (pure guess or based on SAR).

(3) Assign potential rate coefficients to the schemes created based on experimental data as described in Section 2.3. This process requires substantial working time. However, this limiting factor will be overcome soon as we are currently evaluating two approaches to automatically assign potential solutions for rate coefficients from experimental data: (a) a random-forest based method, run on a computer cluster and (b) an analytically-based approach providing roughly 10^3^ rate coefficients per second during evaluation of the chemical scheme against CIMS data.

(4) Characterise the products formed in the autoxidation scheme by means of: (a) atomic composition, (b) functional groups and (c) likely SMILE-structures. This allows various methods to be considered to derive saturation vapor pressures.

(5) Set up an autoxidation chemistry scheme for any parent VOC. The approach is not limited to benzene.

(6) Implement new reaction types to consider new theoretical/experimental findings.

(7) Consider an increased isomeric variety of input radicals. In the benzene example, a single peroxy radical species represents all peroxy radical isomers (*i.e.*, the RO_2_ with 7 oxygen atoms: Bzo_RO2_O7 describes RO_2_ isomers of composition C_6_H_7_O_7_).

(8) Couple autoxidation chemistry schemes to MCM and solve them with an aerosol-dynamics and chemistry model. The resulting model setup is capable of investigating the mechanistic behaviour of the chemistry in detail. A similar degree of detail is not met in any other approach aiming to be implemented in aerosol-dynamics and chemistry models. Still, the suggested method is much more lumped than other approaches aiming to cover the full autoxidation chemistry.^72^

To summarize, the presented approach does not aim to be mechanistic or predictive with regard to structure-dependent reaction pathways. Further, the reaction rate coefficients applied are not determined from structure–activity relationships. The autoAPRAM-fw serves to create reaction schemes to be related to experimental data in order to quantify the rate coefficients of reaction types that explain the observed rearrangement of the CIMS peaks. SARs are considered when comparing the rate coefficients found to previous results from similar systems. This is done in order to check the plausibility of their magnitude. Thus, the proposed autoxidation scheme for benzene represents a single, potential solution. It allows observed CIMS-data evolution as well as SOA formation to be reproduced while proposing realistic (according to the literature) reaction parameters.

## 5 Conclusion

Autoxidation chemistry is a key for understanding the atmospheric source of many multiply functionalized molecules and their potential partition to the condensed phase.^14^ Applying reaction-rules of alkoxy- and peroxy radical chemistry enables sets of chemical reaction equations to be compiled in an almost automated fashion using the autoAPRAM-fw.

Currently, the fitting of reaction rate coefficients to allow for reproduction of the experimentally obtained gas phase species mass spectrum is a limiting factor: it requires considerable manpower (of the order of weeks for a system like benzene). To overcome this, we are currently exploring two approaches: (1) a randomized search for good fits of rate coefficients; and (2) an analytical solution to obtain rate coefficients from stationary and steady state-like experimental conditions. Approach (1) requires substantial computational effort while the analytical method can be run on a personal computer within seconds.

In the present work we show that these reactions and their rate coefficients may be constrained against experimental data from the flow tube and chamber to reproduce the part of the mass spectrum covered by HOM species within the limits of calculated error (typically less than 10% for relative concentrations of HOM species), including the distribution of peroxy radicals (see Fig. 2). Thereby, we found a potential relationship (see eqn (1)) between the rate coefficient and the molecular mass for the ROOR formation *via* reaction (R5b). Predicting the species vapor pressure by group contribution methods proves successful to reproduce observed mass yields in seeded experiments found in the low NO regime (experimental: 40%^66^ and 37%^64^*vs.* simulated: 42% (range: 12% to 71%) and 28% (range: 14% to 42%)), regardless of the experiment type. In contrast, formation of condensed phase species is under-predicted under high NO conditions (experimental: 26% *vs.* simulated: 7%). Potential reasons for this discrepancy are diverse: (a) the present approach neglects the oxidation of closed shell autoAPRAM species. Thus, if terminating reactions are dominating, the propagation of radical chemistry may be under-represented. (b) The prediction of the nitrate species' vapor pressure in the group contribution methods, generally, is considered less accurate compared to non-nitrated species as the experimental data is scarce.^68^ Reasons for scarce experimental data are, besides others, their high reactivity which makes the handling difficult. (c) The high reactivity of NO_*x*_ species affects oxidation experiments in chambers and flow tubes as well. Accordingly, availability of high resolution CIMS data for nitrate species during VOC oxidation experiments is very limited which exacerbates model constraint. (d) Formation of highly functionalized organic species in the presence of high NO mixing ratios likely does not proceed *via* RO_2_ autoxidation as NO strongly limits the peroxy radical lifetime (ESI Fig. S8†). Thus, more fundamental work on nitrated species autoxidation and properties of the respective products will help to improve the model representation. Further note that reactive uptake of the oxidation products at the surfaces of the condensed phase is not modeled explicitly. Accordingly, the calculated SOA mass may be underrepresented in case reactive uptake is of importance, as could be the case for the product organonitrates. Another species group potentially contributing to reactive uptake is epoxides: they may form *via* secondary oxidation of aromatic species. At the aerosol surface they transform into less volatile species *via* acid-catalyzed ring-opening reactions.^73^

Clearly, the molecular structures and their isomeric distribution are subject to speculation. Likely, this situation will remain for an unforeseen time. Similarly, the exact retrieval of saturation vapor pressures for given molecule structures is difficult. However, based on the set of potential peroxy radical isomers (see Fig. S12†) and the methods applied to derive the saturation vapor pressure (see Section 2.5 “Deriving the saturation vapor pressure”), we found that the description of autoxidation and its effect on the molecular structures is still crucial for modeling SOA formation from benzene. This is valid, independent of the choice of underlying RO_2_ structures and independent of the choice of *p*_sat_-derivation method.

The presence of high mixing ratios of NO and HO_2_ poses additional challenges as their nitrate and ROOH yield by NO and H addition, respectively, is suggested to be very low for peroxy radicals attached to a C-ring.^42^ The yields of closed shell species during the RO_2_ reaction with NO and HO_2_ are predicted to be, besides the latter being highly uncertain, of the order of 0.1% and 1% respectively.^42^ Thus, the species allow for efficient NO_*x*_ and, likely but less certain, HO_*x*_ cycling. However, the molecular rearrangements of resulting alkoxy species are hard to predict.^74,75^ In the present chemistry scheme, nitrate and ROOH yields in the range of 0.2% to 4% and 0.5% to 60% are applied. While the yields of RONO_2_ species can be determined from CIMS data, the ROOH yields are less distinct as they can't be distinguished from species formed *via* other bi- or uni-molecular reactions, featuring the same composition.

Clearly, the model predicts potential, high mass yields of up to 55% from benzene oxidation under atmospheric-like daytime conditions (*i.e.* low 
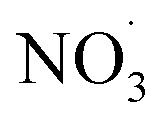
 concentration; see Fig. 4, panel (d)). Additional SOA formation is predicted from 
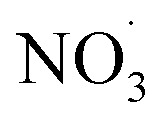
 oxidation of benzene–OH oxidation products shortly after sunset, resulting in the formation of highly oxygenated organonitrates. The modeled SOA mass yield positively correlates with increasing benzene turnover. This finding is supported by recent work showing a positive correlation between benzene turnover and HOM formation.^66^ The effect of NO on autoxidation is less uniform: at NO concentrations less than about 0.1 ppbv, the yield positively correlates with NO, while at higher nitrogen oxide levels, there is a negative correlation. A low NO concentration, due to high yield of alkoxy radicals, may speed up autoxidation.^76^ Note that RO H-shift rates are suggested to be orders of magnitude faster (about 10^3^ to 10^7^ s^−1^)^74^ compared to rate coefficients for peroxy radical H-shifts which are reportedly below 1 s^−1^ with few faster exceptions.^16,19,77^ H-shift rates for peroxy radicals in the range from 0.01 s^−1^ to 1.5 s^−1^ are applied in the current benzene-autoxidation scheme (see ESI section “AutoAPRAM – benzene scheme”†). If NO concentration exceeds the level of 0.1 ppbv, it starts to scavenge the RO_2_ pool which forms the basis for peroxy radical autoxidation reactions and low-volatility dimer formation (see ESI Fig. S8†). For both RO and RO_2_ species, H-shift rates strongly depend on the substitution and span of the H-shift.^78^

A close to realistic atmospheric transport simulation, applying the complete chemistry scheme developed, suggests that condensable species formed by benzene autoxidation chemistry (added on top of the MCM chemistry scheme by applying the autoAPRAM-fw) can have a significant share of the anthropogenic contribution to ambient organic aerosol mass. This fraction (up to 20%), likely, is over-predicted, as autoxidation chemistry, in the model, is still limited to a few VOC species: benzene, which is the focus of the work, as well as α-pinene, β-pinene, limonene and carene.^21^ Additionally, the simulations for Malmö, Sweden, cover a period in April with cold Arctic air masses, which naturally limits the contribution of biogenic species.

Apparent mass yields from daytime autoxidation chemistry (determined from ADCHEM simulations) are similar to parametric simulations under comparable conditions (yields of about 20–52%; ESI Fig. S4–S6† and <55%, respectively). There are differences between apparent mass yields in the atmospheric runs and mass yield from the parametric simulations: the parametric runs consider daytime conditions only. As a consequence the 
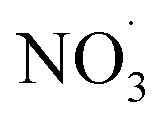
 radical levels remain low and OH is the dominating oxidant. In the atmospheric simulations, OH oxidation-products of benzene accumulate during the day and are oxidized by 
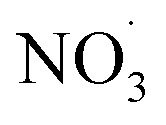
 radicals at dawn (see ESI Fig. S9†). Since OH is low while new SOA forms, the mass yield (ΔSOA/Δbenzene) can get very high as benzene is only oxidized by OH (Xu *et al.*, 2020). As a result, apparent mass yields of up to 1000% are modeled for short periods of time. The simulated 
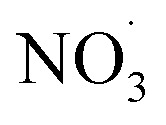
 oxidation of benzene-products seems to question the use of a general benzene mass yield based on OH oxidation experiments. Note that the benzene-SOA formation initialized by nitrate radicals is a model prediction and remains to be examined experimentally.

Direct comparison between atmospheric (simulation) data and mass yield simulations is generally difficult as characterizing parameters don't fully match (*e.g.*, temperature, relative humidity). However, clearly, benzene and its intermediate oxidation products are oxidized under changing NO_*x*_, OH˙ and 
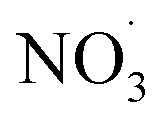
 levels which on the one hand affects the formation of HOM species (in particular the dimer formation) and on the other hand impacts on the nitrogen-containing species' contribution to SOA. As the air masses are transported away from hotspots of anthropogenic activity (*i.e.* emission sources of benzene and NO_*x*_), the conversion of benzene to SOA *via* OH oxidation becomes more effective due to a shift in the chemical regime towards less NO_*x*_ mediated chemistry (see Fig. 4). The formation of SOA, during daytime, is as expected based on experimental findings.^64,66^ In contrast, the increase of 
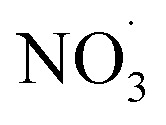
 at dusk results in a distinct and timely limited increase in modeled SOA formation. This effect has not yet been investigated experimentally.

The authors assume that considering autoxidation chemistry for additional VOC species will most likely increase the formation of SOA and reduce the share of individual components. However, bear in mind that mixtures of VOC species may decrease the individual SOA yield *via* scavenging the oxidants or by forming higher volatility RO_2_ + RO_2_ reaction products as shown for the α-pinene–isoprene system.^65^ On the other hand, it has been shown recently that VOC mixtures may also increase mass yields significantly: Faiola *et al.* found augmentation of the aerosol formation by roughly 50–130% from real monoterpene–sesquiterpene mixtures emitted by stressed Scots pine trees.^79^ Consequently, we think that a mechanistic representation of autoxidation chemistry, to some extent, is essential to improve the prognostic capacity in experiments and under atmospheric conditions. This includes the construction of models able to capture various aspects observed experimentally. Further, upon successful reproduction of experiments by means of modelling, reduction of the code to a reduced formal extent is inevitable to allow application in large scale models.^80^ A mechanistic model-representation of key observations from experiments will most likely be successful with regard to predictions in future changing atmospheres, whereas empirically based approaches may fail as a result of prevailing conditions not resembling those in their empirical foundation.

## Data availability

Data from the flow tube, JPAC chamber (high/low NO_*x*_) and CALTECH chamber (high/low NO_*x*_) can be accessed online (https://doi.org/10.5281/zenodo.8087267). Data from ADCHEM atmospheric simulations are available upon request (office@pi-numerics.com).

## Author contributions

LP, PR, MR, TK and MiB designed the research; LP, PR, MR, NH, CD, OG, CX, PZ, PC, BF, TGA, MeB, TK and MiB conducted the research; LP, PR, MR, MeB, MiB, NH, and OG analyzed the data; LP and PR developed the models; LP, PR, MR and MiB wrote the paper.

## Conflicts of interest

There are no conflicts to declare.

## Supplementary Material

EA-004-D4EA00054D-s001
