# Supplementary material for: Towards automated inclusion of autoxidation chemistry in models: from precursors to atmospheric implications
Source: Environ Sci Atmos. 2024 Jul 9;4(8):879–96. doi: 10.1039/d4ea00054d (PMC11307592; doi:10.1039/d4ea00054d)
Supplement: EA-004-D4EA00054D-s001 [file EA-004-D4EA00054D-s001.pdf]

Electronic Supplementary Material (ESI) for Environmental Science: Atmospheres.
This journal is © The Royal Society of Chemistry 2024

Supplementary text

Interpreting ion count data from CIMS measurements

In this study, we compared the output of the model to the benzene oxidation product distribution measured with nitrate ion-based chemical ionization mass spectrometer (NO_3^- - CIMS[1]). The product spectra for low-NO_x conditions were taken from Molteni et al.[2], while for the high NO_x conditions from Garmash et al..[3]

While NO_3^- - CIMS is known to be selectively sensitive towards highly oxygenated multifunctional molecules and radicals formed in volatile organic compound (VOC) oxidation, it is almost completely insensitive toward the least oxygenated species and there are further limitations associated with the CIMS data interpretation. Firstly, the quantification of the detected species is challenging, due to the absence of calibration standards. Secondly, in routine operation, mass spectrometers cannot separate isomers and only exact chemical composition can be resolved. In this section, the focus shall be on the interpretation of ion count data of multifunctional molecules from NO_3^- CIMS.

To be detected by the MS instrument, the molecules of interest are first charged, and the charging in CIMS is done via ion – molecule reactions (ion, here: NO_3^-). As described by Hyttinen et al., (2018), several processes occur in the ion-molecule reaction region within the CIMS:

SR1) Ionization may take place via deprotonation of the target molecule (RH) by the reagent ion (Q^-).

SR2) The reagent ion can attach to the RH to form ion-molecule clusters.

SR3) The reagent ions may form stable reagent ion "dimers" ($QH(Q^-)$) which may ionize the sample molecule via ligand exchange.

SR4) Clustering may also happen with the reagent ion dimer.

SR5) The ionization may also happen via ligand exchange reaction between the sample molecule and hydrates of the clustering ions.

SR6) Interaction of clustering ion and sampling molecule may also result in fragmentation.

$$RH + Q^- \rightarrow R^- + QH \quad \text{(SR1)}$$
$$RH + Q^- \rightarrow (RH)Q^- \quad \text{(SR2)}$$
$$RH + QH(Q^-) \rightarrow QH + RH(Q^-) \quad \text{(SR3)}$$
$$RH + QH(Q^-) \rightarrow (RH)QH(Q^-) \quad \text{(SR4)}$$
$$RH + (H_2O)_nQ^- \rightarrow nH_2O + RH(Q^-) \quad \text{(SR5)}$$
$$RH + Q^- \rightarrow \text{fragmentation products} \quad \text{(SR6)}$$

As a given molecule or a radical can be detected as different ions (R^-, $RH(Q^-)$, $(RH)QH(Q^-)$), it may be difficult to identify the composition of the parent species before it was ionized. Similarly, it is difficult to determine all signals related to one product. This problem is especially pronounced in high-NO_x experiments, where formed oxidation products will contain multiple nitrogen and oxygen atoms. As a result, oxidation products charged by reagent ion dimer (SR4) can be confused with another N-containing oxidation product charged by reagent ion monomer (SR2).

In the present work, products observed in high NO_x oxidation of benzene in JPAC chamber[3] were interpreted based on their timing. If signals of those ions correlated with $R^2 > 0.9$, they were classified as the same sample molecule. For example, ions at m/z 201 and 264 were identified as $C_6H_5NO_2{*}NO_3^-$ and $C_6H_5NO_2{*}HNO_3{*}NO_3^-$ respectively. Signals of these ions varied closely during the experiments with $R^2 = 0.995$ (see Fig S17). In this case, these ions were classified as the same molecule (nitrophenol) detected via ionization reactions (SR3) and (SR4).[3] Similar analysis was performed on other ions.

The CIMS-signal conversion to a mixing ratio or number concentration is approached in different ways: For the simulations of flow tube results (data obtained from Molteni et al.[2]) the original data is reported already in cm^{-3}, and thus no conversion is needed. For the simulations of JPAC chamber experiments, a conversion factor of $1.2*10^{11}$ cm^{-3} for "fraction of total signal" to cm^{-3} conversion is used. This conversion factor was derived from reproducing benzene oxidation mass spectra (under low NO_x conditions; see Garmash et al.[3] - SI) in JPAC chamber by means of modelling. The conversion factor of $1.2*10^{11}$ cm^{-3} best converted from experimental data to the simulated spectra.

High NO_x conditions
Benzene OH oxidation in the presence of high levels of NO_x is summarized in Fig S15. Panel A depicts the simulated and experimental[3] (nitrate CIMS) distribution of molecular mass. The experimental data set serves to constrain the branching ratio for reaction R3 to either form the closed shell $RONO_2$ species (R3a; see main manuscript) or the unstable alkoxy radical (R3b; see main manuscript). As recently reported[4] for the benzene system, the branching produces only a small share of nitrate species (~ 0.2% for bicyclic peroxy radicals and ~ 0.9% for other peroxy radicals containing 6 carbon atoms). In the present work, nitrate yields from reaction R3 range from 0.1% (bicyclic peroxy radical) to 4%. Accordingly, unless the overall reaction rate constant $k_{RO2\text{-}NO}$ for reaction of RO_2 species with NO is overestimated, a large amount of RO species are formed. We explored the potential of the latter path to explain experimentally observed mass spectra and, subsequently the impact on the SOA forming potential of the species formed that way. The reproduction of observed nitrate mass peaks is unproblematic, due to its small share in reaction R3. However, the computed dimer distribution (panel B) suggests underestimation of its main precursor, the RO_2 species. This problem cannot be overcome, even if the yield of reaction R2 clearly dominates over R6 and R8 and literally all alkoxy radicals are transformed to peroxy radicals via autoxidation.

Investigation of the partition to the particle phase is shown in (Fig 3, panel E). The predicted and observed SOA formation differ with increasing hydrocarbon reacted. While the simulated yield can be characterized as constant (roughly 7%), the experimental SOA formation seems to increase the more of the precursor hydrocarbon reacts. Note that the experimental SOA formation data from literature was cut at 100 µg m^{-3} as the OH precursor seems to be almost depleted. The additional oxidation and SOA formation is likely driven by NO_x/HO_x chemistry in the presence of UV light. This does not represent a well-defined chemical system which, accordingly, is not considered for analysis. Consequently, we further question the suggested mass yield of roughly 37% to be fully explained by the OH oxidation of benzene in the presence of NO_x.

Distribution of saturation vapor pressures from group contribution methods
Deriving the saturation vapor pressure function for a specific standard substance can be done experimentally. However, many atmospherically relevant species feature unknown structures, are short lived or highly reactive. Additionally, the number of molecular structures originating from oxidation of a single precursor VOC can be huge.[5,6]
In the present work, a few chosen molecules are investigated by means of quantum chemical calculations using the COSMO-RS model[7–9]. This involves conformer sampling for the considered structure. The conformers showing intramolecular hydrogen bonds are removed from further analysis as they reportedly bias the results[10]. The COSMO*conf* program[11] is used to optimize the geometries and derive the screening charge surface of each conformer considered. This forms the input for condensed phase property computation by the COSMO*therm* program[12], parameterization BP_TZVPD_FINE_19. A detailed description of the procedure can be found elsewhere.[13] This procedure requires significant computational ressources for a single structure being analysed. In order to overcome this challenge, group contribution methods may be consulted. Many of these

methods exist featuring differing accuracy.[14] Group contribution methods applied in this work, were accessed via the online UManSysProp tool [15]. Their performance is discussed in O'Meara et al. [16].
In the present work, saturation vapor pressures were derived with three different group contribution methods for all species: EVAPORATION,[17] NANNOOLAL[18] and MYRDAL/YALKOWSKI[19]. Resulting saturation vapor pressure values at fixed temperature are shown in Fig S13. The methods were chosen based on an assessment which suggested NANNOOLAL to predict the normal boiling point best while EVAPORATION and MYRDAL/YALKOWSKI reproduce SOA mass loadings most accurately amongst all investigated methods.[14]
Fig S14 depicts computed p_{sat} using COSMO-RS in comparison to results from the group contribution methods. There is a considerable variation in the prediction of p_{sat} depending on the method chosen: absolute standard deviations in orders of magnitude [Pa] (psat[group method] - psat[COSMO-RS] at room temperature) for the species computed are roughly: +/- 2.2 (EVAPORATION), +/- 3.9 (NANNOOLAL) and +/- 2.6 (MYRDAL/YALKOWSKI). All group contribution methods tend to overestimate vapor pressures for the species considered. Mean overestimation in orders of magnitude [Pa] are +0.8 (EVAPORATION), +0.5 (NANNOOLAL) and +5.3 (MYRDAL/YALKOWSKI).
For the non-nitrates, EVAPORATION and NANNOOLAL show the closest agreement with COSMO-RS. MYRDAL/YALKOWSKI seem to consistently produce too high psat compared to COSMO-RS. In the cases of closed shell nitrogen containing species, EVAPORATION shows least deviation from COSMO-RS. In the case of dimers, only two species have been analyzed due to the significant computational effort required. For both molecules, EVAPORATION overestimates the saturation vapor pressure by roughly one order of magnitude compared to the COSMO-RS. However, this may not be relevant in the atmosphere as the species will irreversibly partition to the particle phase due to their extremely low vapor pressure. Thus, we conclude that EVAPORATION is, based on the limited set of comparison with COSMO-RS results, the best method in the present case of benzene oxidation.
Limitations regarding the choice of method to predict the saturation vapor pressure of a molecule are manyfold.[16] Since the isomeric distribution of highly oxygenated organic molecules is beyond our understanding for any chemical system, a standardized set of species to investigate is not available. As a result, we miss a clear idea to choose a representation. This is why several methods have been considered in simulations of all SOA-forming laboratory experiments (see Fig 3).

Model input specifications

Main model inputs for the simulations reported are listed below for a) the flow tube simulations (table ST3), b) the JPAC chamber runs (table ST4 and ST5), c) the Caltech chamber runs (ST6) and d) the atmospheric yield study (ST2). The inputs for the atmospheric trajectory runs are less appropriate to be summarized in a table, however, it can be provided by the corresponding author (LP) upon request.

Description of parametric yield calculations

In order to approach atmospherically relevant conditions, methane, CO and sunlight are considered. The sunlight intensity, based on experimentally determined spectrum, was set to a value resulting in an OH level of $2*10^6$ cm^{-3} in the presence of 200 ppb_V of CO and 2 ppm of methane, the two largest atmospheric OH sinks. When conducting the simulations, the OH level was fixed in order to avoid bias by changing the VOC level. Losses of seed aerosol particles, VOC species or their products are neglected in these simulations.
Benzene and NO_x concentrations are varied over a wide range covering observations from remote areas to strongly polluted regions.[20,21] OH concentrations are set to a moderate level and are kept constant for 10 hours. In case the mass yield, within this period, does not approach a constant or maximum value, respectively, OH is set to zero and simulations are continued for another 2 hours.

Exemplary mass yield evolution simulations are depicted by Supplementary Fig S16. In panel A ("sim8"), the maximum mass yield is met at 2 h to 4.5 h after initiating the OH oxidation. Random fluctuations in the yield curve are numerical artifacts attributable to limited accuracy of integration time steps which were set to 5 s to limit computational costs. However, note that the final yield values are not affected. On the one hand, the fluctuations do not introduce a systematic offset, on the other hand yield values are averaged over a certain period, indicated by a purple square, to remove the fluctuations where necessary. Occasionally, no yield maximum is found within 10 hours of benzene oxidation, as shown by panel B. In this case, the yield value at t = 10 hours represents the maximum mass yield.

Clearly, the data generated does not cover all possible conditions. Though, the seed aerosol mass and diameter, as well as the O_3 concentration, under the studied conditions, do not largely impact the mass yield. However, the OH level strongly impacts the result. See supporting information for effects of varying O_3, seed aerosol mass, seed aerosol surface and OH for two conditions: one characterized by high mass yield (0.1 ppb_V NO_x/ 10 ppb_V benzene) and the other characterized by low mass yield (10 ppb_V NO_x and 0.1 ppb_V benzene).

Note that the NO_x values can be converted to NO levels by multiplication times 1/11 as NO_2:NO is set to 10:1.

Specifications of autoAPRAM-benzene species

Basic molecular properties (in a format: name /C/H/O/N/ structure) for all APRAM species used in the model simulations are listed in table ST7.

autoAPRAM-fw – benzene scheme

Reaction rate coefficients are given in s^{-1} for reactions {1} to {7} and reaction types R1, R2 and R6 to R9 (see main manuscript section 2.2 for more information on the reaction types considered). All other rate coefficients are given in $cm^3 molecules^{-1} s^{-1}$. autoAPRAM-fw uses simple rate coefficients KDEC, KRO2NO and KRO2HO2 from MCM v3.3.1:

KDEC = 1E-6

KRO2NO = 2.7E-12*EXP(360/TEMP)

KRO2HO2 = 2.91E-13*EXP(1300/TEMP)

Reaction types marked by "Reaction (RX)" refer to reactions introduced in section "Setting up the gas phase chemistry". Reactions {1} to {7} represent the initiation of autoAPRAM-fw RO2 species from MCM (i.e., the reactions where MCM species form autoAPRAM-fw peroxy radicals). The reactions {8} to {934} represent the autoxidation scheme for benzene created by the autoAPRAM-fw.

autoAPRAM-fw initiation from MCM

{1} BZBIPERO2 -> BZo_RO2_O7 0.32

{2} BZBIPERO -> BZeo_RO2_O6 0.1*KDEC

{3} BZBIPERO2 -> C5_RO2_O6 + CO 0.022

{4} BZEMUCO2 -> BZeo_RO2_O8 0.07

{5} BZEMUCO2 -> C5_RO2_O7 + CO: 0.25

{6} PHENO2 -> BZeo_RO2_O8 0.07

{7} PHENO2 -> C5_RO2_O7 + CO: 0.25

Reaction (R1)

Autoxidation: RO_2 -> H-shift + O2 addition -> RO_2

{8} BZo_RO2_O7 -> BZo_RO2_O9 1.5

{9} BZo_RO2_O9 -> BZo_RO2_O11 0.45

{10} BZeo_RO2_O6 -> BZeo_RO2_O8 0.1

{11}	BZeo_RO2_O8 -> BZeo_RO2_O10	0.01

Reaction (R2)

Alkoxy radical autoxidation: RO -> RO_2

{12}	BZo2_RO_O6 -> BZeo_RO2_O8	KDEC*0.90
{13}	BZo2_RO_O8 -> BZeo_RO2_O10	KDEC*0.85
{14}	BZeo2_RO_O5 -> BZo_RO2_O7	KDEC*0.90
{15}	BZeo2_RO_O7 -> BZo_RO2_O9	KDEC*0.90
{16}	BZeo2_RO_O9 -> BZo_RO2_O11	KDEC*0.40
{17}	BZo_RO_O6 -> BZeo_RO2_O8	KDEC*0.2
{18}	BZo_RO_O8 -> BZeo_RO2_O10	KDEC*0.1
{19}	BZeo_RO_O5 -> BZo_RO2_O7	KDEC*0.45
{20}	BZeo_RO_O7 -> BZo_RO2_O9	KDEC*0.35
{21}	BZeo_RO_O9 -> BZo_RO2_O11	KDEC*0.45
{22}	C5_RO_O5 -> C5_RO2_O7	KDEC*0.90
{23}	C5_RO_O6 -> C5_RO2_O8	KDEC*0.90
{24}	C5_RO_O7 -> C5_RO2_O9	KDEC*0.85
{25}	C5_RO_O8 -> C5_RO2_O10	KDEC*0.70

Reaction (R3a)

Peroxy radicals reacting with NO to form $RONO_2$: -> RO_2 + NO -> $RONO_2$

{26}	BZo_RO2_O7 + NO -> BZo_O5_NO3	KRO2NO*0.009
{27}	BZo_RO2_O9 + NO -> BZo_O7_NO3	KRO2NO*0.005
{28}	BZo_RO2_O11 + NO -> BZo_O9_NO3	KRO2NO*0.12
{29}	BZeo_RO2_O6 + NO -> BZeo_O4_NO3	KRO2NO*0.002
{30}	BZeo_RO2_O8 + NO -> BZeo_O6_NO3	KRO2NO*0.006
{31}	BZeo_RO2_O10 + NO -> BZeo_O8_NO3	KRO2NO*0.01
{32}	C5_RO2_O6 + NO -> C5e_O4_NO3	KRO2NO*0.04
{33}	C5_RO2_O7 + NO -> C5_O5_NO3	KRO2NO*0.004
{34}	C5_RO2_O8 + NO -> C5e_O6_NO3	KRO2NO*0.008
{35}	C5_RO2_O9 + NO -> C5_O7_NO3	KRO2NO*0.02
{36}	C5_RO2_O10 + NO -> C5e_O8_NO3	KRO2NO*0.02

Reaction (R3b)

Peroxy radicals reacting with NO to form RO: RO_2 + NO -> RO + NO_2

{37}	BZo_RO2_O7 + NO -> BZo2_RO_O6 + NO2	KRO2NO*0.897
{38}	BZo_RO2_O9 + NO -> BZo2_RO_O8 + NO2	KRO2NO*0.978
{39}	BZo_RO2_O11 + NO -> BZo2_RO_O10 + NO2	KRO2NO*0.93
{40}	BZeo_RO2_O6 + NO -> BZeo2_RO_O5 + NO2	KRO2NO*0.898
{41}	BZeo_RO2_O8 + NO -> BZeo2_RO_O7 + NO2	KRO2NO*0.894
{42}	BZeo_RO2_O10 + NO -> BZeo2_RO_O9 + NO2	KRO2NO*0.59
{43}	C5_RO2_O6 + NO -> C5_RO_O5 + NO2	KRO2NO*0.96
{44}	C5_RO2_O7 + NO -> C5_RO_O6 + NO2	KRO2NO*0.998
{45}	C5_RO2_O8 + NO -> C5_RO_O7 + NO2	KRO2NO*0.996
{46}	C5_RO2_O9 + NO -> C5_RO_O8 + NO2	KRO2NO*0.98
{47}	C5_RO2_O10 + NO -> C5_RO_O9 + NO2	KRO2NO*0.98

Reaction (R3c)

Peroxy radicals reacting with NO to form RCHO: RO_2 + NO -> RCHO + NO_2 + HO_2

more detailed RO_2 + NO -> RO + NO_2 fast abstraction of H by O_2 eliminating of HO_2: RCHO + HO_2

{48} BZo_RO2_O7 + NO -> BZo_O5_O + NO2 + HO2 KRO2NO*0.1
{49} BZo_RO2_O9 + NO -> BZo_O7_O + NO2 + HO2 KRO2NO*0.02
{50} BZo_RO2_O11 + NO -> BZo_O9_O + NO2 + HO2 KRO2NO*0.03
{51} BZeo_RO2_O6 + NO -> BZeo_O4_O + NO2 + HO2 KRO2NO*0.1
{52} BZeo_RO2_O8 + NO -> BZeo_O6_O + NO2 + HO2 KRO2NO*0.1
{53} BZeo_RO2_O10 + NO -> BZeo_O8_O + NO2 + HO2 KRO2NO*0.4

Reaction (R4a)

Peroxy radicals reacting with HO2 forming closed shell species: RO_2 + HO_2 -> ROOH

{54} BZo_RO2_O7 + HO2 -> BZo_O5_OOH KRO2HO2*0.77*0.01
{55} BZo_RO2_O9 + HO2 -> BZo_O7_OOH KRO2HO2*0.77*0.06
{56} BZo_RO2_O11 + HO2 -> BZo_O9_OOH KRO2HO2*0.77*0.2
{57} BZeo_RO2_O6 + HO2 -> BZeo_O4_OOH KRO2HO2*0.77*0.6
{58} BZeo_RO2_O8 + HO2 -> BZeo_O6_OOH KRO2HO2*0.77*0.04
{59} BZeo_RO2_O10 + HO2 -> BZeo_O8_OOH KRO2HO2*0.77*0.55
{60} C5_RO2_O6 + HO2 -> C5e_O4_OOH KRO2HO2*0.706*0.005
{61} C5_RO2_O7 + HO2 -> C5_O5_OOH KRO2HO2*0.706*0.3
{62} C5_RO2_O8 + HO2 -> C5e_O6_OOH KRO2HO2*0.706*0.6
{63} C5_RO2_O9 + HO2 -> C5_O7_OOH KRO2HO2*0.706*0.1
{64} C5_RO2_O10 + HO2 -> C5e_O8_OOH KRO2HO2*0.706*0.6

Reaction (R4b)

Peroxy radicals reacting with HO_2 forming RO + O_2 + OH:

{65} BZo_RO2_O7 + HO2 -> BZo_RO_O6 + OH KRO2HO2*0.99*0.77
{66} BZo_RO2_O9 + HO2 -> BZo_RO_O8 + OH: KRO2HO2*0.92*0.77
{67} BZo_RO2_O11 + HO2 -> BZo_RO_O10 + OH: KRO2HO2*0.85*0.77
{68} BZeo_RO2_O6 + HO2 -> BZeo_RO_O5 + OH: KRO2HO2*0.4*0.77
{69} BZeo_RO2_O8 + HO2 -> BZeo_RO_O7 + OH: KRO2HO2*0.98*0.77
{70} BZeo_RO2_O10 + HO2 -> BZeo_RO_O9 + OH: KRO2HO2*0.45*0.77
{71} C5_RO2_O6 + HO2 -> MALDIAL + CO + HO2 KRO2HO2*0.995*0.77
{72} C5_RO2_O7 + HO2 -> MALDIAL + CO + HO2 KRO2HO2*0.4*0.77
{73} C5_RO2_O8 + HO2 -> MALDIAL + CO + HO2 KRO2HO2*0.4*0.77
{74} C5_RO2_O9 + HO2 -> MALDIAL + CO + HO2 KRO2HO2*0.9*0.77
{75} C5_RO2_O10 + HO2 -> MALDIAL + CO + HO2 KRO2HO2*0.4*0.77

Reaction (R5a)

Peroxy radical reacting with pool of RO_2s forming alkoxy radical by O removal:

{76} BZo_RO2_O7 -> BZo_RO_O6 RO2*2.62e-11*0.6*0.4
{77} BZo_RO2_O9 -> BZo_RO_O8 RO2*5.38e-11*0.6*0.4
{78} BZo_RO2_O11 -> BZo_RO_O10 RO2*8.14e-11*0.6*0.4
{79} BZeo_RO2_O6 -> BZeo_RO_O5 RO2*1.24e-11*0.6*0.4
{80} BZeo_RO2_O8 -> BZeo_RO_O7 RO2*4e-11*0.6*0.4
{81} BZeo_RO2_O10 -> BZeo_RO_O9 RO2*6.76e-11*0.6*0.4

Reaction (R5b)

Peroxy radicals forming the dimer ROOR (i.e., accretion product)

RO_2 dimer formation: RO_2 + RO_2" -> ROOR"

ROOR precursor BZo_RO2_O7

{82} BZo_RO2_O7 + BZo_RO2_O7 -> BZoO6_BZoO6 4.17e-11*1.0
{83} BZo_RO2_O7 + BZo_RO2_O9 -> BZoO6_BZoO8 1.e-10*1.0
{84} BZo_RO2_O7 + BZo_RO2_O11 -> BZoO6_BZoO10 9.69e-11*1.0
{85} BZo_RO2_O7 + BZeo_RO2_O6 -> BZoO6_BZeoO5 2.79e-11*1.0
{86} BZo_RO2_O7 + BZeo_RO2_O8 -> BZoO6_BZeoO7 5.55e-11*1.0
{87} BZo_RO2_O7 + BZeo_RO2_O10 -> BZoO6_BZeoO9 8.31e-11*1.0
{88} BZo_RO2_O7 + C5_RO2_O6 -> BZoO6_C5eO5 4.51e-11*1.0
{89} BZo_RO2_O7 + C5_RO2_O7 -> BZoO6_C5O6 5.89e-11*1.0
{90} BZo_RO2_O7 + C5_RO2_O8 -> BZoO6_C5eO7 7.27e-11*1.0
{91} BZo_RO2_O7 + C5_RO2_O9 -> BZoO6_C5O8 8.65e-11*1.0
{92} BZo_RO2_O7 + C5_RO2_O10 -> BZoO6_C5eO9 1e-10*1.0
{93} BZo_RO2_O7 + BZBIPERO2 -> BZoO6_BZBI 1.41e-11*0.15
{94} BZo_RO2_O7 + BZEMUCCO3 -> BZoO6_BZMUa 1.24e-11*1.0
{95} BZo_RO2_O7 + BZEMUCO2 -> BZoO6_BZMUb 2.79e-11*1.0
{96} BZo_RO2_O7 + C5DIALO2 -> BZoO6_C5DI 3e-13*1.0
{97} BZo_RO2_O7 + NPHENO2 -> BZoO6_NPHa 6.67e-11*1.0
{98} BZo_RO2_O7 + PHENO2 -> BZoO6_PHEN 2.79e-11*1.0
{99} BZo_RO2_O7 + MALDIALCO3 -> BZoO6_MALa 3e-13*1.0
{100} BZo_RO2_O7 + EPXDLCO3 -> BZoO6_EPX 3e-13*1.0
{101} BZo_RO2_O7 + C3DIALO2 -> BZoO6_C3DI 3e-13*1.0
{102} BZo_RO2_O7 + MALDIALO2 -> BZoO6_MALb 3e-13*1.0
{103} BZo_RO2_O7 + C6H5O2 -> BZoO6_C6a 3e-13*1.0
{104} BZo_RO2_O7 + NBZFUO2 -> BZoO6_NBZa 3.05e-11*1.0
{105} BZo_RO2_O7 + BZFUO2 -> BZoO6_BZFU 3e-13*1.0
{106} BZo_RO2_O7 + HCOCOHCO3 -> BZoO6_C3a 3e-13*1.0
{107} BZo_RO2_O7 + CATEC1O2 -> BZoO6_CATE 3e-13*1.0
{108} BZo_RO2_O7 + HCOCO3 -> BZoO6_C2a 3e-13*1.0
{109} BZo_RO2_O7 + HCOCH2O2 -> BZoO6_C2b 3e-13*1.0
{110} BZo_RO2_O7 + NPHEN1O2 -> BZoO6_NPHb 9.78e-12*1.0
{111} BZo_RO2_O7 + NNCATECO2 -> BZoO6_NNC 1e-10*1.0
{112} BZo_RO2_O7 + NCATECO2 -> BZoO6_NCAT 8.05e-11*1.0
{113} BZo_RO2_O7 + NBZQO2 -> BZoO6_NBZb 5.12e-11*1.0
{114} BZo_RO2_O7 + PBZQO2 -> BZoO6_PBZ 1.24e-11*1.0
{115} BZo_RO2_O7 + MALANHYO2 -> BZoO6_MALc 3.75e-12*1.0
{116} BZo_RO2_O7 + NDNPHENO2 -> BZoO6_NDN 1e-10*1.0
{117} BZo_RO2_O7 + DNPHENO2 -> BZoO6_DNP 1e-10*1.0
{118} BZo_RO2_O7 + HOCH2CO3 -> BZoO6_C2c 3e-13*1.0
{119} BZo_RO2_O7 + C5CO2OHCO3 -> BZoO6_C5a 2.62e-11*1.0
{120} BZo_RO2_O7 + C4CO2DBCO3 -> BZoO6_C4a 3e-13*1.0
ROOR precursor BZo_RO2_O9
{121} BZo_RO2_O9 + BZo_RO2_O9 -> BZoO8_BZoO8 9.69e-11*1.0
{122} BZo_RO2_O9 + BZo_RO2_O11 -> BZoO8_BZoO10 1e-10*1.0
{123} BZo_RO2_O9 + BZeo_RO2_O6 -> BZoO8_BZeoO5 5.55e-11*1.0
{124} BZo_RO2_O9 + BZeo_RO2_O8 -> BZoO8_BZeoO7 8.31e-11*1.0
{125} BZo_RO2_O9 + BZeo_RO2_O10 -> BZoO8_BZeoO9 1e-10*1.0
{126} BZo_RO2_O9 + C5_RO2_O6 -> BZoO8_C5eO5 7.27e-11*1.0
{127} BZo_RO2_O9 + C5_RO2_O7 -> BZoO8_C5O6 8.65e-11*1.0
{128} BZo_RO2_O9 + C5_RO2_O8 -> BZoO8_C5eO7 1e-10*1.0
{129} BZo_RO2_O9 + C5_RO2_O9 -> BZoO8_C5O8 1e-10*1.0
{130} BZo_RO2_O9 + C5_RO2_O10 -> BZoO8_C5eO9 1e-10*1.0

{131} BZo_RO2_O9 + BZBIPERO2 -> BZoO8_BZBI 4.17e-11*0.5
{132} BZo_RO2_O9 + BZEMUCCO3 -> BZoO8_BZMUa 4e-11*1.0
{133} BZo_RO2_O9 + BZEMUCO2 -> BZoO8_BZMUb 5.55e-11*1.0
{134} BZo_RO2_O9 + C5DIALO2 -> BZoO8_C5DI 1.58e-11*1.0
{135} BZo_RO2_O9 + NPHENO2 -> BZoO8_NPHa 9.43e-11*1.0
{136} BZo_RO2_O9 + PHENO2 -> BZoO8_PHEN 5.55e-11*1.0
{137} BZo_RO2_O9 + MALDIALCO3 -> BZoO8_MALa 3.75e-12*1.0
{138} BZo_RO2_O9 + EPXDLCO3 -> BZoO8_EPX 1.75e-11*1.0
{139} BZo_RO2_O9 + C3DIALO2 -> BZoO8_C3DI 3e-13*1.0
{140} BZo_RO2_O9 + MALDIALO2 -> BZoO8_MALb 1.93e-11*1.0
{141} BZo_RO2_O9 + C6H5O2 -> BZoO8_C6a 3e-13*1.0
{142} BZo_RO2_O9 + NBZFUO2 -> BZoO8_NBZa 5.81e-11*1.0
{143} BZo_RO2_O9 + BZFUO2 -> BZoO8_BZFU 1.93e-11*1.0
{144} BZo_RO2_O9 + HCOCOHCO3 -> BZoO8_C3a 7.2e-12*1.0
{145} BZo_RO2_O9 + CATEC1O2 -> BZoO8_CATE 1.24e-11*1.0
{146} BZo_RO2_O9 + HCOCO3 -> BZoO8_C2a 3e-13*1.0
{147} BZo_RO2_O9 + HCOCH2O2 -> BZoO8_C2b 3e-13*1.0
{148} BZo_RO2_O9 + NPHEN1O2 -> BZoO8_NPHb 3.74e-11*1.0
{149} BZo_RO2_O9 + NNCATECO2 -> BZoO8_NNC 1e-10*1.0
{150} BZo_RO2_O9 + NCATECO2 -> BZoO8_NCAT 1e-10*1.0
{151} BZo_RO2_O9 + NBZQO2 -> BZoO8_NBZb 7.87e-11*1.0
{152} BZo_RO2_O9 + PBZQO2 -> BZoO8_PBZ 4e-11*1.0
{153} BZo_RO2_O9 + MALANHYO2 -> BZoO8_MALc 3.13e-11*1.0
{154} BZo_RO2_O9 + NDNPHENO2 -> BZoO8_NDN 1e-10*1.0
{155} BZo_RO2_O9 + DNPHENO2 -> BZoO8_DNP 1e-10*1.0
{156} BZo_RO2_O9 + HOCH2CO3 -> BZoO8_C2c 3e-13*1.0
{157} BZo_RO2_O9 + C5CO2OHCO3 -> BZoO8_C5a 5.37e-11*1.0
{158} BZo_RO2_O9 + C4CO2DBCO3 -> BZoO8_C4a 2.79e-11*1.0
ROOR precursor BZo_RO2_O11
{159} BZo_RO2_O11 + BZo_RO2_O11 -> BZoO10_BZoO10 1e-10*1.0
{160} BZo_RO2_O11 + BZeo_RO2_O6 -> BZoO10_BZeoO5 8.31e-11*1.0
{161} BZo_RO2_O11 + BZeo_RO2_O8 -> BZoO10_BZeoO7 1e-10*1.0
{162} BZo_RO2_O11 + BZeo_RO2_O10 -> BZoO10_BZeoO9 1e-10*1.0
{163} BZo_RO2_O11 + C5_RO2_O6 -> BZoO10_C5eO5 1e-10*1.0
{164} BZo_RO2_O11 + C5_RO2_O7 -> BZoO10_C5O6 1e-10*1.0
{165} BZo_RO2_O11 + C5_RO2_O8 -> BZoO10_C5eO7 1e-10*1.0
{166} BZo_RO2_O11 + C5_RO2_O9 -> BZoO10_C5O8 1e-10*1.0
{167} BZo_RO2_O11 + C5_RO2_O10 -> BZoO10_C5eO9 1e-10*1.0
{168} BZo_RO2_O11 + BZBIPERO2 -> BZoO10_BZBI 1.e-10*1.0
{169} BZo_RO2_O11 + BZEMUCCO3 -> BZoO10_BZMUa 6.75e-11*1.0
{170} BZo_RO2_O11 + BZEMUCO2 -> BZoO10_BZMUb 8.31e-11*1.0
{171} BZo_RO2_O11 + C5DIALO2 -> BZoO10_C5DI 4.34e-11*1.0
{172} BZo_RO2_O11 + NPHENO2 -> BZoO10_NPHa 1e-10*1.0
{173} BZo_RO2_O11 + PHENO2 -> BZoO10_PHEN 8.31e-11*1.0
{174} BZo_RO2_O11 + MALDIALCO3 -> BZoO10_MALa 3.13e-11*1.0
{175} BZo_RO2_O11 + EPXDLCO3 -> BZoO10_EPX 4.51e-11*1.0
{176} BZo_RO2_O11 + C3DIALO2 -> BZoO10_C3DI 2.1e-11*1.0
{177} BZo_RO2_O11 + MALDIALO2 -> BZoO10_MALb 4.69e-11*1.0
{178} BZo_RO2_O11 + C6H5O2 -> BZoO10_C6a 2.62e-11*1.0
{179} BZo_RO2_O11 + NBZFUO2 -> BZoO10_NBZa 8.56e-11*1.0

{180}	BZo_RO2_O11 + BZFUO2 -> BZoO10_BZFU	4.69e-11*1.0
{181}	BZo_RO2_O11 + HCOCOHCO3 -> BZoO10_C3a	3.48e-11*1.0
{182}	BZo_RO2_O11 + CATEC1O2 -> BZoO10_CATE	4e-11*1.0
{183}	BZo_RO2_O11 + HCOCO3 -> BZoO10_C2a	8.92e-12*1.0
{184}	BZo_RO2_O11 + HCOCH2O2 -> BZoO10_C2b	3e-13*1.0
{185}	BZo_RO2_O11 + NPHEN1O2 -> BZoO10_NPHb	6.5e-11*1.0
{186}	BZo_RO2_O11 + NNCATECO2 -> BZoO10_NNC	1e-10*1.0
{187}	BZo_RO2_O11 + NCATECO2 -> BZoO10_NCAT	1e-10*1.0
{188}	BZo_RO2_O11 + NBZQO2 -> BZoO10_NBZb	1e-10*1.0
{189}	BZo_RO2_O11 + PBZQO2 -> BZoO10_PBZ	6.75e-11*1.0
{190}	BZo_RO2_O11 + MALANHYO2 -> BZoO10_MALc	5.89e-11*1.0
{191}	BZo_RO2_O11 + NDNPHENO2 -> BZoO10_NDN	1e-10*1.0
{192}	BZo_RO2_O11 + DNPHENO2 -> BZoO10_DNP	1e-10*1.0
{193}	BZo_RO2_O11 + HOCH2CO3 -> BZoO10_C2c	1.06e-11*1.0
{194}	BZo_RO2_O11 + C5CO2OHCO3 -> BZoO10_C5a	8.13e-11*1.0
{195}	BZo_RO2_O11 + C4CO2DBCO3 -> BZoO10_C4a	5.55e-11*1.0
ROOR precursor BZeo_RO2_O6
{196}	BZeo_RO2_O6 + BZeo_RO2_O6 -> BZeoO5_BZeoO5	1.41e-11*1.0
{197}	BZeo_RO2_O6 + BZeo_RO2_O8 -> BZeoO5_BZeoO7	4.17e-11*1.0
{198}	BZeo_RO2_O6 + BZeo_RO2_O10 -> BZeoO5_BZeoO9	1.e-10*1.0
{199}	BZeo_RO2_O6 + C5_RO2_O6 -> BZeoO5_C5eO5	3.13e-11*1.0
{200}	BZeo_RO2_O6 + C5_RO2_O7 -> BZeoO5_C5O6	4.51e-11*1.0
{201}	BZeo_RO2_O6 + C5_RO2_O8 -> BZeoO5_C5eO7	5.89e-11*1.0
{202}	BZeo_RO2_O6 + C5_RO2_O9 -> BZeoO5_C5O8	7.27e-11*1.0
{203}	BZeo_RO2_O6 + C5_RO2_O10 -> BZeoO5_C5eO9	8.65e-11*1.0
{204}	BZeo_RO2_O6 + BZBIPERO2 -> BZeoO5_BZBI	3e-13*1.0
{205}	BZeo_RO2_O6 + BZEMUCCO3 -> BZeoO5_BZMUa	3e-13*1.0
{206}	BZeo_RO2_O6 + BZEMUCO2 -> BZeoO5_BZMUb	1.41e-11*1.0
{207}	BZeo_RO2_O6 + C5DIALO2 -> BZeoO5_C5DI	3e-13*1.0
{208}	BZeo_RO2_O6 + NPHENO2 -> BZeoO5_NPHa	5.29e-11*1.0
{209}	BZeo_RO2_O6 + PHENO2 -> BZeoO5_PHEN	1.41e-11*1.0
{210}	BZeo_RO2_O6 + MALDIALCO3 -> BZeoO5_MALa	3e-13*1.0
{211}	BZeo_RO2_O6 + EPXDLCO3 -> BZeoO5_EPX	3e-13*1.0
{212}	BZeo_RO2_O6 + C3DIALO2 -> BZeoO5_C3DI	3e-13*1.0
{213}	BZeo_RO2_O6 + MALDIALO2 -> BZeoO5_MALb	3e-13*1.0
{214}	BZeo_RO2_O6 + C6H5O2 -> BZeoO5_C6a	3e-13*1.0
{215}	BZeo_RO2_O6 + NBZFUO2 -> BZeoO5_NBZa	1.67e-11*1.0
{216}	BZeo_RO2_O6 + BZFUO2 -> BZeoO5_BZFU	3e-13*1.0
{217}	BZeo_RO2_O6 + HCOCOHCO3 -> BZeoO5_C3a	3e-13*1.0
{218}	BZeo_RO2_O6 + CATEC1O2 -> BZeoO5_CATE	3e-13*1.0
{219}	BZeo_RO2_O6 + HCOCO3 -> BZeoO5_C2a	3e-13*1.0
{220}	BZeo_RO2_O6 + HCOCH2O2 -> BZeoO5_C2b	3e-13*1.0
{221}	BZeo_RO2_O6 + NPHEN1O2 -> BZeoO5_NPHb	3e-13*1.0
{222}	BZeo_RO2_O6 + NNCATECO2 -> BZeoO5_NNC	1e-10*1.0
{223}	BZeo_RO2_O6 + NCATECO2 -> BZeoO5_NCAT	6.67e-11*1.0
{224}	BZeo_RO2_O6 + NBZQO2 -> BZeoO5_NBZb	3.74e-11*1.0
{225}	BZeo_RO2_O6 + PBZQO2 -> BZeoO5_PBZ	3e-13*1.0
{226}	BZeo_RO2_O6 + MALANHYO2 -> BZeoO5_MALc	3e-13*1.0
{227}	BZeo_RO2_O6 + NDNPHENO2 -> BZeoO5_NDN	1e-10*1.0
{228}	BZeo_RO2_O6 + DNPHENO2 -> BZeoO5_DNP	9.17e-11*1.0

{229} BZeo_RO2_O6 + HOCH2CO3 -> BZeoO5_C2c 3e-13*1.0
{230} BZeo_RO2_O6 + C5CO2OHCO3 -> BZeoO5_C5a 1.24e-11*1.0
{231} BZeo_RO2_O6 + C4CO2DBCO3 -> BZeoO5_C4a 3e-13*1.0
ROOR precursor BZeo_RO2_O8
{232} BZeo_RO2_O8 + BZeo_RO2_O8 -> BZeoO7_BZeoO7 1.e-10*1.0
{233} BZeo_RO2_O8 + BZeo_RO2_O10 -> BZeoO7_BZeoO9 9.69e-11*1.0
{234} BZeo_RO2_O8 + C5_RO2_O6 -> BZeoO7_C5eO5 5.89e-11*1.0
{235} BZeo_RO2_O8 + C5_RO2_O7 -> BZeoO7_C5O6 7.27e-11*1.0
{236} BZeo_RO2_O8 + C5_RO2_O8 -> BZeoO7_C5eO7 8.65e-11*1.0
{237} BZeo_RO2_O8 + C5_RO2_O9 -> BZeoO7_C5O8 1e-10*1.0
{238} BZeo_RO2_O8 + C5_RO2_O10 -> BZeoO7_C5eO9 1e-10*1.0
{239} BZeo_RO2_O8 + BZBIPERO2 -> BZeoO7_BZBI 2.79e-11*0.3
{240} BZeo_RO2_O8 + BZEMUCCO3 -> BZeoO7_BZMUa 2.62e-11*1.0
{241} BZeo_RO2_O8 + BZEMUCO2 -> BZeoO7_BZMUb 4.17e-11*1.0
{242} BZeo_RO2_O8 + C5DIALO2 -> BZeoO7_C5DI 2.02e-12*1.0
{243} BZeo_RO2_O8 + NPHENO2 -> BZeoO7_NPHa 8.05e-11*1.0
{244} BZeo_RO2_O8 + PHENO2 -> BZeoO7_PHEN 4.17e-11*1.0
{245} BZeo_RO2_O8 + MALDIALCO3 -> BZeoO7_MALa 3e-13*1.0
{246} BZeo_RO2_O8 + EPXDLCO3 -> BZeoO7_EPX 3.75e-12*1.0
{247} BZeo_RO2_O8 + C3DIALO2 -> BZeoO7_C3DI 3e-13*1.0
{248} BZeo_RO2_O8 + MALDIALO2 -> BZeoO7_MALb 5.47e-12*1.0
{249} BZeo_RO2_O8 + C6H5O2 -> BZeoO7_C6a 3e-13*1.0
{250} BZeo_RO2_O8 + NBZFUO2 -> BZeoO7_NBZa 4.43e-11*1.0
{251} BZeo_RO2_O8 + BZFUO2 -> BZeoO7_BZFU 5.47e-12*1.0
{252} BZeo_RO2_O8 + HCOCOHCO3 -> BZeoO7_C3a 3e-13*1.0
{253} BZeo_RO2_O8 + CATEC1O2 -> BZeoO7_CATE 3e-13*1.0
{254} BZeo_RO2_O8 + HCOCO3 -> BZeoO7_C2a 3e-13*1.0
{255} BZeo_RO2_O8 + HCOCH2O2 -> BZeoO7_C2b 3e-13*1.0
{256} BZeo_RO2_O8 + NPHEN1O2 -> BZeoO7_NPHb 2.36e-11*1.0
{257} BZeo_RO2_O8 + NNCATECO2 -> BZeoO7_NNC 1e-10*1.0
{258} BZeo_RO2_O8 + NCATECO2 -> BZeoO7_NCAT 9.43e-11*1.0
{259} BZeo_RO2_O8 + NBZQO2 -> BZeoO7_NBZb 6.5e-11*1.0
{260} BZeo_RO2_O8 + PBZQO2 -> BZeoO7_PBZ 2.62e-11*1.0
{261} BZeo_RO2_O8 + MALANHYO2 -> BZeoO7_MALc 1.75e-11*1.0
{262} BZeo_RO2_O8 + NDNPHENO2 -> BZeoO7_NDN 1e-10*1.0
{263} BZeo_RO2_O8 + DNPHENO2 -> BZeoO7_DNP 1e-10*1.0
{264} BZeo_RO2_O8 + HOCH2CO3 -> BZeoO7_C2c 3e-13*1.0
{265} BZeo_RO2_O8 + C5CO2OHCO3 -> BZeoO7_C5a 4e-11*1.0
{266} BZeo_RO2_O8 + C4CO2DBCO3 -> BZeoO7_C4a 1.41e-11*1.0
ROOR precursor BZeo_RO2_O10
{267} BZeo_RO2_O10 + BZeo_RO2_O10 -> BZeoO9_BZeoO9 1e-10*1.0
{268} BZeo_RO2_O10 + C5_RO2_O6 -> BZeoO9_C5eO5 8.65e-11*1.0
{269} BZeo_RO2_O10 + C5_RO2_O7 -> BZeoO9_C5O6 1e-10*1.0
{270} BZeo_RO2_O10 + C5_RO2_O8 -> BZeoO9_C5eO7 1e-10*1.0
{271} BZeo_RO2_O10 + C5_RO2_O9 -> BZeoO9_C5O8 1e-10*1.0
{272} BZeo_RO2_O10 + C5_RO2_O10 -> BZeoO9_C5eO9 1e-10*1.0
{273} BZeo_RO2_O10 + BZBIPERO2 -> BZeoO9_BZBI 5.55e-11*1.0
{274} BZeo_RO2_O10 + BZEMUCCO3 -> BZeoO9_BZMUa 5.37e-11*1.0
{275} BZeo_RO2_O10 + BZEMUCO2 -> BZeoO9_BZMUb 1.e-10*1.0
{276} BZeo_RO2_O10 + C5DIALO2 -> BZeoO9_C5DI 2.96e-11*1.0

{277} BZeo_RO2_O10 + NPHENO2 -> BZeoO9_NPHa 1e-10*1.0
{278} BZeo_RO2_O10 + PHENO2 -> BZeoO9_PHEN 1.e-10*1.0
{279} BZeo_RO2_O10 + MALDIALCO3 -> BZeoO9_MALa 1.75e-11*1.0
{280} BZeo_RO2_O10 + EPXDLCO3 -> BZeoO9_EPX 3.13e-11*1.0
{281} BZeo_RO2_O10 + C3DIALO2 -> BZeoO9_C3DI 7.2e-12*1.0
{282} BZeo_RO2_O10 + MALDIALO2 -> BZeoO9_MALb 3.31e-11*1.0
{283} BZeo_RO2_O10 + C6H5O2 -> BZeoO9_C6a 1.24e-11*1.0
{284} BZeo_RO2_O10 + NBZFUO2 -> BZeoO9_NBZa 7.19e-11*1.0
{285} BZeo_RO2_O10 + BZFUO2 -> BZeoO9_BZFU 3.31e-11*1.0
{286} BZeo_RO2_O10 + HCOCOHCO3 -> BZeoO9_C3a 2.1e-11*1.0
{287} BZeo_RO2_O10 + CATEC1O2 -> BZeoO9_CATE 2.62e-11*1.0
{288} BZeo_RO2_O10 + HCOCO3 -> BZeoO9_C2a 3e-13*1.0
{289} BZeo_RO2_O10 + HCOCH2O2 -> BZeoO9_C2b 3e-13*1.0
{290} BZeo_RO2_O10 + NPHEN1O2 -> BZeoO9_NPHb 5.12e-11*1.0
{291} BZeo_RO2_O10 + NNCATECO2 -> BZeoO9_NNC 1e-10*1.0
{292} BZeo_RO2_O10 + NCATECO2 -> BZeoO9_NCAT 1e-10*1.0
{293} BZeo_RO2_O10 + NBZQO2 -> BZeoO9_NBZb 9.25e-11*1.0
{294} BZeo_RO2_O10 + PBZQO2 -> BZeoO9_PBZ 5.37e-11*1.0
{295} BZeo_RO2_O10 + MALANHYO2 -> BZeoO9_MALc 4.51e-11*1.0
{296} BZeo_RO2_O10 + NDNPHENO2 -> BZeoO9_NDN 1e-10*1.0
{297} BZeo_RO2_O10 + DNPHENO2 -> BZeoO9_DNP 1e-10*1.0
{298} BZeo_RO2_O10 + HOCH2CO3 -> BZeoO9_C2c 3e-13*1.0
{299} BZeo_RO2_O10 + C5CO2OHCO3 -> BZeoO9_C5a 6.75e-11*1.0
{300} BZeo_RO2_O10 + C4CO2DBCO3 -> BZeoO9_C4a 4.17e-11*1.0
ROOR precursor C5_RO2_O6
{301} C5_RO2_O6 + C5_RO2_O6 -> C5eO5_C5eO5 4.86e-11*1.0
{302} C5_RO2_O6 + C5_RO2_O7 -> C5eO5_C5O6 6.24e-11*1.0
{303} C5_RO2_O6 + C5_RO2_O8 -> C5eO5_C5eO7 7.62e-11*1.0
{304} C5_RO2_O6 + C5_RO2_O9 -> C5eO5_C5O8 9e-11*1.0
{305} C5_RO2_O6 + C5_RO2_O10 -> C5eO5_C5eO9 1e-10*1.0
{306} C5_RO2_O6 + BZBIPERO2 -> C5eO5_BZBI 1.75e-11*1.0
{307} C5_RO2_O6 + BZEMUCCO3 -> C5eO5_BZMUa 1.58e-11*1.0
{308} C5_RO2_O6 + BZEMUCO2 -> C5eO5_BZMUb 3.13e-11*1.0
{309} C5_RO2_O6 + C5DIALO2 -> C5eO5_C5DI 3e-13*1.0
{310} C5_RO2_O6 + NPHENO2 -> C5eO5_NPHa 7.01e-11*1.0
{311} C5_RO2_O6 + PHENO2 -> C5eO5_PHEN 3.13e-11*1.0
{312} C5_RO2_O6 + MALDIALCO3 -> C5eO5_MALa 3e-13*1.0
{313} C5_RO2_O6 + EPXDLCO3 -> C5eO5_EPX 3e-13*1.0
{314} C5_RO2_O6 + C3DIALO2 -> C5eO5_C3DI 3e-13*1.0
{315} C5_RO2_O6 + MALDIALO2 -> C5eO5_MALb 3e-13*1.0
{316} C5_RO2_O6 + C6H5O2 -> C5eO5_C6a 3e-13*1.0
{317} C5_RO2_O6 + NBZFUO2 -> C5eO5_NBZa 3.39e-11*1.0
{318} C5_RO2_O6 + BZFUO2 -> C5eO5_BZFU 3e-13*1.0
{319} C5_RO2_O6 + HCOCOHCO3 -> C5eO5_C3a 3e-13*1.0
{320} C5_RO2_O6 + CATEC1O2 -> C5eO5_CATE 3e-13*1.0
{321} C5_RO2_O6 + HCOCO3 -> C5eO5_C2a 3e-13*1.0
{322} C5_RO2_O6 + HCOCH2O2 -> C5eO5_C2b 3e-13*1.0
{323} C5_RO2_O6 + NPHEN1O2 -> C5eO5_NPHb 1.32e-11*1.0
{324} C5_RO2_O6 + NNCATECO2 -> C5eO5_NNC 1e-10*1.0
{325} C5_RO2_O6 + NCATECO2 -> C5eO5_NCAT 8.39e-11*1.0

{326} C5_RO2_O6 + NBZQO2 -> C5eO5_NBZb 5.46e-11*1.0
{327} C5_RO2_O6 + PBZQO2 -> C5eO5_PBZ 1.58e-11*1.0
{328} C5_RO2_O6 + MALANHYO2 -> C5eO5_MALc 7.2e-12*1.0
{329} C5_RO2_O6 + NDNPHENO2 -> C5eO5_NDN 1e-10*1.0
{330} C5_RO2_O6 + DNPHENO2 -> C5eO5_DNP 1e-10*1.0
{331} C5_RO2_O6 + HOCH2CO3 -> C5eO5_C2c 3e-13*1.0
{332} C5_RO2_O6 + C5CO2OHCO3 -> C5eO5_C5a 2.96e-11*1.0
{333} C5_RO2_O6 + C4CO2DBCO3 -> C5eO5_C4a 3.75e-12*1.0
ROOR precursor C5_RO2_O7
{334} C5_RO2_O7 + C5_RO2_O7 -> C5O6_C5O6 7.62e-11*1.0
{335} C5_RO2_O7 + C5_RO2_O8 -> C5O6_C5eO7 9e-11*1.0
{336} C5_RO2_O7 + C5_RO2_O9 -> C5O6_C5O8 1e-10*1.0
{337} C5_RO2_O7 + C5_RO2_O10 -> C5O6_C5eO9 1e-10*1.0
{338} C5_RO2_O7 + BZBIPERO2 -> C5O6_BZBI 3.13e-11*1.0
{339} C5_RO2_O7 + BZEMUCCO3 -> C5O6_BZMUa 2.96e-11*1.0
{340} C5_RO2_O7 + BZEMUCO2 -> C5O6_BZMUb 4.51e-11*1.0
{341} C5_RO2_O7 + C5DIALO2 -> C5O6_C5DI 5.47e-12*1.0
{342} C5_RO2_O7 + NPHENO2 -> C5O6_NPHa 8.39e-11*1.0
{343} C5_RO2_O7 + PHENO2 -> C5O6_PHEN 4.51e-11*1.0
{344} C5_RO2_O7 + MALDIALCO3 -> C5O6_MALa 3e-13*1.0
{345} C5_RO2_O7 + EPXDLCO3 -> C5O6_EPX 7.2e-12*1.0
{346} C5_RO2_O7 + C3DIALO2 -> C5O6_C3DI 3e-13*1.0
{347} C5_RO2_O7 + MALDIALO2 -> C5O6_MALb 8.92e-12*1.0
{348} C5_RO2_O7 + C6H5O2 -> C5O6_C6a 3e-13*1.0
{349} C5_RO2_O7 + NBZFUO2 -> C5O6_NBZa 4.77e-11*1.0
{350} C5_RO2_O7 + BZFUO2 -> C5O6_BZFU 8.92e-12*1.0
{351} C5_RO2_O7 + HCOCOHCO3 -> C5O6_C3a 3e-13*1.0
{352} C5_RO2_O7 + CATEC1O2 -> C5O6_CATE 2.02e-12*1.0
{353} C5_RO2_O7 + HCOCO3 -> C5O6_C2a 3e-13*1.0
{354} C5_RO2_O7 + HCOCH2O2 -> C5O6_C2b 3e-13*1.0
{355} C5_RO2_O7 + NPHEN1O2 -> C5O6_NPHb 2.7e-11*1.0
{356} C5_RO2_O7 + NNCATECO2 -> C5O6_NNC 1e-10*1.0
{357} C5_RO2_O7 + NCATECO2 -> C5O6_NCAT 9.77e-11*1.0
{358} C5_RO2_O7 + NBZQO2 -> C5O6_NBZb 6.84e-11*1.0
{359} C5_RO2_O7 + PBZQO2 -> C5O6_PBZ 2.96e-11*1.0
{360} C5_RO2_O7 + MALANHYO2 -> C5O6_MALc 2.1e-11*1.0
{361} C5_RO2_O7 + NDNPHENO2 -> C5O6_NDN 1e-10*1.0
{362} C5_RO2_O7 + DNPHENO2 -> C5O6_DNP 1e-10*1.0
{363} C5_RO2_O7 + HOCH2CO3 -> C5O6_C2c 3e-13*1.0
{364} C5_RO2_O7 + C5CO2OHCO3 -> C5O6_C5a 4.34e-11*1.0
{365} C5_RO2_O7 + C4CO2DBCO3 -> C5O6_C4a 1.75e-11*1.0
ROOR precursor C5_RO2_O8
{366} C5_RO2_O8 + C5_RO2_O8 -> C5eO7_C5eO7 1e-10*1.0
{367} C5_RO2_O8 + C5_RO2_O9 -> C5eO7_C5O8 1e-10*1.0
{368} C5_RO2_O8 + C5_RO2_O10 -> C5eO7_C5eO9 1e-10*1.0
{369} C5_RO2_O8 + BZBIPERO2 -> C5eO7_BZBI 4.51e-11*0.3
{370} C5_RO2_O8 + BZEMUCCO3 -> C5eO7_BZMUa 4.34e-11*1.0
{371} C5_RO2_O8 + BZEMUCO2 -> C5eO7_BZMUb 5.89e-11*1.0
{372} C5_RO2_O8 + C5DIALO2 -> C5eO7_C5DI 1.93e-11*1.0
{373} C5_RO2_O8 + NPHENO2 -> C5eO7_NPHa 9.77e-11*1.0

{374} C5_RO2_O8 + PHENO2 -> C5eO7_PHEN 5.89e-11*1.0
{375} C5_RO2_O8 + MALDIALCO3 -> C5eO7_MALa 7.2e-12*1.0
{376} C5_RO2_O8 + EPXDLCO3 -> C5eO7_EPX 2.1e-11*1.0
{377} C5_RO2_O8 + C3DIALO2 -> C5eO7_C3DI 3e-13*1.0
{378} C5_RO2_O8 + MALDIALO2 -> C5eO7_MALb 2.27e-11*1.0
{379} C5_RO2_O8 + C6H5O2 -> C5eO7_C6a 2.02e-12*1.0
{380} C5_RO2_O8 + NBZFUO2 -> C5eO7_NBZa 6.15e-11*1.0
{381} C5_RO2_O8 + BZFUO2 -> C5eO7_BZFU 2.27e-11*1.0
{382} C5_RO2_O8 + HCOCOHCO3 -> C5eO7_C3a 1.06e-11*1.0
{383} C5_RO2_O8 + CATEC1O2 -> C5eO7_CATE 1.58e-11*1.0
{384} C5_RO2_O8 + HCOCO3 -> C5eO7_C2a 3e-13*1.0
{385} C5_RO2_O8 + HCOCH2O2 -> C5eO7_C2b 3e-13*1.0
{386} C5_RO2_O8 + NPHEN1O2 -> C5eO7_NPHb 4.08e-11*1.0
{387} C5_RO2_O8 + NNCATECO2 -> C5eO7_NNC 1e-10*1.0
{388} C5_RO2_O8 + NCATECO2 -> C5eO7_NCAT 1e-10*1.0
{389} C5_RO2_O8 + NBZQO2 -> C5eO7_NBZb 8.22e-11*1.0
{390} C5_RO2_O8 + PBZQO2 -> C5eO7_PBZ 4.34e-11*1.0
{391} C5_RO2_O8 + MALANHYO2 -> C5eO7_MALc 3.48e-11*1.0
{392} C5_RO2_O8 + NDNPHENO2 -> C5eO7_NDN 1e-10*1.0
{393} C5_RO2_O8 + DNPHENO2 -> C5eO7_DNP 1e-10*1.0
{394} C5_RO2_O8 + HOCH2CO3 -> C5eO7_C2c 3e-13*1.0
{395} C5_RO2_O8 + C5CO2OHCO3 -> C5eO7_C5a 5.72e-11*1.0
{396} C5_RO2_O8 + C4CO2DBCO3 -> C5eO7_C4a 3.13e-11*1.0
ROOR precursor C5_RO2_O9
{397} C5_RO2_O9 + C5_RO2_O9 -> C5O8_C5O8 1e-10*1.0
{398} C5_RO2_O9 + C5_RO2_O10 -> C5O8_C5eO9 1e-10*1.0
{399} C5_RO2_O9 + BZBIPERO2 -> C5O8_BZBI 5.89e-11*1.0
{400} C5_RO2_O9 + BZEMUCCO3 -> C5O8_BZMUa 5.72e-11*1.0
{401} C5_RO2_O9 + BZEMUCO2 -> C5O8_BZMUb 7.27e-11*1.0
{402} C5_RO2_O9 + C5DIALO2 -> C5O8_C5DI 3.31e-11*1.0
{403} C5_RO2_O9 + NPHENO2 -> C5O8_NPHa 1e-10*1.0
{404} C5_RO2_O9 + PHENO2 -> C5O8_PHEN 7.27e-11*1.0
{405} C5_RO2_O9 + MALDIALCO3 -> C5O8_MALa 2.1e-11*1.0
{406} C5_RO2_O9 + EPXDLCO3 -> C5O8_EPX 3.48e-11*1.0
{407} C5_RO2_O9 + C3DIALO2 -> C5O8_C3DI 1.06e-11*1.0
{408} C5_RO2_O9 + MALDIALO2 -> C5O8_MALb 3.65e-11*1.0
{409} C5_RO2_O9 + C6H5O2 -> C5O8_C6a 1.58e-11*1.0
{410} C5_RO2_O9 + NBZFUO2 -> C5O8_NBZa 7.53e-11*1.0
{411} C5_RO2_O9 + BZFUO2 -> C5O8_BZFU 3.65e-11*1.0
{412} C5_RO2_O9 + HCOCOHCO3 -> C5O8_C3a 2.44e-11*1.0
{413} C5_RO2_O9 + CATEC1O2 -> C5O8_CATE 2.96e-11*1.0
{414} C5_RO2_O9 + HCOCO3 -> C5O8_C2a 3e-13*1.0
{415} C5_RO2_O9 + HCOCH2O2 -> C5O8_C2b 3e-13*1.0
{416} C5_RO2_O9 + NPHEN1O2 -> C5O8_NPHb 5.46e-11*1.0
{417} C5_RO2_O9 + NNCATECO2 -> C5O8_NNC 1e-10*1.0
{418} C5_RO2_O9 + NCATECO2 -> C5O8_NCAT 1e-10*1.0
{419} C5_RO2_O9 + NBZQO2 -> C5O8_NBZb 9.6e-11*1.0
{420} C5_RO2_O9 + PBZQO2 -> C5O8_PBZ 5.72e-11*1.0
{421} C5_RO2_O9 + MALANHYO2 -> C5O8_MALc 4.86e-11*1.0
{422} C5_RO2_O9 + NDNPHENO2 -> C5O8_NDN 1e-10*1.0

{423} C5_RO2_O9 + DNPHENO2 -> C5O8_DNP 1e-10*1.0
{424} C5_RO2_O9 + HOCH2CO3 -> C5O8_C2c 3e-13*1.0
{425} C5_RO2_O9 + C5CO2OHCO3 -> C5O8_C5a 7.1e-11*1.0
{426} C5_RO2_O9 + C4CO2DBCO3 -> C5O8_C4a 4.51e-11*1.0
ROOR precursor C5_RO2_O10
{427} C5_RO2_O10 + C5_RO2_O10 -> C5eO9_C5eO9 1e-10*1.0
{428} C5_RO2_O10 + BZBIPERO2 -> C5eO9_BZBI 7.27e-11*1.0
{429} C5_RO2_O10 + BZEMUCCO3 -> C5eO9_BZMUa 7.1e-11*1.0
{430} C5_RO2_O10 + BZEMUCO2 -> C5eO9_BZMUb 8.65e-11*1.0
{431} C5_RO2_O10 + C5DIALO2 -> C5eO9_C5DI 4.69e-11*1.0
{432} C5_RO2_O10 + NPHENO2 -> C5eO9_NPHa 1e-10*1.0
{433} C5_RO2_O10 + PHENO2 -> C5eO9_PHEN 8.65e-11*1.0
{434} C5_RO2_O10 + MALDIALCO3 -> C5eO9_MALa 3.48e-11*1.0
{435} C5_RO2_O10 + EPXDLCO3 -> C5eO9_EPX 4.86e-11*1.0
{436} C5_RO2_O10 + C3DIALO2 -> C5eO9_C3DI 2.44e-11*1.0
{437} C5_RO2_O10 + MALDIALO2 -> C5eO9_MALb 5.03e-11*1.0
{438} C5_RO2_O10 + C6H5O2 -> C5eO9_C6a 2.96e-11*1.0
{439} C5_RO2_O10 + NBZFUO2 -> C5eO9_NBZa 8.91e-11*1.0
{440} C5_RO2_O10 + BZFUO2 -> C5eO9_BZFU 5.03e-11*1.0
{441} C5_RO2_O10 + HCOCOHCO3 -> C5eO9_C3a 3.82e-11*1.0
{442} C5_RO2_O10 + CATEC1O2 -> C5eO9_CATE 4.34e-11*1.0
{443} C5_RO2_O10 + HCOCO3 -> C5eO9_C2a 1.24e-11*1.0
{444} C5_RO2_O10 + HCOCH2O2 -> C5eO9_C2b 3e-13*1.0
{445} C5_RO2_O10 + NPHEN1O2 -> C5eO9_NPHb 6.84e-11*1.0
{446} C5_RO2_O10 + NNCATECO2 -> C5eO9_NNC 1e-10*1.0
{447} C5_RO2_O10 + NCATECO2 -> C5eO9_NCAT 1e-10*1.0
{448} C5_RO2_O10 + NBZQO2 -> C5eO9_NBZb 1e-10*1.0
{449} C5_RO2_O10 + PBZQO2 -> C5eO9_PBZ 7.1e-11*1.0
{450} C5_RO2_O10 + MALANHYO2 -> C5eO9_MALc 6.24e-11*1.0
{451} C5_RO2_O10 + NDNPHENO2 -> C5eO9_NDN 1e-10*1.0
{452} C5_RO2_O10 + DNPHENO2 -> C5eO9_DNP 1e-10*1.0
{453} C5_RO2_O10 + HOCH2CO3 -> C5eO9_C2c 1.41e-11*1.0
{454} C5_RO2_O10 + C5CO2OHCO3 -> C5eO9_C5a 8.48e-11*1.0
{455} C5_RO2_O10 + C4CO2DBCO3 -> C5eO9_C4a 5.89e-11*1.0
ROOR precursor BZBIPERO2
{456} BZBIPERO2 + BZBIPERO2 -> BZBI_BZBI 3e-13*0.7
{457} BZBIPERO2 + BZEMUCCO3 -> BZBI_BZMUa 3e-13*1.0
{458} BZBIPERO2 + BZEMUCO2 -> BZBI_BZMUb 3e-13*1.0
{459} BZBIPERO2 + C5DIALO2 -> BZBI_C5DI 3e-13*1.0
{460} BZBIPERO2 + NPHENO2 -> BZBI_NPHa 3.91e-11*1.0
{461} BZBIPERO2 + PHENO2 -> BZBI_PHEN 3e-13*1.0
{462} BZBIPERO2 + MALDIALCO3 -> BZBI_MALa 3e-13*1.0
{463} BZBIPERO2 + EPXDLCO3 -> BZBI_EPX 3e-13*1.0
{464} BZBIPERO2 + C3DIALO2 -> BZBI_C3DI 3e-13*1.0
{465} BZBIPERO2 + MALDIALO2 -> BZBI_MALb 3e-13*1.0
{466} BZBIPERO2 + C6H5O2 -> BZBI_C6a 3e-13*1.0
{467} BZBIPERO2 + NBZFUO2 -> BZBI_NBZa 2.89e-12*1.0
{468} BZBIPERO2 + BZFUO2 -> BZBI_BZFU 3e-13*1.0
{469} BZBIPERO2 + HCOCOHCO3 -> BZBI_C3a 3e-13*1.0
{470} BZBIPERO2 + CATEC1O2 -> BZBI_CATE 3e-13*1.0

{471} BZBIPERO2 + HCOCO3 -> BZBI_C2a 3e-13*1.0
{472} BZBIPERO2 + HCOCH2O2 -> BZBI_C2b 3e-13*1.0
{473} BZBIPERO2 + NPHEN1O2 -> BZBI_NPHb 3e-13*1.0
{474} BZBIPERO2 + NNCATECO2 -> BZBI_NNC 9.17e-11*1.0
{475} BZBIPERO2 + NCATECO2 -> BZBI_NCAT 5.29e-11*1.0
{476} BZBIPERO2 + NBZQO2 -> BZBI_NBZb 2.36e-11*1.0
{477} BZBIPERO2 + PBZQO2 -> BZBI_PBZ 3e-13*1.0
{478} BZBIPERO2 + MALANHYO2 -> BZBI_MALc 3e-13*1.0
{479} BZBIPERO2 + NDNPHENO2 -> BZBI_NDN 1e-10*1.0
{480} BZBIPERO2 + DNPHENO2 -> BZBI_DNP 7.79e-11*1.0
{481} BZBIPERO2 + HOCH2CO3 -> BZBI_C2c 3e-13*1.0
{482} BZBIPERO2 + C5CO2OHCO3 -> BZBI_C5a 3e-13*1.0
{483} BZBIPERO2 + C4CO2DBCO3 -> BZBI_C4a 3e-13*1.0
ROOR precursor BZEMUCCO3
{484} BZEMUCCO3 + BZEMUCCO3 -> BZMUa_BZMUa 3e-13*1.0
{485} BZEMUCCO3 + BZEMUCO2 -> BZMUa_BZMUb 3e-13*1.0
{486} BZEMUCCO3 + C5DIALO2 -> BZMUa_C5DI 3e-13*1.0
{487} BZEMUCCO3 + NPHENO2 -> BZMUa_NPHa 3.74e-11*1.0
{488} BZEMUCCO3 + PHENO2 -> BZMUa_PHEN 3e-13*1.0
{489} BZEMUCCO3 + MALDIALCO3 -> BZMUa_MALa 3e-13*1.0
{490} BZEMUCCO3 + EPXDLCO3 -> BZMUa_EPX 3e-13*1.0
{491} BZEMUCCO3 + C3DIALO2 -> BZMUa_C3DI 3e-13*1.0
{492} BZEMUCCO3 + MALDIALO2 -> BZMUa_MALb 3e-13*1.0
{493} BZEMUCCO3 + C6H5O2 -> BZMUa_C6a 3e-13*1.0
{494} BZEMUCCO3 + NBZFUO2 -> BZMUa_NBZa 1.16e-12*1.0
{495} BZEMUCCO3 + BZFUO2 -> BZMUa_BZFU 3e-13*1.0
{496} BZEMUCCO3 + HCOCOHCO3 -> BZMUa_C3a 3e-13*1.0
{497} BZEMUCCO3 + CATEC1O2 -> BZMUa_CATE 3e-13*1.0
{498} BZEMUCCO3 + HCOCO3 -> BZMUa_C2a 3e-13*1.0
{499} BZEMUCCO3 + HCOCH2O2 -> BZMUa_C2b 3e-13*1.0
{500} BZEMUCCO3 + NPHEN1O2 -> BZMUa_NPHb 3e-13*1.0
{501} BZEMUCCO3 + NNCATECO2 -> BZMUa_NNC 9e-11*1.0
{502} BZEMUCCO3 + NCATECO2 -> BZMUa_NCAT 5.12e-11*1.0
{503} BZEMUCCO3 + NBZQO2 -> BZMUa_NBZb 2.19e-11*1.0
{504} BZEMUCCO3 + PBZQO2 -> BZMUa_PBZ 3e-13*1.0
{505} BZEMUCCO3 + MALANHYO2 -> BZMUa_MALc 3e-13*1.0
{506} BZEMUCCO3 + NDNPHENO2 -> BZMUa_NDN 1e-10*1.0
{507} BZEMUCCO3 + DNPHENO2 -> BZMUa_DNP 7.62e-11*1.0
{508} BZEMUCCO3 + HOCH2CO3 -> BZMUa_C2c 3e-13*1.0
{509} BZEMUCCO3 + C5CO2OHCO3 -> BZMUa_C5a 3e-13*1.0
{510} BZEMUCCO3 + C4CO2DBCO3 -> BZMUa_C4a 3e-13*1.0
ROOR precursor BZEMUCO2
{511} BZEMUCO2 + BZEMUCO2 -> BZMUb_BZMUb 1.41e-11*1.0
{512} BZEMUCO2 + C5DIALO2 -> BZMUb_C5DI 3e-13*1.0
{513} BZEMUCO2 + NPHENO2 -> BZMUb_NPHa 5.29e-11*1.0
{514} BZEMUCO2 + PHENO2 -> BZMUb_PHEN 1.41e-11*1.0
{515} BZEMUCO2 + MALDIALCO3 -> BZMUb_MALa 3e-13*1.0
{516} BZEMUCO2 + EPXDLCO3 -> BZMUb_EPX 3e-13*1.0
{517} BZEMUCO2 + C3DIALO2 -> BZMUb_C3DI 3e-13*1.0
{518} BZEMUCO2 + MALDIALO2 -> BZMUb_MALb 3e-13*1.0

{519} BZEMUCO2 + C6H5O2 -> BZMUb_C6a 3e-13*1.0
{520} BZEMUCO2 + NBZFUO2 -> BZMUb_NBZa 1.67e-11*1.0
{521} BZEMUCO2 + BZFUO2 -> BZMUb_BZFU 3e-13*1.0
{522} BZEMUCO2 + HCOCOHCO3 -> BZMUb_C3a 3e-13*1.0
{523} BZEMUCO2 + CATEC1O2 -> BZMUb_CATE 3e-13*1.0
{524} BZEMUCO2 + HCOCO3 -> BZMUb_C2a 3e-13*1.0
{525} BZEMUCO2 + HCOCH2O2 -> BZMUb_C2b 3e-13*1.0
{526} BZEMUCO2 + NPHEN1O2 -> BZMUb_NPHb 3e-13*1.0
{527} BZEMUCO2 + NNCATECO2 -> BZMUb_NNC 1e-10*1.0
{528} BZEMUCO2 + NCATECO2 -> BZMUb_NCAT 6.67e-11*1.0
{529} BZEMUCO2 + NBZQO2 -> BZMUb_NBZb 3.74e-11*1.0
{530} BZEMUCO2 + PBZQO2 -> BZMUb_PBZ 3e-13*1.0
{531} BZEMUCO2 + MALANHYO2 -> BZMUb_MALc 3e-13*1.0
{532} BZEMUCO2 + NDNPHENO2 -> BZMUb_NDN 1e-10*1.0
{533} BZEMUCO2 + DNPHENO2 -> BZMUb_DNP 9.17e-11*1.0
{534} BZEMUCO2 + HOCH2CO3 -> BZMUb_C2c 3e-13*1.0
{535} BZEMUCO2 + C5CO2OHCO3 -> BZMUb_C5a 1.24e-11*1.0
{536} BZEMUCO2 + C4CO2DBCO3 -> BZMUb_C4a 3e-13*1.0
ROOR precursor C5DIALO2
{537} C5DIALO2 + C5DIALO2 -> C5DI_C5DI 3e-13*1.0
{538} C5DIALO2 + NPHENO2 -> C5DI_NPHa 1.32e-11*1.0
{539} C5DIALO2 + PHENO2 -> C5DI_PHEN 3e-13*1.0
{540} C5DIALO2 + MALDIALCO3 -> C5DI_MALa 3e-13*1.0
{541} C5DIALO2 + EPXDLCO3 -> C5DI_EPX 3e-13*1.0
{542} C5DIALO2 + C3DIALO2 -> C5DI_C3DI 3e-13*1.0
{543} C5DIALO2 + MALDIALO2 -> C5DI_MALb 3e-13*1.0
{544} C5DIALO2 + C6H5O2 -> C5DI_C6a 3e-13*1.0
{545} C5DIALO2 + NBZFUO2 -> C5DI_NBZa 3e-13*1.0
{546} C5DIALO2 + BZFUO2 -> C5DI_BZFU 3e-13*1.0
{547} C5DIALO2 + HCOCOHCO3 -> C5DI_C3a 3e-13*1.0
{548} C5DIALO2 + CATEC1O2 -> C5DI_CATE 3e-13*1.0
{549} C5DIALO2 + HCOCO3 -> C5DI_C2a 3e-13*1.0
{550} C5DIALO2 + HCOCH2O2 -> C5DI_C2b 3e-13*1.0
{551} C5DIALO2 + NPHEN1O2 -> C5DI_NPHb 3e-13*1.0
{552} C5DIALO2 + NNCATECO2 -> C5DI_NNC 6.58e-11*1.0
{553} C5DIALO2 + NCATECO2 -> C5DI_NCAT 2.7e-11*1.0
{554} C5DIALO2 + NBZQO2 -> C5DI_NBZb 3e-13*1.0
{555} C5DIALO2 + PBZQO2 -> C5DI_PBZ 3e-13*1.0
{556} C5DIALO2 + MALANHYO2 -> C5DI_MALc 3e-13*1.0
{557} C5DIALO2 + NDNPHENO2 -> C5DI_NDN 9.08e-11*1.0
{558} C5DIALO2 + DNPHENO2 -> C5DI_DNP 5.2e-11*1.0
{559} C5DIALO2 + HOCH2CO3 -> C5DI_C2c 3e-13*1.0
{560} C5DIALO2 + C5CO2OHCO3 -> C5DI_C5a 3e-13*1.0
{561} C5DIALO2 + C4CO2DBCO3 -> C5DI_C4a 3e-13*1.0
ROOR precursor NPHENO2
{562} NPHENO2 + NPHENO2 -> NPHa_NPHa 9.17e-11*1.0
{563} NPHENO2 + PHENO2 -> NPHa_PHEN 5.29e-11*1.0
{564} NPHENO2 + MALDIALCO3 -> NPHa_MALa 1.16e-12*1.0
{565} NPHENO2 + EPXDLCO3 -> NPHa_EPX 1.5e-11*1.0
{566} NPHENO2 + C3DIALO2 -> NPHa_C3DI 3e-13*1.0

{567} NPHENO2 + MALDIALO2 -> NPHa_MALb 1.67e-11*1.0
{568} NPHENO2 + C6H5O2 -> NPHa_C6a 3e-13*1.0
{569} NPHENO2 + NBZFUO2 -> NPHa_NBZa 5.55e-11*1.0
{570} NPHENO2 + BZFUO2 -> NPHa_BZFU 1.67e-11*1.0
{571} NPHENO2 + HCOCOHCO3 -> NPHa_C3a 4.61e-12*1.0
{572} NPHENO2 + CATEC1O2 -> NPHa_CATE 9.78e-12*1.0
{573} NPHENO2 + HCOCO3 -> NPHa_C2a 3e-13*1.0
{574} NPHENO2 + HCOCH2O2 -> NPHa_C2b 3e-13*1.0
{575} NPHENO2 + NPHEN1O2 -> NPHa_NPHb 3.48e-11*1.0
{576} NPHENO2 + NNCATECO2 -> NPHa_NNC 1e-10*1.0
{577} NPHENO2 + NCATECO2 -> NPHa_NCAT 1e-10*1.0
{578} NPHENO2 + NBZQO2 -> NPHa_NBZb 7.62e-11*1.0
{579} NPHENO2 + PBZQO2 -> NPHa_PBZ 3.74e-11*1.0
{580} NPHENO2 + MALANHYO2 -> NPHa_MALc 2.87e-11*1.0
{581} NPHENO2 + NDNPHENO2 -> NPHa_NDN 1e-10*1.0
{582} NPHENO2 + DNPHENO2 -> NPHa_DNP 1e-10*1.0
{583} NPHENO2 + HOCH2CO3 -> NPHa_C2c 3e-13*1.0
{584} NPHENO2 + C5CO2OHCO3 -> NPHa_C5a 5.12e-11*1.0
{585} NPHENO2 + C4CO2DBCO3 -> NPHa_C4a 2.53e-11*1.0
ROOR precursor PHENO2
{586} PHENO2 + PHENO2 -> PHEN_PHEN 1.41e-11*1.0
{587} PHENO2 + MALDIALCO3 -> PHEN_MALa 3e-13*1.0
{588} PHENO2 + EPXDLCO3 -> PHEN_EPX 3e-13*1.0
{589} PHENO2 + C3DIALO2 -> PHEN_C3DI 3e-13*1.0
{590} PHENO2 + MALDIALO2 -> PHEN_MALb 3e-13*1.0
{591} PHENO2 + C6H5O2 -> PHEN_C6a 3e-13*1.0
{592} PHENO2 + NBZFUO2 -> PHEN_NBZa 1.67e-11*1.0
{593} PHENO2 + BZFUO2 -> PHEN_BZFU 3e-13*1.0
{594} PHENO2 + HCOCOHCO3 -> PHEN_C3a 3e-13*1.0
{595} PHENO2 + CATEC1O2 -> PHEN_CATE 3e-13*1.0
{596} PHENO2 + HCOCO3 -> PHEN_C2a 3e-13*1.0
{597} PHENO2 + HCOCH2O2 -> PHEN_C2b 3e-13*1.0
{598} PHENO2 + NPHEN1O2 -> PHEN_NPHb 3e-13*1.0
{599} PHENO2 + NNCATECO2 -> PHEN_NNC 1e-10*1.0
{600} PHENO2 + NCATECO2 -> PHEN_NCAT 6.67e-11*1.0
{601} PHENO2 + NBZQO2 -> PHEN_NBZb 3.74e-11*1.0
{602} PHENO2 + PBZQO2 -> PHEN_PBZ 3e-13*1.0
{603} PHENO2 + MALANHYO2 -> PHEN_MALc 3e-13*1.0
{604} PHENO2 + NDNPHENO2 -> PHEN_NDN 1e-10*1.0
{605} PHENO2 + DNPHENO2 -> PHEN_DNP 9.17e-11*1.0
{606} PHENO2 + HOCH2CO3 -> PHEN_C2c 3e-13*1.0
{607} PHENO2 + C5CO2OHCO3 -> PHEN_C5a 1.24e-11*1.0
{608} PHENO2 + C4CO2DBCO3 -> PHEN_C4a 3e-13*1.0
ROOR precursor MALDIALCO3
{609} MALDIALCO3 + MALDIALCO3 -> MALa_MALa 3e-13*1.0
{610} MALDIALCO3 + EPXDLCO3 -> MALa_EPX 3e-13*1.0
{611} MALDIALCO3 + C3DIALO2 -> MALa_C3DI 3e-13*1.0
{612} MALDIALCO3 + MALDIALO2 -> MALa_MALb 3e-13*1.0
{613} MALDIALCO3 + C6H5O2 -> MALa_C6a 3e-13*1.0
{614} MALDIALCO3 + NBZFUO2 -> MALa_NBZa 3e-13*1.0

{615} MALDIALCO3 + BZFUO2 -> MALa_BZFU 3e-13*1.0
{616} MALDIALCO3 + HCOCOHCO3 -> MALa_C3a 3e-13*1.0
{617} MALDIALCO3 + CATEC1O2 -> MALa_CATE 3e-13*1.0
{618} MALDIALCO3 + HCOCO3 -> MALa_C2a 3e-13*1.0
{619} MALDIALCO3 + HCOCH2O2 -> MALa_C2b 3e-13*1.0
{620} MALDIALCO3 + NPHEN1O2 -> MALa_NPHb 3e-13*1.0
{621} MALDIALCO3 + NNCATECO2 -> MALa_NNC 5.37e-11*1.0
{622} MALDIALCO3 + NCATECO2 -> MALa_NCAT 1.5e-11*1.0
{623} MALDIALCO3 + NBZQO2 -> MALa_NBZb 3e-13*1.0
{624} MALDIALCO3 + PBZQO2 -> MALa_PBZ 3e-13*1.0
{625} MALDIALCO3 + MALANHYO2 -> MALa_MALc 3e-13*1.0
{626} MALDIALCO3 + NDNPHENO2 -> MALa_NDN 7.87e-11*1.0
{627} MALDIALCO3 + DNPHENO2 -> MALa_DNP 4e-11*1.0
{628} MALDIALCO3 + HOCH2CO3 -> MALa_C2c 3e-13*1.0
{629} MALDIALCO3 + C5CO2OHCO3 -> MALa_C5a 3e-13*1.0
{630} MALDIALCO3 + C4CO2DBCO3 -> MALa_C4a 3e-13*1.0
ROOR precursor EPXDLCO3
{631} EPXDLCO3 + EPXDLCO3 -> EPX_EPX 3e-13*1.0
{632} EPXDLCO3 + C3DIALO2 -> EPX_C3DI 3e-13*1.0
{633} EPXDLCO3 + MALDIALO2 -> EPX_MALb 3e-13*1.0
{634} EPXDLCO3 + C6H5O2 -> EPX_C6a 3e-13*1.0
{635} EPXDLCO3 + NBZFUO2 -> EPX_NBZa 3e-13*1.0
{636} EPXDLCO3 + BZFUO2 -> EPX_BZFU 3e-13*1.0
{637} EPXDLCO3 + HCOCOHCO3 -> EPX_C3a 3e-13*1.0
{638} EPXDLCO3 + CATEC1O2 -> EPX_CATE 3e-13*1.0
{639} EPXDLCO3 + HCOCO3 -> EPX_C2a 3e-13*1.0
{640} EPXDLCO3 + HCOCH2O2 -> EPX_C2b 3e-13*1.0
{641} EPXDLCO3 + NPHEN1O2 -> EPX_NPHb 3e-13*1.0
{642} EPXDLCO3 + NNCATECO2 -> EPX_NNC 6.75e-11*1.0
{643} EPXDLCO3 + NCATECO2 -> EPX_NCAT 2.87e-11*1.0
{644} EPXDLCO3 + NBZQO2 -> EPX_NBZb 3e-13*1.0
{645} EPXDLCO3 + PBZQO2 -> EPX_PBZ 3e-13*1.0
{646} EPXDLCO3 + MALANHYO2 -> EPX_MALc 3e-13*1.0
{647} EPXDLCO3 + NDNPHENO2 -> EPX_NDN 9.25e-11*1.0
{648} EPXDLCO3 + DNPHENO2 -> EPX_DNP 5.37e-11*1.0
{649} EPXDLCO3 + HOCH2CO3 -> EPX_C2c 3e-13*1.0
{650} EPXDLCO3 + C5CO2OHCO3 -> EPX_C5a 3e-13*1.0
{651} EPXDLCO3 + C4CO2DBCO3 -> EPX_C4a 3e-13*1.0
ROOR precursor C3DIALO2
{652} C3DIALO2 + C3DIALO2 -> C3DI_C3DI 3e-13*1.0
{653} C3DIALO2 + MALDIALO2 -> C3DI_MALb 3e-13*1.0
{654} C3DIALO2 + C6H5O2 -> C3DI_C6a 3e-13*1.0
{655} C3DIALO2 + NBZFUO2 -> C3DI_NBZa 3e-13*1.0
{656} C3DIALO2 + BZFUO2 -> C3DI_BZFU 3e-13*1.0
{657} C3DIALO2 + HCOCOHCO3 -> C3DI_C3a 3e-13*1.0
{658} C3DIALO2 + CATEC1O2 -> C3DI_CATE 3e-13*1.0
{659} C3DIALO2 + HCOCO3 -> C3DI_C2a 3e-13*1.0
{660} C3DIALO2 + HCOCH2O2 -> C3DI_C2b 3e-13*1.0
{661} C3DIALO2 + NPHEN1O2 -> C3DI_NPHb 3e-13*1.0
{662} C3DIALO2 + NNCATECO2 -> C3DI_NNC 4.34e-11*1.0

{663} C3DIALO2 + NCATECO2 -> C3DI_NCAT 4.61e-12*1.0
{664} C3DIALO2 + NBZQO2 -> C3DI_NBZb 3e-13*1.0
{665} C3DIALO2 + PBZQO2 -> C3DI_PBZ 3e-13*1.0
{666} C3DIALO2 + MALANHYO2 -> C3DI_MALc 3e-13*1.0
{667} C3DIALO2 + NDNPHENO2 -> C3DI_NDN 6.84e-11*1.0
{668} C3DIALO2 + DNPHENO2 -> C3DI_DNP 2.96e-11*1.0
{669} C3DIALO2 + HOCH2CO3 -> C3DI_C2c 3e-13*1.0
{670} C3DIALO2 + C5CO2OHCO3 -> C3DI_C5a 3e-13*1.0
{671} C3DIALO2 + C4CO2DBCO3 -> C3DI_C4a 3e-13*1.0
ROOR precursor MALDIALO2
{672} MALDIALO2 + MALDIALO2 -> MALb_MALb 3e-13*1.0
{673} MALDIALO2 + C6H5O2 -> MALb_C6a 3e-13*1.0
{674} MALDIALO2 + NBZFUO2 -> MALb_NBZa 3e-13*1.0
{675} MALDIALO2 + BZFUO2 -> MALb_BZFU 3e-13*1.0
{676} MALDIALO2 + HCOCOHCO3 -> MALb_C3a 3e-13*1.0
{677} MALDIALO2 + CATEC1O2 -> MALb_CATE 3e-13*1.0
{678} MALDIALO2 + HCOCO3 -> MALb_C2a 3e-13*1.0
{679} MALDIALO2 + HCOCH2O2 -> MALb_C2b 3e-13*1.0
{680} MALDIALO2 + NPHEN1O2 -> MALb_NPHb 3e-13*1.0
{681} MALDIALO2 + NNCATECO2 -> MALb_NNC 6.93e-11*1.0
{682} MALDIALO2 + NCATECO2 -> MALb_NCAT 3.05e-11*1.0
{683} MALDIALO2 + NBZQO2 -> MALb_NBZb 1.16e-12*1.0
{684} MALDIALO2 + PBZQO2 -> MALb_PBZ 3e-13*1.0
{685} MALDIALO2 + MALANHYO2 -> MALb_MALc 3e-13*1.0
{686} MALDIALO2 + NDNPHENO2 -> MALb_NDN 9.43e-11*1.0
{687} MALDIALO2 + DNPHENO2 -> MALb_DNP 5.55e-11*1.0
{688} MALDIALO2 + HOCH2CO3 -> MALb_C2c 3e-13*1.0
{689} MALDIALO2 + C5CO2OHCO3 -> MALb_C5a 3e-13*1.0
{690} MALDIALO2 + C4CO2DBCO3 -> MALb_C4a 3e-13*1.0
ROOR precursor C6H5O2
{691} C6H5O2 + C6H5O2 -> C6a_C6a 3e-13*1.0
{692} C6H5O2 + NBZFUO2 -> C6a_NBZa 3e-13*1.0
{693} C6H5O2 + BZFUO2 -> C6a_BZFU 3e-13*1.0
{694} C6H5O2 + HCOCOHCO3 -> C6a_C3a 3e-13*1.0
{695} C6H5O2 + CATEC1O2 -> C6a_CATE 3e-13*1.0
{696} C6H5O2 + HCOCO3 -> C6a_C2a 3e-13*1.0
{697} C6H5O2 + HCOCH2O2 -> C6a_C2b 3e-13*1.0
{698} C6H5O2 + NPHEN1O2 -> C6a_NPHb 3e-13*1.0
{699} C6H5O2 + NNCATECO2 -> C6a_NNC 4.86e-11*1.0
{700} C6H5O2 + NCATECO2 -> C6a_NCAT 9.78e-12*1.0
{701} C6H5O2 + NBZQO2 -> C6a_NBZb 3e-13*1.0
{702} C6H5O2 + PBZQO2 -> C6a_PBZ 3e-13*1.0
{703} C6H5O2 + MALANHYO2 -> C6a_MALc 3e-13*1.0
{704} C6H5O2 + NDNPHENO2 -> C6a_NDN 7.36e-11*1.0
{705} C6H5O2 + DNPHENO2 -> C6a_DNP 3.48e-11*1.0
{706} C6H5O2 + HOCH2CO3 -> C6a_C2c 3e-13*1.0
{707} C6H5O2 + C5CO2OHCO3 -> C6a_C5a 3e-13*1.0
{708} C6H5O2 + C4CO2DBCO3 -> C6a_C4a 3e-13*1.0
ROOR precursor NBZFUO2
{709} NBZFUO2 + NBZFUO2 -> NBZa_NBZa 1.93e-11*1.0

{710} NBZFUO2 + BZFUO2 -> NBZa_BZFU 3e-13*1.0
{711} NBZFUO2 + HCOCOHCO3 -> NBZa_C3a 3e-13*1.0
{712} NBZFUO2 + CATEC1O2 -> NBZa_CATE 3e-13*1.0
{713} NBZFUO2 + HCOCO3 -> NBZa_C2a 3e-13*1.0
{714} NBZFUO2 + HCOCH2O2 -> NBZa_C2b 3e-13*1.0
{715} NBZFUO2 + NPHEN1O2 -> NBZa_NPHb 3e-13*1.0
{716} NBZFUO2 + NNCATECO2 -> NBZa_NNC 1e-10*1.0
{717} NBZFUO2 + NCATECO2 -> NBZa_NCAT 6.93e-11*1.0
{718} NBZFUO2 + NBZQO2 -> NBZa_NBZb 4e-11*1.0
{719} NBZFUO2 + PBZQO2 -> NBZa_PBZ 1.16e-12*1.0
{720} NBZFUO2 + MALANHYO2 -> NBZa_MALc 3e-13*1.0
{721} NBZFUO2 + NDNPHENO2 -> NBZa_NDN 1e-10*1.0
{722} NBZFUO2 + DNPHENO2 -> NBZa_DNP 9.43e-11*1.0
{723} NBZFUO2 + HOCH2CO3 -> NBZa_C2c 3e-13*1.0
{724} NBZFUO2 + C5CO2OHCO3 -> NBZa_C5a 1.5e-11*1.0
{725} NBZFUO2 + C4CO2DBCO3 -> NBZa_C4a 3e-13*1.0
ROOR precursor BZFUO2
{726} BZFUO2 + BZFUO2 -> BZFU_BZFU 3e-13*1.0
{727} BZFUO2 + HCOCOHCO3 -> BZFU_C3a 3e-13*1.0
{728} BZFUO2 + CATEC1O2 -> BZFU_CATE 3e-13*1.0
{729} BZFUO2 + HCOCO3 -> BZFU_C2a 3e-13*1.0
{730} BZFUO2 + HCOCH2O2 -> BZFU_C2b 3e-13*1.0
{731} BZFUO2 + NPHEN1O2 -> BZFU_NPHb 3e-13*1.0
{732} BZFUO2 + NNCATECO2 -> BZFU_NNC 6.93e-11*1.0
{733} BZFUO2 + NCATECO2 -> BZFU_NCAT 3.05e-11*1.0
{734} BZFUO2 + NBZQO2 -> BZFU_NBZb 1.16e-12*1.0
{735} BZFUO2 + PBZQO2 -> BZFU_PBZ 3e-13*1.0
{736} BZFUO2 + MALANHYO2 -> BZFU_MALc 3e-13*1.0
{737} BZFUO2 + NDNPHENO2 -> BZFU_NDN 9.43e-11*1.0
{738} BZFUO2 + DNPHENO2 -> BZFU_DNP 5.55e-11*1.0
{739} BZFUO2 + HOCH2CO3 -> BZFU_C2c 3e-13*1.0
{740} BZFUO2 + C5CO2OHCO3 -> BZFU_C5a 3e-13*1.0
{741} BZFUO2 + C4CO2DBCO3 -> BZFU_C4a 3e-13*1.0
ROOR precursor HCOCOHCO3
{742} HCOCOHCO3 + HCOCOHCO3 -> C3a_C3a 3e-13*1.0
{743} HCOCOHCO3 + CATEC1O2 -> C3a_CATE 3e-13*1.0
{744} HCOCOHCO3 + HCOCO3 -> C3a_C2a 3e-13*1.0
{745} HCOCOHCO3 + HCOCH2O2 -> C3a_C2b 3e-13*1.0
{746} HCOCOHCO3 + NPHEN1O2 -> C3a_NPHb 3e-13*1.0
{747} HCOCOHCO3 + NNCATECO2 -> C3a_NNC 5.72e-11*1.0
{748} HCOCOHCO3 + NCATECO2 -> C3a_NCAT 1.84e-11*1.0
{749} HCOCOHCO3 + NBZQO2 -> C3a_NBZb 3e-13*1.0
{750} HCOCOHCO3 + PBZQO2 -> C3a_PBZ 3e-13*1.0
{751} HCOCOHCO3 + MALANHYO2 -> C3a_MALc 3e-13*1.0
{752} HCOCOHCO3 + NDNPHENO2 -> C3a_NDN 8.22e-11*1.0
{753} HCOCOHCO3 + DNPHENO2 -> C3a_DNP 4.34e-11*1.0
{754} HCOCOHCO3 + HOCH2CO3 -> C3a_C2c 3e-13*1.0
{755} HCOCOHCO3 + C5CO2OHCO3 -> C3a_C5a 3e-13*1.0
{756} HCOCOHCO3 + C4CO2DBCO3 -> C3a_C4a 3e-13*1.0
ROOR precursor CATEC1O2

{757}	CATEC1O2 + CATEC1O2 -> CATE_CATE	3e-13*1.0
{758}	CATEC1O2 + HCOCO3 -> CATE_C2a	3e-13*1.0
{759}	CATEC1O2 + HCOCH2O2 -> CATE_C2b	3e-13*1.0
{760}	CATEC1O2 + NPHEN1O2 -> CATE_NPHb	3e-13*1.0
{761}	CATEC1O2 + NNCATECO2 -> CATE_NNC	6.24e-11*1.0
{762}	CATEC1O2 + NCATECO2 -> CATE_NCAT	2.36e-11*1.0
{763}	CATEC1O2 + NBZQO2 -> CATE_NBZb	3e-13*1.0
{764}	CATEC1O2 + PBZQO2 -> CATE_PBZ	3e-13*1.0
{765}	CATEC1O2 + MALANHYO2 -> CATE_MALc	3e-13*1.0
{766}	CATEC1O2 + NDNPHENO2 -> CATE_NDN	8.74e-11*1.0
{767}	CATEC1O2 + DNPHENO2 -> CATE_DNP	4.86e-11*1.0
{768}	CATEC1O2 + HOCH2CO3 -> CATE_C2c	3e-13*1.0
{769}	CATEC1O2 + C5CO2OHCO3 -> CATE_C5a	3e-13*1.0
{770}	CATEC1O2 + C4CO2DBCO3 -> CATE_C4a	3e-13*1.0
ROOR precursor HCOCO3
{771}	HCOCO3 + HCOCO3 -> C2a_C2a	3e-13*1.0
{772}	HCOCO3 + HCOCH2O2 -> C2a_C2b	3e-13*1.0
{773}	HCOCO3 + NPHEN1O2 -> C2a_NPHb	3e-13*1.0
{774}	HCOCO3 + NNCATECO2 -> C2a_NNC	3.13e-11*1.0
{775}	HCOCO3 + NCATECO2 -> C2a_NCAT	3e-13*1.0
{776}	HCOCO3 + NBZQO2 -> C2a_NBZb	3e-13*1.0
{777}	HCOCO3 + PBZQO2 -> C2a_PBZ	3e-13*1.0
{778}	HCOCO3 + MALANHYO2 -> C2a_MALc	3e-13*1.0
{779}	HCOCO3 + NDNPHENO2 -> C2a_NDN	5.63e-11*1.0
{780}	HCOCO3 + DNPHENO2 -> C2a_DNP	1.75e-11*1.0
{781}	HCOCO3 + HOCH2CO3 -> C2a_C2c	3e-13*1.0
{782}	HCOCO3 + C5CO2OHCO3 -> C2a_C5a	3e-13*1.0
{783}	HCOCO3 + C4CO2DBCO3 -> C2a_C4a	3e-13*1.0
ROOR precursor HCOCH2O2
{784}	HCOCH2O2 + HCOCH2O2 -> C2b_C2b	3e-13*1.0
{785}	HCOCH2O2 + NPHEN1O2 -> C2b_NPHb	3e-13*1.0
{786}	HCOCH2O2 + NNCATECO2 -> C2b_NNC	1.93e-11*1.0
{787}	HCOCH2O2 + NCATECO2 -> C2b_NCAT	3e-13*1.0
{788}	HCOCH2O2 + NBZQO2 -> C2b_NBZb	3e-13*1.0
{789}	HCOCH2O2 + PBZQO2 -> C2b_PBZ	3e-13*1.0
{790}	HCOCH2O2 + MALANHYO2 -> C2b_MALc	3e-13*1.0
{791}	HCOCH2O2 + NDNPHENO2 -> C2b_NDN	4.43e-11*1.0
{792}	HCOCH2O2 + DNPHENO2 -> C2b_DNP	5.47e-12*1.0
{793}	HCOCH2O2 + HOCH2CO3 -> C2b_C2c	3e-13*1.0
{794}	HCOCH2O2 + C5CO2OHCO3 -> C2b_C5a	3e-13*1.0
{795}	HCOCH2O2 + C4CO2DBCO3 -> C2b_C4a	3e-13*1.0
ROOR precursor NPHEN1O2
{796}	NPHEN1O2 + NPHEN1O2 -> NPHb_NPHb	3e-13*1.0
{797}	NPHEN1O2 + NNCATECO2 -> NPHb_NNC	8.74e-11*1.0
{798}	NPHEN1O2 + NCATECO2 -> NPHb_NCAT	4.86e-11*1.0
{799}	NPHEN1O2 + NBZQO2 -> NPHb_NBZb	1.93e-11*1.0
{800}	NPHEN1O2 + PBZQO2 -> NPHb_PBZ	3e-13*1.0
{801}	NPHEN1O2 + MALANHYO2 -> NPHb_MALc	3e-13*1.0
{802}	NPHEN1O2 + NDNPHENO2 -> NPHb_NDN	1e-10*1.0
{803}	NPHEN1O2 + DNPHENO2 -> NPHb_DNP	7.36e-11*1.0

{804} NPHEN1O2 + HOCH2CO3 -> NPHb_C2c 3e-13*1.0
{805} NPHEN1O2 + C5CO2OHCO3 -> NPHb_C5a 3e-13*1.0
{806} NPHEN1O2 + C4CO2DBCO3 -> NPHb_C4a 3e-13*1.0
ROOR precursor NNCATECO2
{807} NNCATECO2 + NNCATECO2 -> NNC_NNC 1e-10*1.0
{808} NNCATECO2 + NCATECO2 -> NNC_NCAT 1e-10*1.0
{809} NNCATECO2 + NBZQO2 -> NNC_NBZb 1e-10*1.0
{810} NNCATECO2 + PBZQO2 -> NNC_PBZ 9e-11*1.0
{811} NNCATECO2 + MALANHYO2 -> NNC_MALc 8.13e-11*1.0
{812} NNCATECO2 + NDNPHENO2 -> NNC_NDN 1e-10*1.0
{813} NNCATECO2 + DNPHENO2 -> NNC_DNP 1e-10*1.0
{814} NNCATECO2 + HOCH2CO3 -> NNC_C2c 3.31e-11*1.0
{815} NNCATECO2 + C5CO2OHCO3 -> NNC_C5a 1e-10*1.0
{816} NNCATECO2 + C4CO2DBCO3 -> NNC_C4a 7.79e-11*1.0
ROOR precursor NCATECO2
{817} NCATECO2 + NCATECO2 -> NCAT_NCAT 1e-10*1.0
{818} NCATECO2 + NBZQO2 -> NCAT_NBZb 9e-11*1.0
{819} NCATECO2 + PBZQO2 -> NCAT_PBZ 5.12e-11*1.0
{820} NCATECO2 + MALANHYO2 -> NCAT_MALc 4.25e-11*1.0
{821} NCATECO2 + NDNPHENO2 -> NCAT_NDN 1e-10*1.0
{822} NCATECO2 + DNPHENO2 -> NCAT_DNP 1e-10*1.0
{823} NCATECO2 + HOCH2CO3 -> NCAT_C2c 3e-13*1.0
{824} NCATECO2 + C5CO2OHCO3 -> NCAT_C5a 6.5e-11*1.0
{825} NCATECO2 + C4CO2DBCO3 -> NCAT_C4a 3.91e-11*1.0
ROOR precursor NBZQO2
{826} NBZQO2 + NBZQO2 -> NBZb_NBZb 6.06e-11*1.0
{827} NBZQO2 + PBZQO2 -> NBZb_PBZ 2.19e-11*1.0
{828} NBZQO2 + MALANHYO2 -> NBZb_MALc 1.32e-11*1.0
{829} NBZQO2 + NDNPHENO2 -> NBZb_NDN 1e-10*1.0
{830} NBZQO2 + DNPHENO2 -> NBZb_DNP 1e-10*1.0
{831} NBZQO2 + HOCH2CO3 -> NBZb_C2c 3e-13*1.0
{832} NBZQO2 + C5CO2OHCO3 -> NBZb_C5a 3.56e-11*1.0
{833} NBZQO2 + C4CO2DBCO3 -> NBZb_C4a 9.78e-12*1.0
ROOR precursor PBZQO2
{834} PBZQO2 + PBZQO2 -> PBZ_PBZ 3e-13*1.0
{835} PBZQO2 + MALANHYO2 -> PBZ_MALc 3e-13*1.0
{836} PBZQO2 + NDNPHENO2 -> PBZ_NDN 1e-10*1.0
{837} PBZQO2 + DNPHENO2 -> PBZ_DNP 7.62e-11*1.0
{838} PBZQO2 + HOCH2CO3 -> PBZ_C2c 3e-13*1.0
{839} PBZQO2 + C5CO2OHCO3 -> PBZ_C5a 3e-13*1.0
{840} PBZQO2 + C4CO2DBCO3 -> PBZ_C4a 3e-13*1.0
ROOR precursor MALANHYO2
{841} MALANHYO2 + MALANHYO2 -> MALc_MALc 3e-13*1.0
{842} MALANHYO2 + NDNPHENO2 -> MALc_NDN 1e-10*1.0
{843} MALANHYO2 + DNPHENO2 -> MALc_DNP 6.75e-11*1.0
{844} MALANHYO2 + HOCH2CO3 -> MALc_C2c 3e-13*1.0
{845} MALANHYO2 + C5CO2OHCO3 -> MALc_C5a 3e-13*1.0
{846} MALANHYO2 + C4CO2DBCO3 -> MALc_C4a 3e-13*1.0
ROOR precursor NDNPHENO2
{847} NDNPHENO2 + NDNPHENO2 -> NDN_NDN 1e-10*1.0

{848} NDNPHENO2 + DNPHENO2 -> NDN_DNP 1e-10*1.0
{849} NDNPHENO2 + HOCH2CO3 -> NDN_C2c 5.81e-11*1.0
{850} NDNPHENO2 + C5CO2OHCO3 -> NDN_C5a 1e-10*1.0
{851} NDNPHENO2 + C4CO2DBCO3 -> NDN_C4a 1e-10*1.0
ROOR precursor DNPHENO2
{852} DNPHENO2 + DNPHENO2 -> DNP_DNP 1e-10*1.0
{853} DNPHENO2 + HOCH2CO3 -> DNP_C2c 1.93e-11*1.0
{854} DNPHENO2 + C5CO2OHCO3 -> DNP_C5a 9e-11*1.0
{855} DNPHENO2 + C4CO2DBCO3 -> DNP_C4a 6.41e-11*1.0
ROOR precursor HOCH2CO3
{856} HOCH2CO3 + HOCH2CO3 -> C2c_C2c 3e-13*1.0
{857} HOCH2CO3 + C5CO2OHCO3 -> C2c_C5a 3e-13*1.0
{858} HOCH2CO3 + C4CO2DBCO3 -> C2c_C4a 3e-13*1.0
ROOR precursor C5CO2OHCO3
{859} C5CO2OHCO3 + C5CO2OHCO3 -> C5a_C5a 1.06e-11*1.0
{860} C5CO2OHCO3 + C4CO2DBCO3 -> C5a_C4a 3e-13*1.0
ROOR precursor C4CO2DBCO3
{861} C4CO2DBCO3 + C4CO2DBCO3 -> C4a_C4a 3e-13*1.0

Reaction (R5c)
Peroxy radical reacting with pool of RO_2s forming ROH by O removal and H addition:
{862} BZo_RO2_O7 -> BZo_O4_2OH RO2*2.62e-11*0.21*0.4
{863} BZo_RO2_O9 -> BZo_O6_2OH RO2*5.38e-11*0.21*0.4
{864} BZo_RO2_O11 -> BZo_O8_2OH RO2*8.14e-11*0.21*0.4
{865} BZeo_RO2_O6 -> BZeo_O3_2OH RO2*1.24e-11*0.21*0.4
{866} BZeo_RO2_O8 -> BZeo_O5_2OH RO2*4e-11*0.21*0.4
{867} BZeo_RO2_O10 -> BZeo_O7_2OH RO2*6.76e-11*0.21*0.4
{868} C5_RO2_O6 -> C5e_O3_2OH RO2*2.97e-11*0.21*0.4
{869} C5_RO2_O7 -> C5_O4_2OH RO2*4.34e-11*0.21*0.4
{870} C5_RO2_O8 -> C5e_O5_2OH RO2*5.72e-11*0.21*0.4
{871} C5_RO2_O9 -> C5_O6_2OH RO2*7.1e-11*0.21*0.4
{872} C5_RO2_O10 -> C5e_O7_2OH RO2*8.48e-11*0.21*0.4

Reaction (R5d)
Peroxy radical reacting with pool of RO_2s forming R=O by OH removal:
{873} BZo_RO2_O7 -> BZo_O5_O RO2*2.62e-11*0.2*0.4
{874} BZo_RO2_O9 -> BZo_O7_O RO2*5.38e-11*0.2*0.4
{875} BZo_RO2_O11 -> BZo_O9_O RO2*8.14e-11*0.2*0.4
{876} BZeo_RO2_O6 -> BZeo_O4_O RO2*1.24e-11*0.2*0.4
{877} BZeo_RO2_O8 -> BZeo_O6_O RO2*4e-11*0.2*0.4
{878} BZeo_RO2_O10 -> BZeo_O8_O RO2*6.76e-11*0.2*0.4
{879} C5_RO2_O6 -> C5e_O4_O RO2*2.97e-11*0.2*0.4
{880} C5_RO2_O7 -> C5_O5_O RO2*4.34e-11*0.2*0.4
{881} C5_RO2_O8 -> C5e_O6_O RO2*5.72e-11*0.2*0.4
{882} C5_RO2_O9 -> C5_O7_O RO2*7.1e-11*0.2*0.4
{883} C5_RO2_O10 -> C5e_O8_O RO2*8.48e-11*0.2*0.4

Reaction (R6)
Fragmentation of alkoxy radicals
{884} BZo2_RO_O6 -> GLYOX + MALDIAL + HO2 KDEC*0.05

{885} BZo2_RO_O8 -> GLYOX + MALDIAL + HO2 KDEC*0.05
{886} BZo2_RO_O10 -> GLYOX + MALDIAL + HO2 KDEC*0.05
{887} BZeo2_RO_O5 -> GLYOX + MALDIAL + HO2 KDEC*0.05
{888} BZeo2_RO_O7 -> GLYOX + MALDIAL + HO2 KDEC*0.05
{889} BZeo2_RO_O9 -> GLYOX + MALDIAL + HO2 KDEC*0.05
{890} BZo_RO_O6 -> GLYOX + MALDIAL + HO2 KDEC*0.8
{891} BZo_RO_O8 -> GLYOX + MALDIAL + HO2 KDEC*0.88
{892} BZo_RO_O10 -> GLYOX + MALDIAL + HO2 KDEC*0.43
{893} BZeo_RO_O5 -> GLYOX + MALDIAL + HO2 KDEC*0.1
{894} BZeo_RO_O7 -> GLYOX + MALDIAL + HO2 KDEC*0.64
{895} BZeo_RO_O9 -> GLYOX + MALDIAL + HO2 KDEC*0.1
{896} C5_RO_O5 -> MALDIAL + CO + HO2 KDEC*0.05
{897} C5_RO_O6 -> MALDIAL + CO + HO2 KDEC*0.05
{898} C5_RO_O7 -> MALDIAL + CO + HO2 KDEC*0.05
{899} C5_RO_O8 -> MALDIAL + CO + HO2 KDEC*0.05
{900} C5_RO_O9 -> MALDIAL + CO + HO2 KDEC*0.05

Reaction (R7)
Closed shell formation from RO_2 via H-shift from alpha-hydro(per)oxyl position: RO_2 -> RC=O
{901} BZo_RO2_O7 -> BZouni_O6_O + OH 0.01
{902} BZo_RO2_O9 -> BZouni_O8_O + OH 0.01
{903} BZo_RO2_O11 -> BZouni_O10_O + OH 0.01
{904} BZeo_RO2_O6 -> BZeouni_O5_O + HO2 0.01
{905} BZeo_RO2_O8 -> BZeouni_O7_O + HO2 0.01
{906} BZeo_RO2_O10 -> BZeouni_O9_O + HO2 0.01
{907} C5_RO2_O6 -> C5euni_O5_O + OH 0.3
{908} C5_RO2_O7 -> C5uni_O6_O + OH 0.8
{909} C5_RO2_O8 -> C5euni_O7_O + OH 0.8
{910} C5_RO2_O9 -> C5uni_O8_O + HO2 0.01
{911} C5_RO2_O10 -> C5euni_O9_O + HO2 0.01

Reaction (R8)
Closed shell formation from RO via H-shift from alpha-hydro(per)oxyl position: RO → RC=O
{912} BZo2_RO_O6 -> ROCS_O5_O + HO2 KDEC*0.05
{913} BZo2_RO_O8 -> ROCS_O7_O + HO2 KDEC*0.10
{914} BZo2_RO_O10 -> ROCS_O9_O + HO2 KDEC*0.60
{915} BZo2_RO_O10 -> ROCS_O8_O + HO2 KDEC*0.35
{916} BZeo2_RO_O5 -> ROCS_O4_O + HO2 KDEC*0.05
{917} BZeo2_RO_O7 -> ROCS_O6_O + HO2 KDEC*0.05
{918} BZeo2_RO_O9 -> ROCS_O8_O + HO2 KDEC*0.55
{919} BZo_RO_O6 -> ROCS_O5_O + HO2 KDEC*0.004
{920} BZo_RO_O8 -> ROCS_O7_O + HO2 KDEC*0.001
{921} BZo_RO_O10 -> ROCS_O9_O + HO2 KDEC*0.02
{922} BZeo_RO_O5 -> ROCS_O4_O + HO2 KDEC*0.45
{923} BZeo_RO_O7 -> ROCS_O6_O + HO2 KDEC*0.007
{924} BZeo_RO_O9 -> ROCS_O8_O + HO2 KDEC*0.45
{925} C5_RO_O5 -> C5e_O4_O + HO2 KDEC*0.05
{926} C5_RO_O6 -> C5_O5_O + HO2 KDEC*0.05
{927} C5_RO_O7 -> C5e_O6_O + HO2 KDEC*0.10
{928} C5_RO_O8 -> C5_O7_O + HO2 KDEC*0.25

{929}	C5_RO_O9 -> C5e_O8_O + HO2	KDEC*0.50
{930}	C5_RO_O9 -> C5_O7_O + HO2	KDEC*0.45

Reaction (R9)
Peroxy radical undergoing H-shift and forming carbonyl radical (RC=O*)
RC=O* eliminates CO and adding O_2 to from C5RO2

{931}	BZo_RO2_O7 -> C5_RO2_O8 + CO	0.6
{932}	BZo_RO2_O9 -> C5_RO2_O10 + CO	0.014
{933}	BZeo_RO2_O6 -> C5_RO2_O7 + CO	0.8
{934}	BZeo_RO2_O8 -> C5_RO2_O9 + CO	0.06

References (SI-text)

1 T. Jokinen, M. Sipilä, H. Junninen, M. Ehn, G. Lönn, J. Hakala, T. Petäjä, R. L. Mauldin, M. Kulmala and D. R. Worsnop, Atmospheric sulphuric acid and neutral cluster measurements using CI-APi-TOF, *Atmos. Chem. Phys.*, 2012, **12**, 4117–4125.

2 U. Molteni, F. Bianchi, F. Klein, I. El Haddad, C. Frege, M. J. Rossi, J. Dommen and U. Baltensperger, Formation of highly oxygenated organic molecules from aromatic compounds, *Atmos. Chem. Phys.*, 2018, **18**, 1909–1921.

3 O. Garmash, M. P. Rissanen, I. Pullinen, S. Schmitt, O. Kausiala, R. Tillmann, D. Zhao, C. Percival, T. J. Bannan, M. Priestley, Å. M. Hallquist, E. Kleist, A. Kiendler-Scharr, M. Hallquist, T. Berndt, G. McFiggans, J. Wildt, T. F. Mentel and M. Ehn, Multi-generation OH oxidation as a source for highly oxygenated organic molecules from aromatics, *Atmos. Chem. Phys.*, 2020, **20**, 515–537.

4 L. Xu, K. H. Møller, J. D. Crounse, H. G. Kjaergaard and P. O. Wennberg, New Insights into the Radical Chemistry and Product Distribution in the OH-Initiated Oxidation of Benzene, *Environ. Sci. Technol.*, 2020, **54**, 13467–13477.

5 A. H. Goldstein and I. E. Galbally, Known and Unexplored Organic Constituents in the Earth's Atmosphere, *Environ. Sci. Technol.*, 2007, **41**, 1514–1521.

6 P. D. Lightfoot, R. A. Cox, J. N. Crowley, M. Destriau, G. D. Hayman, M. E. Jenkin, G. K. Moortgat and F. Zabel, Organic peroxy radicals: Kinetics, spectroscopy and tropospheric chemistry, *Atmospheric Environment. Part A. General Topics*, 1992, **26**, 1805–1961.

7 A. Klamt, Conductor-like Screening Model for Real Solvents: A New Approach to the Quantitative Calculation of Solvation Phenomena, *J. Phys. Chem.*, 1995, **99**, 2224–2235.

8 A. Klamt, V. Jonas, T. Bürger and J. C. W. Lohrenz, Refinement and Parametrization of COSMO-RS, *J. Phys. Chem. A*, 1998, **102**, 5074–5085.

9 F. Eckert and A. Klamt, Fast solvent screening via quantum chemistry: COSMO-RS approach, *AIChE J.*, 2002, **48**, 369–385.

10 T. Kurtén, N. Hyttinen, E. L. D'Ambro, J. Thornton and N. L. Prisle, Estimating the saturation vapor pressures of isoprene oxidation products C₅H₁₂O₆ and C₅H₁₀O₆ using COSMO-RS, *Atmos. Chem. Phys.*, 2018, **18**, 17589–17600.

11 *BIOVIA COSMOconf*, 2019.

12 *BIOVIA COSMOtherm*, .

13 N. Hyttinen, I. Pullinen, A. Nissinen, S. Schobesberger, A. Virtanen and T. Yli-Juuti, Comparison of saturation vapor pressures of <i>α</i>-pinene + O₃ oxidation products derived from COSMO-RS computations and thermal desorption experiments, *Atmos. Chem. Phys.*, 2022, **22**, 1195–1208.

14 S. O'Meara, A. M. Booth, M. H. Barley, D. Topping and G. McFiggans, An assessment of vapour pressure estimation methods, *Phys. Chem. Chem. Phys.*, 2014, **16**, 19453–19469.

15 D. Topping, M. Barley, M. K. Bane, N. Higham, B. Aumont, N. Dingle and G. McFiggans, UManSysProp v1.0: an online and open-source facility for molecular property prediction and atmospheric aerosol calculations, *Geosci. Model Dev.*, 2016, **9**, 899–914.

16 S. O'Meara, A. M. Booth, M. H. Barley, D. Topping and G. McFiggans, An assessment of vapour pressure estimation methods, *Phys. Chem. Chem. Phys.*, 2014, **16**, 19453–19469.

17 S. Compernolle, K. Ceulemans and J.-F. Müller, EVAPORATION: a new vapour pressure estimation methodfor organic molecules including non-additivity and intramolecular interactions, *Atmos. Chem. Phys.*, 2011, **11**, 9431–9450.

18 Y. Nannoolal, J. Rarey and D. Ramjugernath, Estimation of pure component properties, *Fluid Phase Equilibria*, 2008, **269**, 117–133.

19 P. B. Myrdal and S. H. Yalkowsky, Estimating Pure Component Vapor Pressures of Complex Organic Molecules, *Ind. Eng. Chem. Res.*, 1997, **36**, 2494–2499.
20 *WHO global air quality guidelines. Particulate matter (PM2.5 and PM10), ozone, nitrogen dioxide, sulfur dioxide and carbon monoxide.*, Wold Health Organization, Geneva, 2021.
21 A. Sekar, G. K. Varghese and M. K. Ravi Varma, Analysis of benzene air quality standards, monitoring methods and concentrations in indoor and outdoor environment, *Heliyon*, 2019, **5**, e02918.

Supplementary Figures

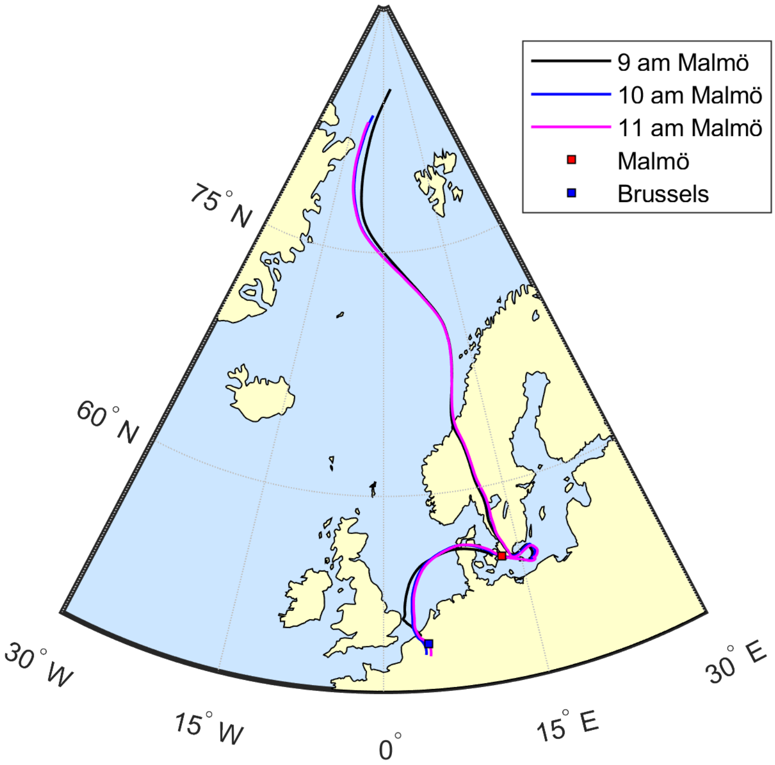

Fig S1 depicts the calculated air-mass trajectories for selected cases named after their local arriving time at Malmö, Sweden on April 28th ("9 am", "10 am" and "11 am"; i.e. UTC + 1 hour).

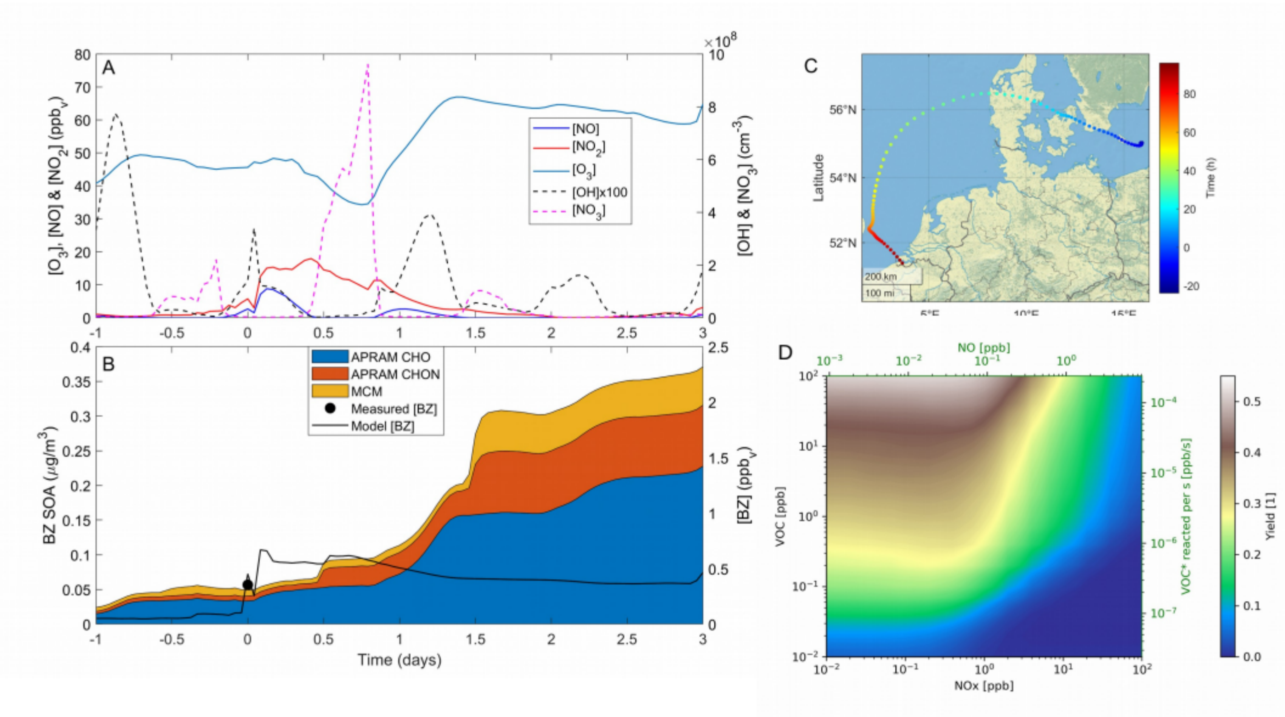

Fig. S2: ADCHEM results along the Case 1 (“9 am”) air mass trajectory. Panel A shows the modelled gas-phase concentrations of NO, NO_2, O_3, OH and NO_3 from 1 day upwind (-1 day) to 3 days downwind Malmö. Panel B shows the modelled benzene gas-phase concentrations ([BZ]) and the modelled benzene SOA mass concentrations of non-nitrate APRAM species, APRAM organonitrates and MCM species. The modelled [BZ] is also compared with the observed benzene concentrations at the measurement station Dalaplan in Malmö. The air mass trajectory path is displayed in panel C. Panel D illustrates the computed mass yield from OH oxidation in the presence of different levels of NO_x and VOC. Bottom and top abscissas depict NOx and NO concentrations. VOC mixing ratio and VOC turnover are shown by the left- and the right-hand ordinate, respectively. The color code indicates the mass yield value.

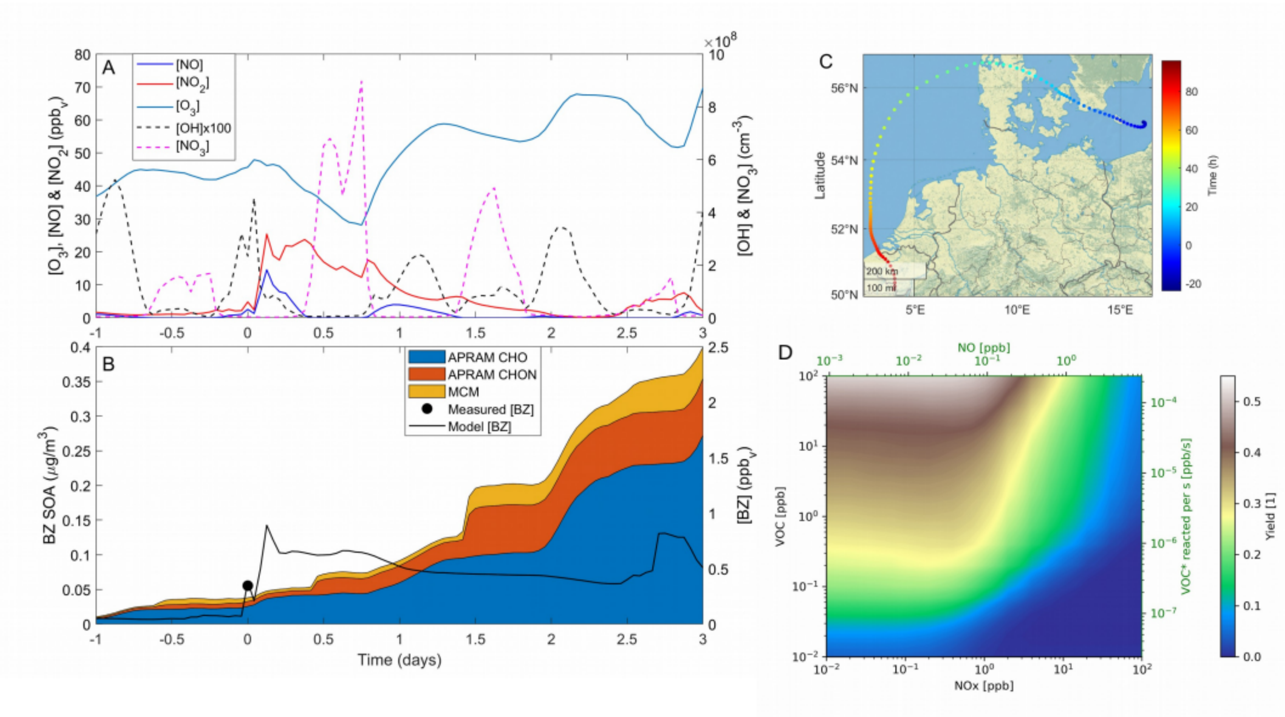

Fig. S3: ADCHEM results along the Case 2 (“10 am”) air mass trajectory. Panel A shows the modelled gas-phase concentrations of NO, NO_2, O_3, OH and NO_3 from 1 day upwind (-1 day) to 3 days downwind Malmö. Panel B shows the modelled benzene gas-phase concentrations ([BZ]) and the modelled benzene SOA mass concentrations of non-nitrate APRAM species, APRAM organonitrates and MCM species. The modelled [BZ] is also compared with the observed benzene concentrations at the measurement station Dalaplan in Malmö. The air mass trajectory path is displayed in panel C. Panel D illustrates the computed mass yield from OH oxidation in the presence of different levels of NO_x and VOC. Bottom and top abscissas depict NOx and NO concentrations. VOC mixing ratio and VOC turnover are shown by the left- and the right-hand ordinate, respectively. The color code indicates the mass yield value.

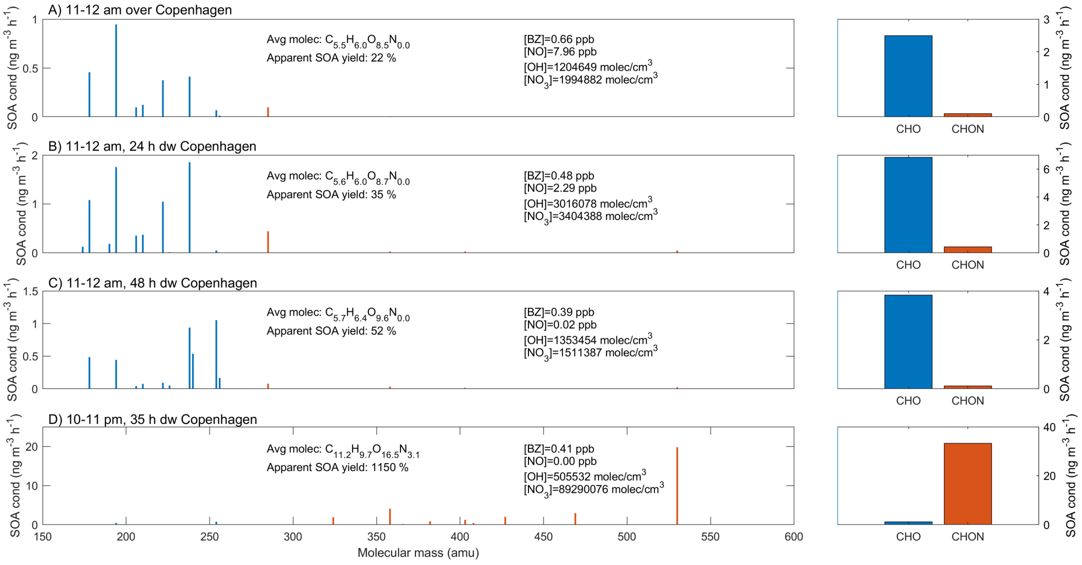

Fig S4: Modelled APRAM benzene SOA mass spectrum of the condensing species during selected 1 hour time slots along the air mass trajectory (Case 1 - "9 am"). Panel A, B and C shows daytime (11-12 am) mass spectrum over the Copenhagen region, 24 hours downwind Copenhagen and 48 hours downwind Copenhagen respectively. Panel D show the mass spectrum which represent the conditions shortly after the sunset (10-11 pm), 35 h downwind Copenhagen. The subpanels to the right show the integrated total APRAM benzene SOA non-nitrate and organonitrate SOA mass increase within the selected time slots. Additional information about the condensing SOA average molecular composition, the apparent SOA yields, the benzene, NO, OH and NO_3 concentrations, and molecular formulas of the major mass peaks are displayed in each panel.

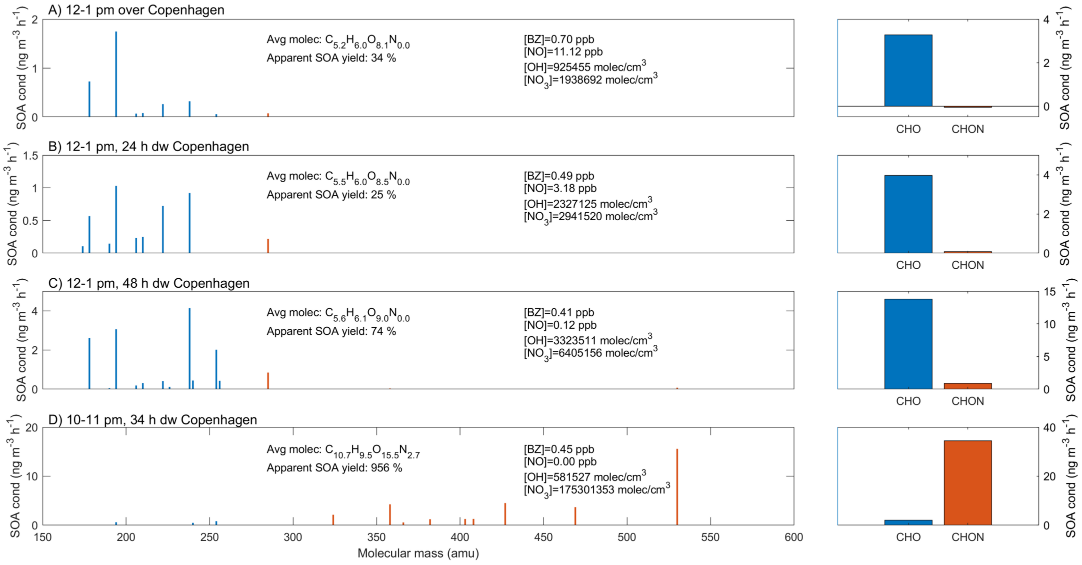

Fig S5: Modelled APRAM benzene SOA mass spectrum of the condensing species during selected 1 hour time slots along the air mass trajectory (Case 2, "10 am"). Panel A, B and C shows daytime (12-1 pm) mass spectrum over the Copenhagen region, 24 hours downwind Copenhagen and 48 hours downwind Copenhagen respectively. Panel D show the mass spectrum which represent the conditions shortly after the sunset (10-11 pm), 34 h downwind Copenhagen. The subpanels to the right show the integrated total APRAM benzene SOA non-nitrate and organonitrate SOA mass increase within the selected time slots. Additional information about the condensing SOA average

molecular composition, the apparent SOA yield, the benzene, NO, OH and NO_3 concentrations, and molecular formulas of the major mass peaks are displayed in each panel.

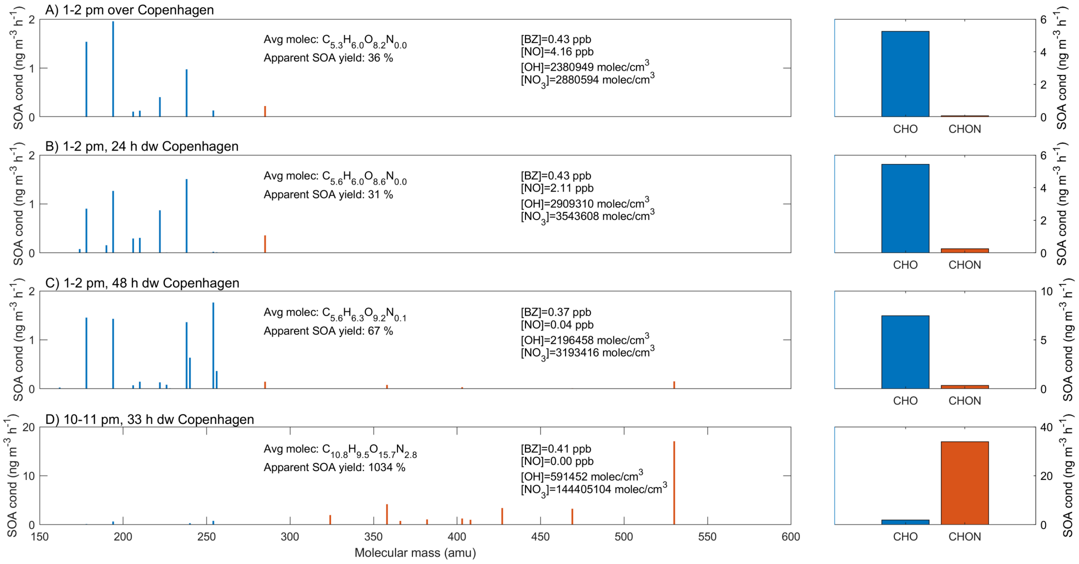

Fig S6: Modelled APRAM benzene SOA mass spectrum of the condensing species during selected 1 hour time slots along the air mass trajectory (Case 3, "11 am"). Panel A, B and C shows daytime (1-2 pm) mass spectrum over the Copenhagen region, 24 hours downwind Copenhagen and 48 hours downwind Copenhagen respectively. Panel D show the mass spectrum which represent the conditions shortly after the sunset (10-11 pm), 33 h downwind Copenhagen. The subpanels to the right show the integrated total APRAM benzene SOA non-nitrate and organonitrate SOA mass increase within the selected time slots. Additional information about the condensing SOA average molecular composition, the apparent SOA yield, the benzene, NO, OH and NO_3 concentrations, and molecular formulas of the major mass peaks are displayed in each panel.

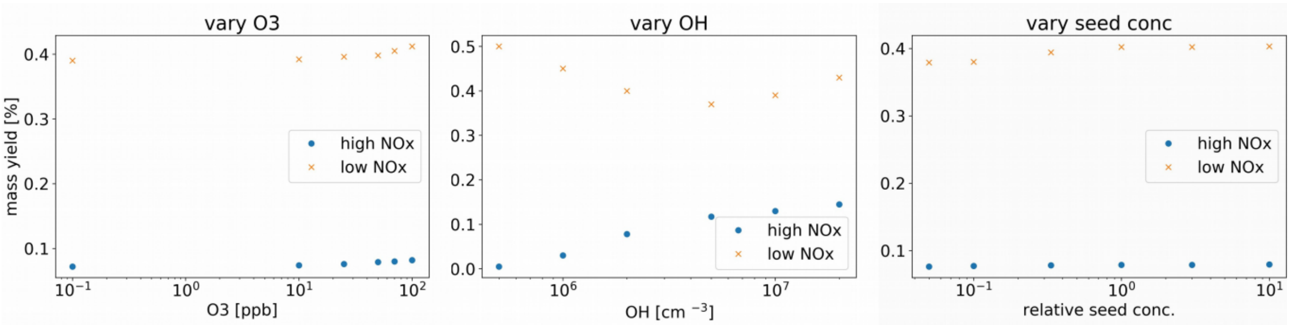

Fig S7: Variation of O_3, OH and relative seed aerosol concentration (base seed aerosol value refers to 10 µg m^{-3} seed aerosol of 55 nm in diameter; concentration is varied by keeping the mass concentration constant and varying seed aerosol diameter which results in a seed aerosol concentration change) relative to the values used in the parametric yield study and impact on the mass yield. Two different VOC/NO_x pairs are considered: "high NO_x" conditions comprise a benzene mixing ratio of 0.3 ppb and 10 ppb of NO_x. A benzene level of 10 ppb and 0.1 ppb NO_x forms the input for the "low NO_x" condition.

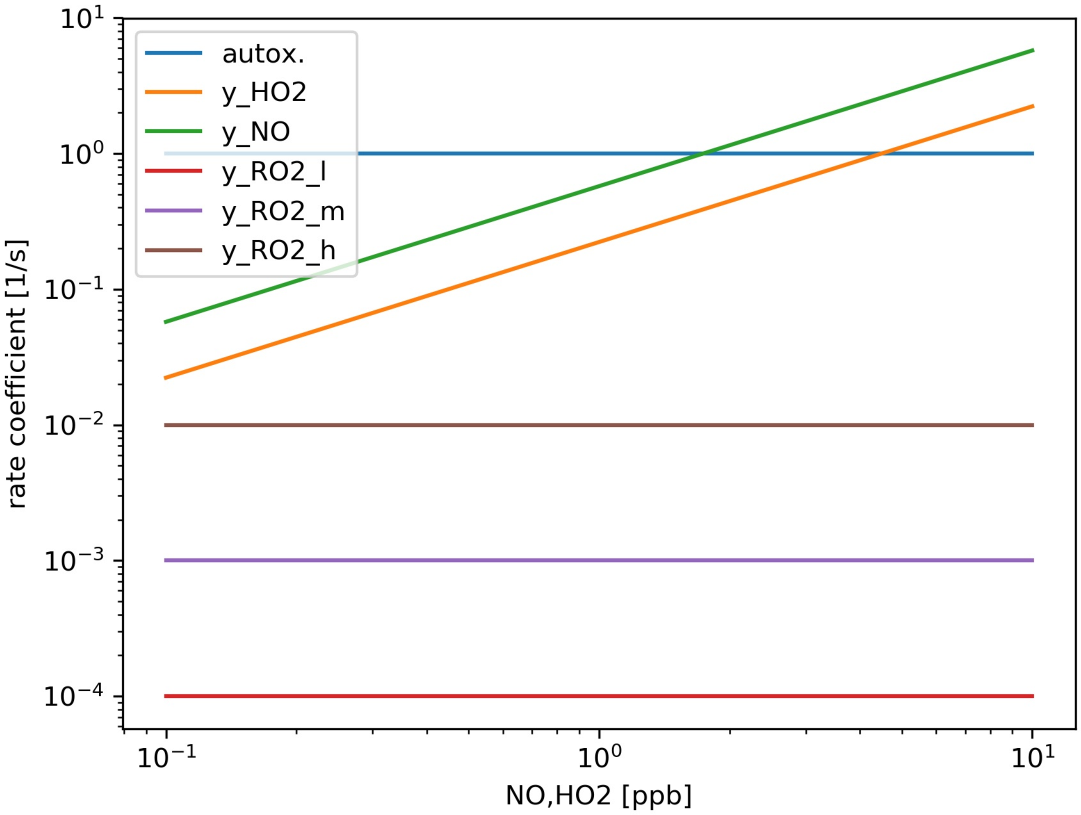

Fig S8: Peroxy radical loss rate (rate coefficient in s^{-1}) for peroxy radical reactions RO_2 + HO_2 (y_HO2), RO_2 + NO (y_NO), RO_2 + RO_2* (y_RO2 _l ,_m ,_h) and autoxidation reaction (assuming a H-shift rate of 1 s-1) at a temperature of 296 K. RO_2* represents the pool of peroxy radicals in the system (assumption [RO2*] = 10^9 cm^{-3}) with three different rate coefficients (kRO2_RO2) for the RO_2 – RO_2 reaction (y_RO2_l: 10^{-13} ; y_RO2_m: 10^{-12}; y_RO2_h : 10^{-11} in $s^{-1}cm^3$, respectively). Yields for the reaction of RO_2 with HO_2 (k_{RO2HO2}) and NO (k_{RO2NO}) are obtained from MCM[1].

[1]See e.g. mcm webpage: http://mcm.york.ac.uk/parameters/simple.htt (visited Oct. 14th 2022)

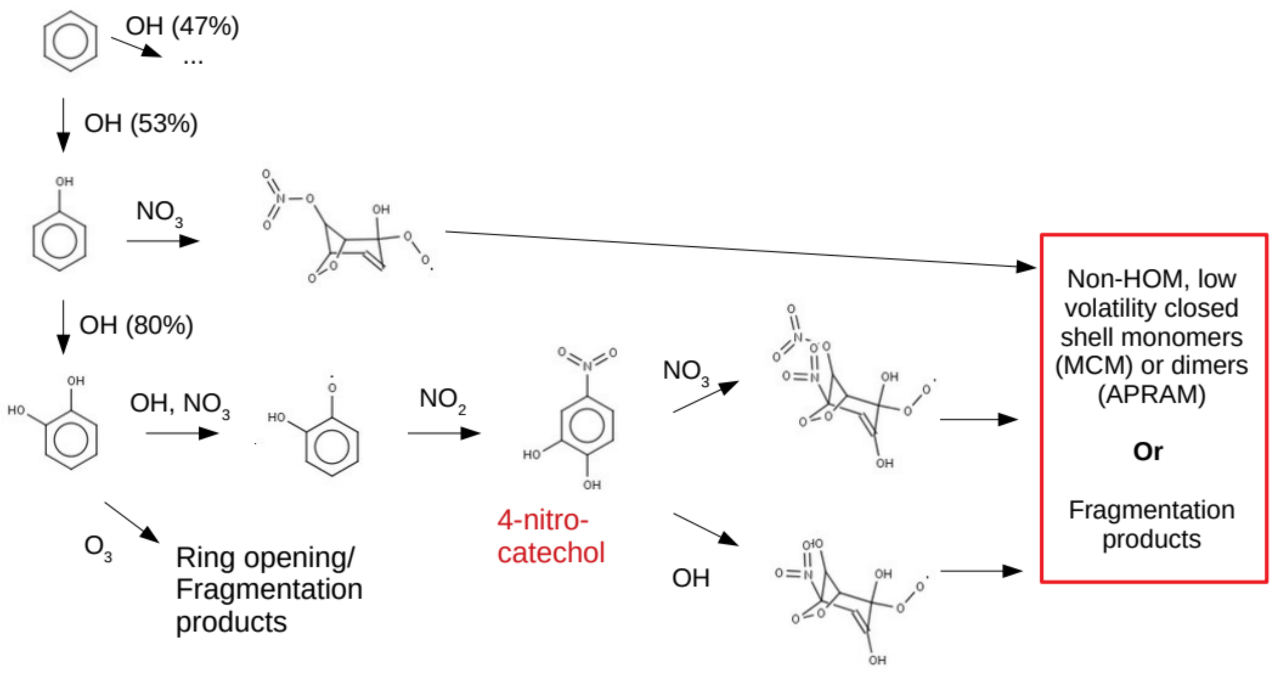

Fig S9: Reaction path in MCM leading to high apparent OH-mass yield at (nighttime) NO_3 radical-oxidation of benzene OH-oxidation products.

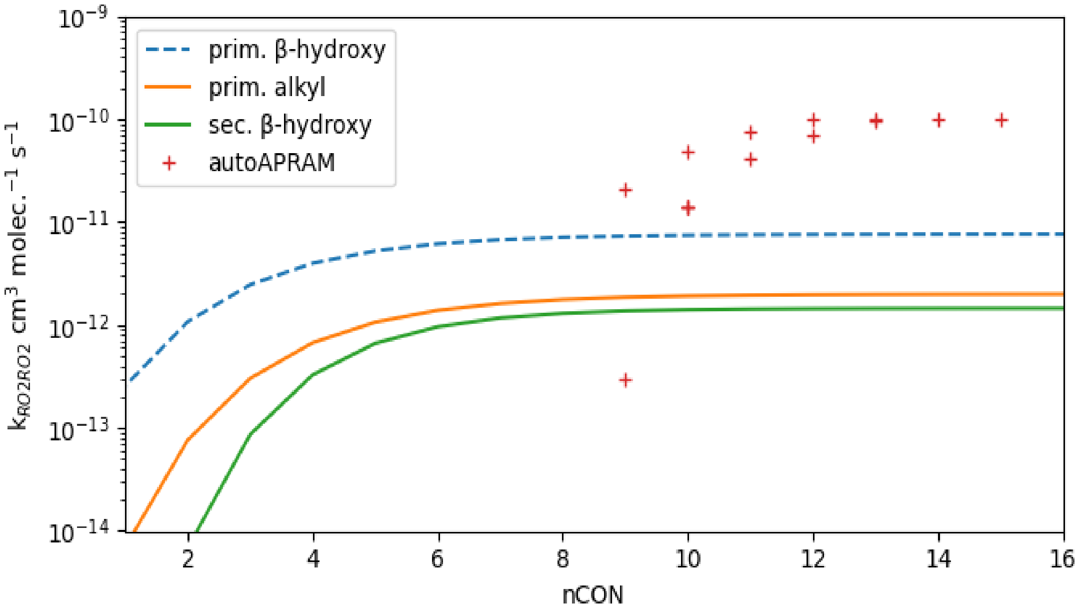

Fig S10: Dimer formation rates, as used in the benzene chemistry from autoAPRAMfw ("autoAPRAM"), compared to SAR-based RO_2-RO_2 rate coefficients provided by Jenkin et al.,[1] for three different peroxy radical types. "nCON" depicts the number of carbon, oxygen and nitrogen atoms in the structure.

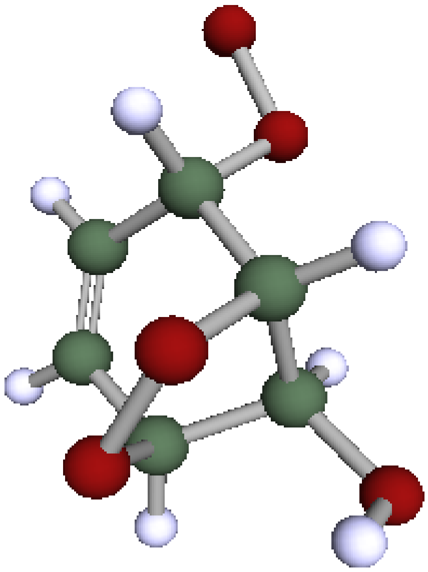

Fig S11: likely peroxy radical structure amongst $C_6H_7O_5$ isomers.

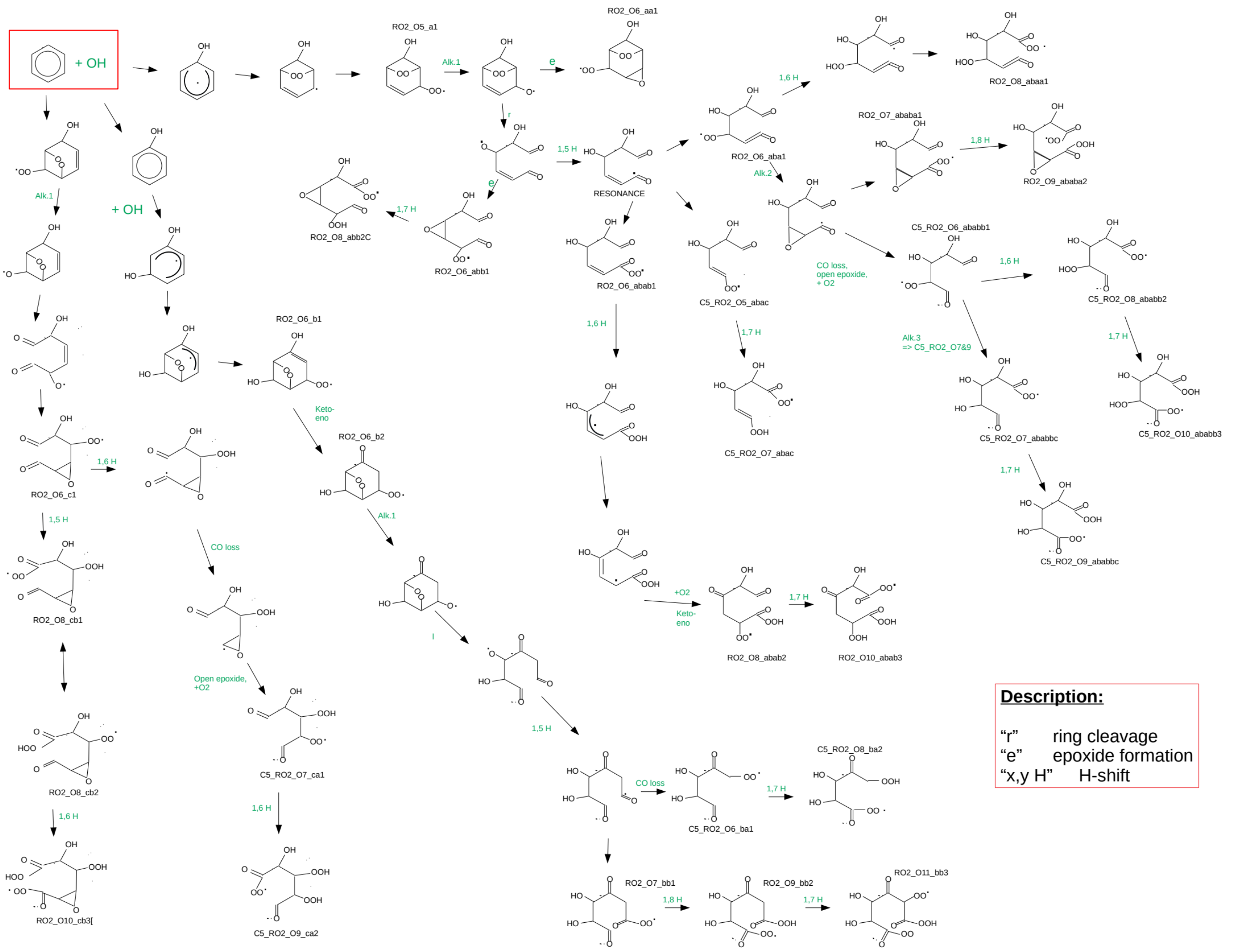

Fig S12/scheme: formation of potential radical structures and the respective reaction pathways. The aim is to suggest structures for the peroxy radicals observed in experiments. These structures serve as input for autoSMILES.

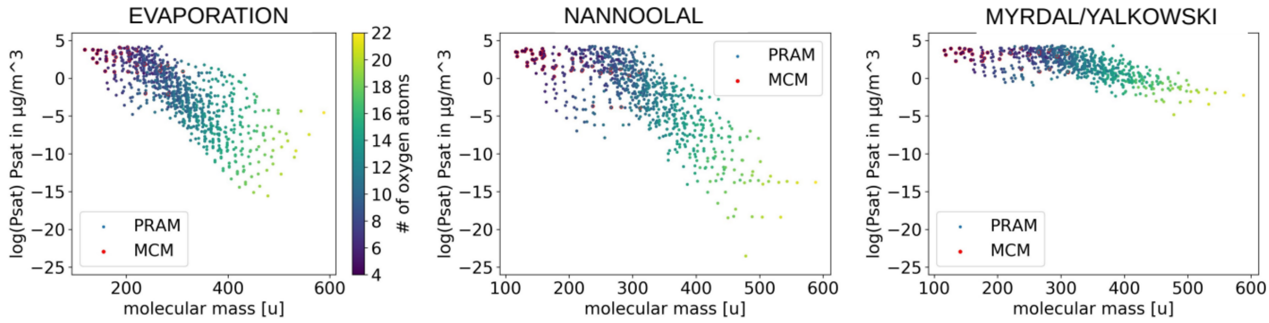

Fig S13: Saturation vapor pressure (ordinate) at room temperature as a function of molecular mass (abscissa) for three different group contribution methods. The colorbar indicates the number of oxygen atoms. Results are distinguished by whether the molecules are mainly formed by MCM(red signs) or autoxidation chemistry (i.e., autoAPRAM-fw species; all other colors).

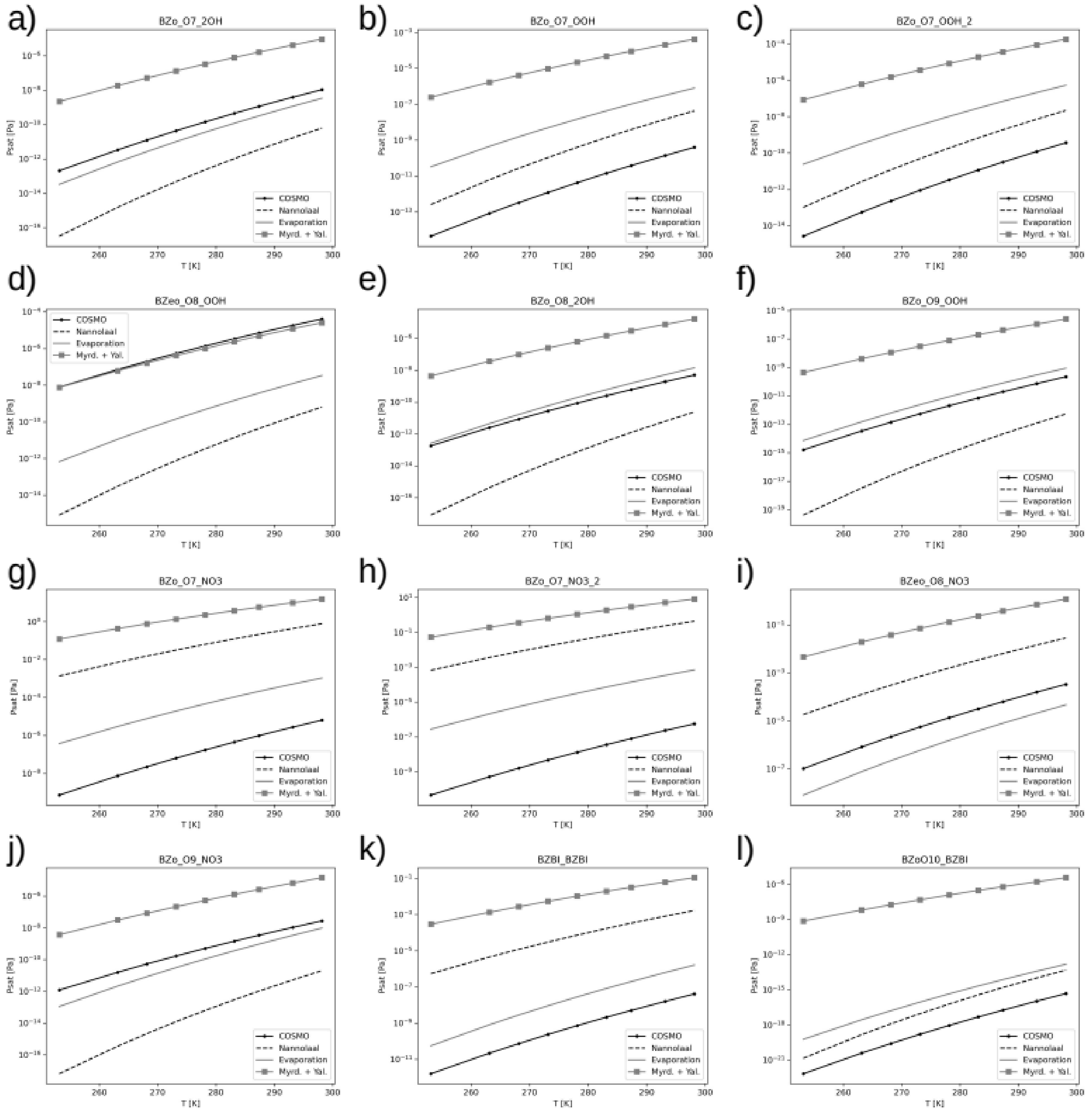

Fig S14: panels a to l depict the comparison of saturation vapor pressure estimates based on three group contribution methods (NANNOOLAL, EVAPORATION, MYRDAL/YALKOWSKI) and COSMO-RS for a temperature range from 250 K to 300 K. Each panel represents a chemical structure. The related SMILES strings defining the isomeric structures are listed in table ST1.

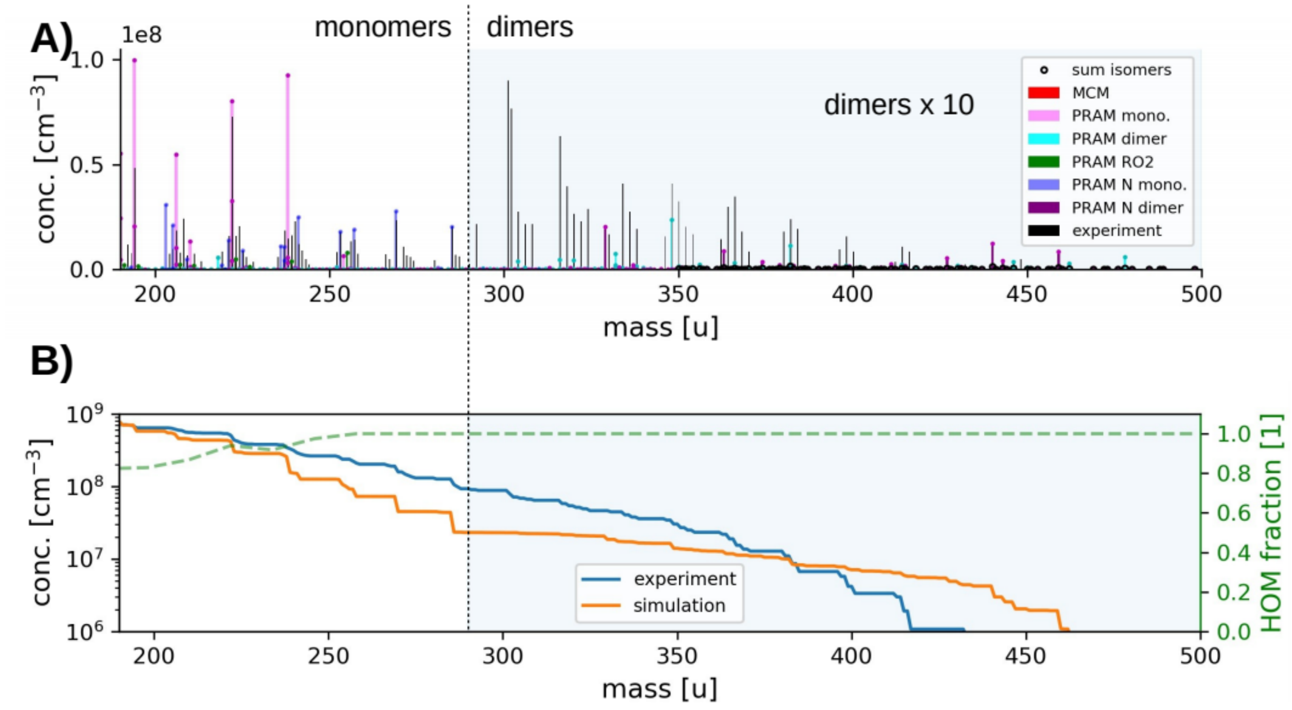

Fig S15: simulation of OH oxidation of benzene in the presence of high mixing ratios of NO_x (> 100 ppb) and UV light. Panel A) depicts the experimental results from chamber experiments[2] (black bars) and the simulation results (colored bars). Nitrogen-containing molecules are marked with a "N". The concentrations of experimentally determined and simulated dimers are raised by a factor 10 to improve readability. Cumulative representation of the model results, starting at the upper observed/simulated molecular mass limit, is shown in panel B). The secondary axis indicates the computed HOM fraction.

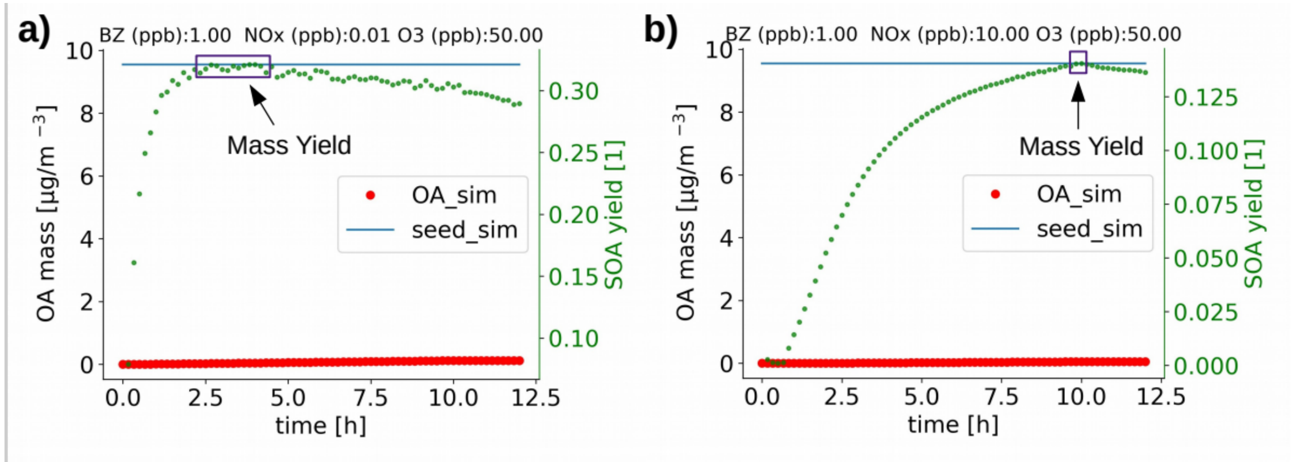

Fig S16: Examples of mass yield evolution curves. Panel a shows a situation where a maximum value is reached within 10 hours simulated. The purple square indicates the data points used for fitting the yield value. In panel b, no maximum is derived after 10 h simulated time. The left hand ordinate depicts the organic aerosol formed in the simulation (OA_sim) as well as the seed aerosol mass (seed_sim); the right hand ordinate represents the according mass yield. Note that the yield is defined as a function of benzene and secondary benzene (OH oxidation of MCM species "BZEPOXMUC" and phenol) consumption, respectively.

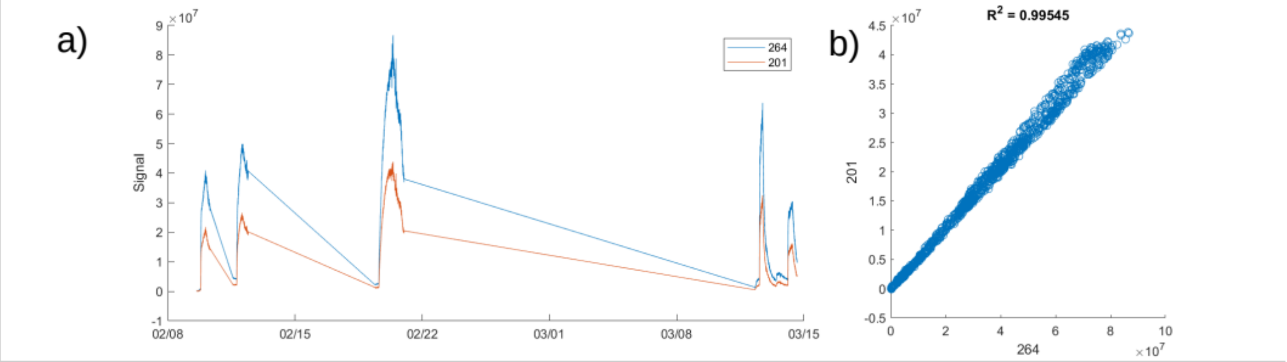

Fig S17 depicts the correlation of species of atomic mass 264 and 201. Panel a) illustrates the evolution of the two species' CIMS signals during experiments conducted at several days. Panel b) shows the correlation of the signals.

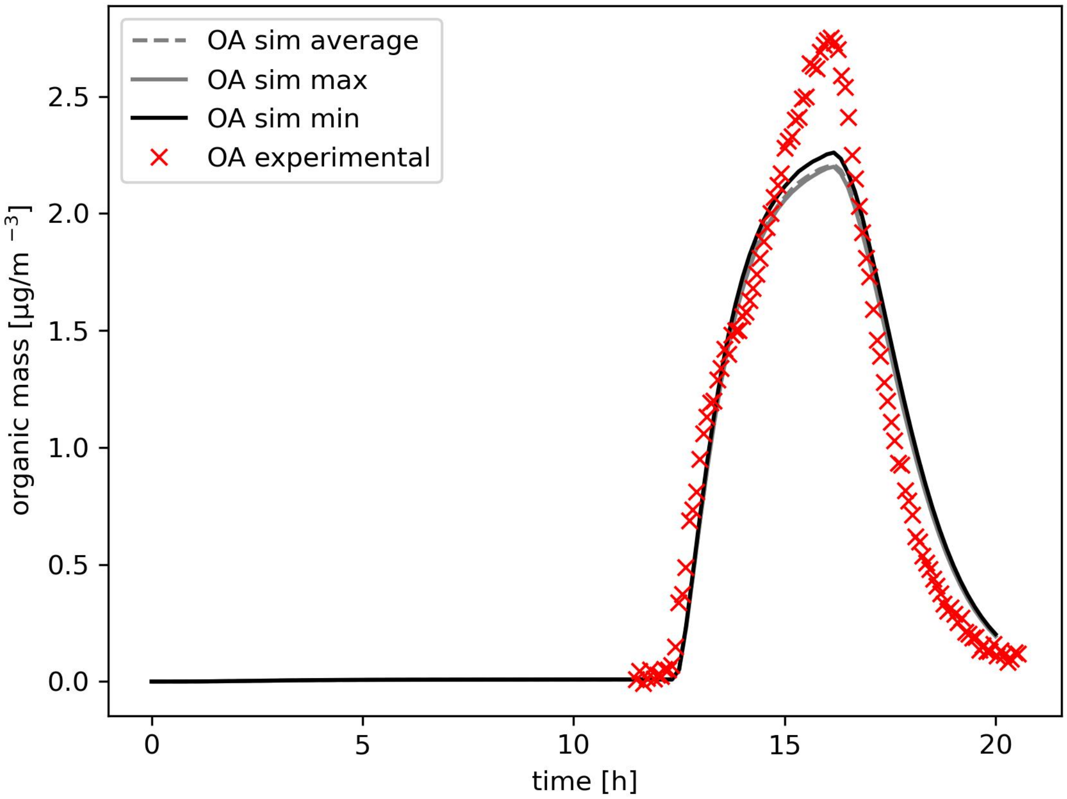

Fig S18 depicts the variation of organic aerosol "OA" simulated (seeded JPAC low-NOx experiment) based on three different sets of underlying RO_2 structures. For each atomic composition, the isomers with lowest ("OA sim min") and highest ("OA sim max") satuartion vapor pressure are considered. An average value of isomeric Psat distribution forms the basis for simulation "OA sim average". The underlying set of structures (including closed shell structures and adducts) is based on RO2 structures described in Fig. S12.

References SI Figures:

1 M. E. Jenkin, R. Valorso, B. Aumont and A. R. Rickard, Estimation of rate coefficients and branching ratios for reactions of organic peroxy radicals for use in automated mechanism construction, *Atmos. Chem. Phys.*, 2019, **19**, 7691–7717.

2 O. Garmash, M. P. Rissanen, I. Pullinen, S. Schmitt, O. Kausiala, R. Tillmann, D. Zhao, C. Percival, T. J. Bannan, M. Priestley, Å. M. Hallquist, E. Kleist, A. Kiendler-Scharr, M. Hallquist, T. Berndt, G. McFiggans, J. Wildt, T. F. Mentel and M. Ehn, Multi-generation OH oxidation as a source for highly oxygenated organic molecules from aromatics, *Atmos. Chem. Phys.*, 2020, **20**, 515–537.

Supplementary Tables:

PRAM Name	Suggested structure	MM
BZBI_BZBI	O(C1C=CC2OOC1C2O)OC1C=CC2OOC1C2O	286.23516
BZo_O7_2OH	O=C(O)C(O)C(=O)CC(OO)C(=O)OO	224.12052
BZo_O7_NO3	O=C(ON(=O)=O)C(O)C(O)C1OC1C(OO)=O	253.11858
BZo_O7_NO3_2	O=C(ON(=O)=O)C(O)C(O)C(=O)CC(=O)OO	253.11858
BZo_O7_OOH	O=C(OO)C(O)C(O)C1OC1C(OO)=O	224.12052
BZo_O7_OOH_2	O=C(OO)C(O)C(O)C(=O)CC(=O)OO	224.12052
BZo_O8_2OH	O=C(OO)C(O)C(O)C(=O)C(O)C(=O)OO	240.11952
BZo_O9_OOH	O=C(OO)C(O)C(O)C(=O)C(OO)C(=O)OO	256.11852
BZoO10_BZBI	O=C(OO)C(O)C(O)C(=O)C(OOC1C=CC2OOC1C2O)C(=O)OO	382.22916
BZeo_O8_OOH	O=C(OO)C(O)C(=O)CC(OO)C(=O)OO	240.11952
BZo_O9_NO3	O=C(OO)C(O)C(O)C(=O)C(ON(=O)=O)C(=O)OO	285.11658
BZeo_O8_NO3	O=C(ON(=O)=O)C(O)C(=O)CC(OO)C(=O)OO	269.11758

Table ST1: List of exemplary structures that is investigated with all saturation vapor pressure methods (COSMO-RS, EVAPORATION, NANNOOLAL, MYRDAL/YALKOWSKI) including the species name, the SMILES description of the structure and the molecular mass (MM).

benzene (ppb)	$10^{-2} – 10^{2}$ ppb_V
NOx (ppb)	$10^{-2} – 10^{2}$ ppb_V
methane	2 ppm_V
CO	200 ppb_V
NO	0.1 * NO2
SO_2	10 ppt_V
sunlight Intensity	Equiv. to const. OH of 10^6 cm^{-3}
temperature	293.15 K
relative humidity	60%
pressure	10^5 Pa
model Δt [s]	5

Table ST2: overview of input parameters for the parametric yield study under atmospheric conditions. Benzene and NOx levels are varied in the given range while other parameters remain constant.

OH [cm^{-3}]	8.5E+11
HO_2 [cm^{-3}]	1.7E+12
Benzene [cm^{-3}]	9.85E+13
O_3 [cm^{-3}]	3.36E+12
Temp. [K]	298.13
RH [%]	40
residence time [s]	20
tube diameter [cm]	7.4

model Δt [s]	0.1

Table ST3: initial values for gas phase species entering the flow tube. Temperature (Temp.) and relative humidity (RH) are considered constant while traveling through the tube, while the gas phase chemical species are allowed to evolve.

Chamber Vol. [m^3]	1.45
inflow [l/min]	30
model Δt [s]	10
RH [%]	65
T [K]	287.35
simulation time [h]	20
Benzene [ppb_V]	15.4
O_3 [ppb_V]	58
NO [ppb_V]	1.075
NO_2 [ppb_V]	3.225
SO_2 [ppb_V]	0.01
CO [ppb_V]	1

Table ST4: inputs for simulating gas phase chemistry experiments in JPAC chamber under high NOx conditions. The mean residence time is 45 min. Concentration of species listed in the table are considered constant throughout the simulation (i.e. flow reactor experiment).

Chamber Vol. [m^3]	1.45
in-/outflow [l/min]	30
model Δt [s]	10
RH [%]	65
T [K]	287.35
simulation time [h]	20
Benzene [ppb_V]	15.7
O_3 [ppb_V]	42
NO [ppb_V]	0.0001
NO_2 [ppb_V]	0.001
SO_2 [ppb_V]	0.01
CO [ppb_V]	1
seed addition time [h]	12.4 – 16.1
$d_{p,m}$ [nm]	100
σ_g	1.2
double charged fraction [%]	25

Table ST5: inputs for simulating SOA formation experiments in JPAC chamber. The mean residence time is 45 min. Seed aerosol is added for a period of roughly 3.7 hours, 12.4 hours after the start of the experiment. Concentration of species listed in the table are considered constant throughout the simulation (i.e. flow reactor experiment).

	low NO_x	High NO_x
Chamber Vol. [m^3]	28	28

outflow [l/min]	5	5
model Δt [s]	10	10
RH [%]	5	5
T [K]	295	295
simulation time [h]	7	7
Benzene [ppb_V]	395	337
O_3 [ppb_V]	15	simulated
H_2O_2 [ppm_V]	4	--
HONO [ppb_V]	--	350
NO [ppb_V]	0.0001	83
NO_2 [ppb_V]	0.001	86
SO_2 [ppb_V]	0.01	0.01
CO [ppb_V]	1	1
$d_{p,m}$ [nm]	55	55
σ_g	1.2	1.2
double charged fraction [%]	25	25

Table ST6: summary of model inputs for simulations of CALTECH chamber under low and high NO_x conditions, respectively. The chemical species and seed aerosol are added at the start of the experiment/simulation (i.e. batch-mode experiment). Oxidants form from photoloysing precursors (H_2O_2 and HONO). The light spectrum was obtained from Cocker et al.,.[1]

autoAPRAM-fw name	C	H	O	N	Structure (SMILES-format)
BZo_RO2_O7	6	7	7	0	O=CC(O)C(O)C1OC1C(O[O])=O
BZo_RO2_O9	6	7	9	0	O=C(O[O])C(O)C(O)C1OC1C(OO)=O
BZo_RO2_O11	6	7	11	0	O=C(OO)C(O)C(O)C(=O)C(O[O])C(=O)OO
BZeo_RO2_O6	6	7	6	0	[O]OC1C2OC2C3OOC1C3(O)
BZeo_RO2_O8	6	7	8	0	O=C=CC(OO)C(O)C(O)C(=O)O[O]
BZeo_RO2_O10	6	7	10	0	O=C(O[O])C(O)C(=O)CC(OO)C(=O)OO
C5_RO2_O6	5	7	6	0	O=CC(O[O])C(O)C(O)C=O
C5_RO2_O7	5	7	7	0	OOC=CC(O)C(O)C(=O)O[O]
C5_RO2_O8	5	7	8	0	O=CC(OO)C(O)C(O)C(O[O])=O
C5_RO2_O9	5	7	9	0	O=C(O[O])C(O)C(O)C(O)C(=O)OO
C5_RO2_O10	5	7	10	0	O=C(O[O])C(OO)C(O)C(O)C(OO)=O
BZo_O4_2OH	6	8	6	0	O=CC(O)C(O)C1OC1C(O)=O
BZo_O6_2OH	6	8	8	0	O=C(O)C(O)C(O)C1OC1C(OO)=O
BZo_O8_2OH	6	8	10	0	O=C(OO)C(O)C(O)C(=O)C(O)C(=O)OO
BZeo_O3_2OH	6	8	5	0	OC1C2OC2C3OOC1C3(O)
BZeo_O5_2OH	6	8	7	0	O=C=CC(OO)C(O)C(O)C(=O)O
BZeo_O7_2OH	6	8	9	0	O=C(O)C(O)C(=O)CC(OO)C(=O)OO
C5e_O3_2OH	5	8	5	0	O=CC(O)C(O)C(O)C=O
C5_O4_2OH	5	8	6	0	OOC=CC(O)C(O)C(=O)O
C5e_O5_2OH	5	8	7	0	O=CC(OO)C(O)C(O)C(O)=O
C5_O6_2OH	5	8	8	0	O=C(O)C(O)C(O)C(O)C(=O)OO
C5e_O7_2OH	5	8	9	0	O=C(O)C(OO)C(O)C(O)C(OO)=O
BZo_O5_O	6	6	6	0	O=CC(=O)C(O)C1OC1C(O)=O
BZo_O7_O	6	6	8	0	O=C(O)C(=O)C(O)C1OC1C(OO)=O
BZo_O9_O	6	6	10	0	O=C(OO)C(O)C(O)C(=O)C(=O)C(=O)OO
BZeo_O4_O	6	6	5	0	O=C1C2OC2C3OOC1C3(O)
BZeo_O6_O	6	6	7	0	O=C=CC(OO)C(=O)C(O)C(=O)O
BZeo_O8_O	6	6	9	0	O=C(O)C(=O)C(=O)CC(OO)C(=O)OO
C5e_O4_O	5	6	5	0	O=CC(=O)C(O)C(O)C=O
C5_O5_O	5	6	6	0	OOC=CC(=O)C(O)C(=O)O
C5e_O6_O	5	6	7	0	O=CC(OO)C(=O)C(O)C(O)=O
C5_O7_O	5	6	8	0	O=C(O)C(=O)C(O)C(O)C(=O)OO
C5e_O8_O	5	6	9	0	O=C(O)C(OO)C(=O)C(O)C(OO)=O
BZouni_O6_O	6	6	7	0	O=CC(=O)C(O)C1OC1C(OO)=O
BZouni_O8_O	6	6	9	0	O=C(OO)C(=O)C(O)C1OC1C(OO)=O
BZouni_O10_O	6	6	11	0	O=C(OO)C(=O)C(O)C(=O)C(OO)C(=O)OO
BZeouni_O5_O	6	6	6	0	OOC1C2OC2C3OOC1C3(=O)
BZeouni_O7_O	6	6	8	0	O=C=CC(OO)C(=O)C(O)C(=O)OO
BZeouni_O9_O	6	6	10	0	O=C(OO)C(=O)C(=O)CC(OO)C(=O)OO

C5euni_O5_O	5	6	6	0	O=CC(OO)C(=O)C(O)C=O
C5uni_O6_O	5	6	7	0	OOC=CC(=O)C(O)C(=O)OO
C5euni_O7_O	5	6	8	0	O=CC(OO)C(=O)C(O)C(OO)=O
C5uni_O8_O	5	6	9	0	O=C(OO)C(=O)C(O)C(O)C(=O)OO
C5euni_O9_O	5	6	10	0	O=C(OO)C(OO)C(=O)C(O)C(OO)=O
BZo_RO_O6	6	7	6	0	O=CC(O)C(O)C1OC1C([O])=O
BZo_RO_O8	6	7	8	0	O=C([O])C(O)C(O)C1OC1C(OO)=O
BZo_RO_O10	6	7	10	0	O=C(OO)C(O)C(O)C(=O)C([O])C(=O)OO
BZeo_RO_O5	6	7	5	0	[O]C1C2OC2C3OOC1C3(O)
BZeo_RO_O7	6	7	7	0	O=C=CC(OO)C(O)C(O)C(=O)[O]
BZeo_RO_O9	6	7	9	0	O=C([O])C(O)C(=O)CC(OO)C(=O)OO
BZoO6_BZoO6	12	14	12	0	O=CC(O)C(O)C1OC1C(=O)(OO(C(=O)C1OC1C(O)C(O)C=O))
BZoO6_BZoO8	12	14	14	0	O=CC(O)C(O)C1OC1C(=O)(OO(C(C(O)C(O)C1OC1C(OO)=O)=O))
BZoO6_BZoO10	12	14	16	0	O=CC(O)C(O)C1OC1C(=O)(OO(C(C(=O)OO)C(=O)C(O)C(O)C(OO)=O))
BZoO6_BZeoO5	12	14	11	0	O=CC(O)C(O)C1OC1C(=O)(OOC1C2OC2C3OOC1C3(O))
BZoO6_BZeoO7	12	14	13	0	O=CC(O)C(O)C1OC1C(=O)(OO(C(=O)C(O)C(O)C(OO)C=C=O))
BZoO6_BZeoO9	12	14	15	0	O=CC(O)C(O)C1OC1C(=O)(OO(C(C(O)C(=O)CC(OO)C(=O)OO)=O))
BZoO6_C5eO5	11	14	11	0	O=CC(O)C(O)C1OC1C(=O)(OO(C(C(O)C(O)C=O)C=O))
BZoO6_C5O6	11	14	12	0	O=CC(O)C(O)C1OC1C(=O)(OO(C(=O)C(O)C(O)C=COO))
BZoO6_C5eO7	11	14	13	0	O=CC(O)C(O)C1OC1C(=O)(OO(C(=O)C(O)C(O)C(OO)C=O))
BZoO6_C5O8	11	14	14	0	O=CC(O)C(O)C1OC1C(=O)(OO(C(C(O)C(O)C(O)C(=O)OO)=O))
BZoO6_C5eO9	11	14	15	0	O=CC(O)C(O)C1OC1C(=O)(OO(C(C(OO)C(O)C(O)C(OO)=O)=O))
BZoO6_BZBI	12	14	10	0	O=CC(O)C(O)C1OC1C(=O)(OOC1C=CC2OOC1C2O)
BZoO6_BZMUa	12	12	10	0	O=CC(O)C(O)C1OC1C(=O)(OOC(=O)C1OC1C=CC=O)
BZoO6_BZMUb	12	14	11	0	O=CC(O)C(O)C1OC1C(=O)(OO(C(C1OC1C=O)C(O)C=O))
BZoO6_C5DI	11	12	9	0	O=CC(O)C(O)C1OC1C(=O)(OO(C(C=O)C=CC=O))
BZoO6_NPHa	12	13	13	1	O=CC(O)C(O)C1OC1C(=O)(OOC1(O)C=CC2OOC1C2ON(=O)=O)
BZoO6_PHEN	12	14	11	0	O=CC(O)C(O)C1OC1C(=O)(OOC1(O)C=CC2OOC1C2O)
BZoO6_MALa	10	10	9	0	O=CC(O)C(O)C1OC1C(=O)(OOC(=O)C=CC=O)
BZoO6_EPX	10	10	10	0	O=CC(O)C(O)C1OC1C(=O)(OOC(=O)C1OC1C=O)
BZoO6_C3DI	9	10	9	0	O=CC(O)C(O)C1OC1C(=O)(OOC(C=O)C=O)
BZoO6_MALb	10	12	10	0	O=CC(O)C(O)C1OC1C(=O)(OOC(C=O)C(O)C=O)
BZoO6_C6a	12	12	7	0	O=CC(O)C(O)C1OC1C(=O)(OOc1ccccc1)
BZoO6_NBZa	10	11	12	1	O=CC(O)C(O)C1OC1C(=O)(OOC1C(=O)OCC1ON(=O)=O)
BZoO6_BZFU	10	12	10	0	O=CC(O)C(O)C1OC1C(=O)(OOC1C(O)COC1=O)
BZoO6_C3a	9	10	10	0	O=CC(O)C(O)C1OC1C(=O)(OO(C(=O)C(C=O)O))
BZoO6_CATE	12	12	8	0	O=CC(O)C(O)C1OC1C(=O)(OOc1ccccc1O)
BZoO6_C2a	8	8	9	0	O=CC(O)C(O)C1OC1C(=O)(OOC(=O)C=O)
BZoO6_C2b	8	10	8	0	O=CC(O)C(O)C1OC1C(=O)(OOCC=O)
BZoO6_NPHb	12	11	9	1	O=CC(O)C(O)C1OC1C(=O)(OOc1ccccc1N(=O)=O)
BZoO6_NNC	12	12	16	2	O=CC(O)C(O)C1OC1C(=O)(OOC1(O)C(=CC2(OOC1C2ON(=O)=O)N(=O)=O)O)
BZoO6_NCAT	12	13	14	1	O=CC(O)C(O)C1OC1C(=O)(OOC1(O)C(=CC2(OOC1C2O)N(=O)=O)O)
BZoO6_NBZb	12	11	12	1	O=CC(O)C(O)C1OC1C(=O)(OOC1C(=O)C=CC(=O)C1ON(=O)=O)
BZoO6_PBZ	12	12	10	0	O=CC(O)C(O)C1OC1C(=O)(OOC1C(=O)C=CC(=O)C1O)
BZoO6_MALc	10	10	11	0	O=CC(O)C(O)C1OC1C(=O)(OOC1C(=O)OC(=O)C1O)
BZoO6_NDN	12	11	17	3	O=CC(O)C(O)C1OC1C(=O)(OOC1(O)C(=CC2(OOC1C2ON(=O)=O)N(=O)=O)N(=O)=O)
BZoO6_DNP	12	12	15	2	O=CC(O)C(O)C1OC1C(=O)(OOC1(O)C(=CC2(OOC1C2O)N(=O)=O)N(=O)=O)
BZoO6_C2c	8	10	9	0	O=CC(O)C(O)C1OC1C(=O)(OO(C(=O)CO))
BZoO6_C5a	12	12	11	0	O=CC(O)C(O)C1OC1C(=O)(OO(C(=O)C=CC(=O)C(C=O)O))
BZoO6_C4a	11	10	10	0	O=CC(O)C(O)C1OC1C(=O)(OOC(=O)C=CC(=O)C=O)
BZoO8_BZoO8	12	14	16	0	O=C(OO(C(C(O)C(O)C1OC1C(OO)=O)=O))C(O)C(O)C1OC1C(OO)=O
BZoO8_BZoO10	12	14	18	0	O=C(OO(C(C(=O)OO)C(=O)C(O)C(O)C(OO)=O))C(O)C(O)C1OC1C(OO)=O
BZoO8_BZeoO5	12	14	13	0	O=C(OOC1C2OC2C3OOC1C3(O))C(O)C(O)C1OC1C(OO)=O
BZoO8_BZeoO7	12	14	15	0	O=C(OO(C(=O)C(O)C(O)C(OO)C=C=O))C(O)C(O)C1OC1C(OO)=O
BZoO8_BZeoO9	12	14	17	0	O=C(OO(C(C(O)C(=O)CC(OO)C(=O)OO)=O))C(O)C(O)C1OC1C(OO)=O
BZoO8_C5eO5	11	14	13	0	O=C(OO(C(C(O)C(O)C=O)C=O))C(O)C(O)C1OC1C(OO)=O
BZoO8_C5O6	11	14	14	0	O=C(OO(C(=O)C(O)C(O)C=COO))C(O)C(O)C1OC1C(OO)=O
BZoO8_C5eO7	11	14	15	0	O=C(OO(C(=O)C(O)C(O)C(OO)C=O))C(O)C(O)C1OC1C(OO)=O
BZoO8_C5O8	11	14	16	0	O=C(OO(C(C(O)C(O)C(O)C(=O)OO)=O))C(O)C(O)C1OC1C(OO)=O
BZoO8_C5eO9	11	14	17	0	O=C(OO(C(C(OO)C(O)C(O)C(OO)=O)=O))C(O)C(O)C1OC1C(OO)=O
BZoO8_BZBI	12	14	12	0	O=C(OOC1C=CC2OOC1C2O)C(O)C(O)C1OC1C(OO)=O
BZoO8_BZMUa	12	12	12	0	O=C(OOC(=O)C1OC1C=CC=O)C(O)C(O)C1OC1C(OO)=O
BZoO8_BZMUb	12	14	13	0	O=C(OO(C(C1OC1C=O)C(O)C=O))C(O)C(O)C1OC1C(OO)=O
BZoO8_C5DI	11	12	11	0	O=C(OO(C(C=O)C=CC=O))C(O)C(O)C1OC1C(OO)=O
BZoO8_NPHa	12	13	15	1	O=C(OOC1(O)C=CC2OOC1C2ON(=O)=O)C(O)C(O)C1OC1C(OO)=O
BZoO8_PHEN	12	14	13	0	O=C(OOC1(O)C=CC2OOC1C2O)C(O)C(O)C1OC1C(OO)=O
BZoO8_MALa	10	10	11	0	O=C(OOC(=O)C=CC=O)C(O)C(O)C1OC1C(OO)=O
BZoO8_EPX	10	10	12	0	O=C(OOC(=O)C1OC1C=O)C(O)C(O)C1OC1C(OO)=O
BZoO8_C3DI	9	10	11	0	O=C(OOC(C=O)C=O)C(O)C(O)C1OC1C(OO)=O
BZoO8_MALb	10	12	12	0	O=C(OOC(C=O)C(O)C=O)C(O)C(O)C1OC1C(OO)=O
BZoO8_C6a	12	12	9	0	O=C(OOc1ccccc1)C(O)C(O)C1OC1C(OO)=O
BZoO8_NBZa	10	11	14	1	O=C(OOC1C(=O)OCC1ON(=O)=O)C(O)C(O)C1OC1C(OO)=O
BZoO8_BZFU	10	12	12	0	O=C(OOC1C(O)COC1=O)C(O)C(O)C1OC1C(OO)=O
BZoO8_C3a	9	10	12	0	O=C(OO(C(=O)C(C=O)O))C(O)C(O)C1OC1C(OO)=O
BZoO8_CATE	12	12	10	0	O=C(OOc1ccccc1O)C(O)C(O)C1OC1C(OO)=O

BZoO8_C2a	8	8	11	0	O=C(OOC(=O)C=O)C(O)C(O)C1OC1C(OO)=O
BZoO8_C2b	8	10	10	0	O=C(OOCC=O)C(O)C(O)C1OC1C(OO)=O
BZoO8_NPHb	12	11	11	1	O=C(OOc1ccccc1N(=O)=O)C(O)C(O)C1OC1C(OO)=O
BZoO8_NNC	12	12	18	2	O=C(OOC1(O)C(=CC2(OOC1C2ON(=O)=O)N(=O)=O)O)C(O)C(O)C1OC1C(OO)=O
BZoO8_NCAT	12	13	16	1	O=C(OOC1(O)C(=CC2(OOC1C2O)N(=O)=O)O)C(O)C(O)C1OC1C(OO)=O
BZoO8_NBZb	12	11	14	1	O=C(OOC1C(=O)C=CC(=O)C1ON(=O)=O)C(O)C(O)C1OC1C(OO)=O
BZoO8_PBZ	12	12	12	0	O=C(OOC1C(=O)C=CC(=O)C1O)C(O)C(O)C1OC1C(OO)=O
BZoO8_MALc	10	10	13	0	O=C(OOC1C(=O)OC(=O)C1O)C(O)C(O)C1OC1C(OO)=O
BZoO8_NDN	12	11	19	3	O=C(OOC1(O)C(=CC2(OOC1C2ON(=O)=O)N(=O)=O)N(=O)=O)C(O)C(O)C1OC1C(OO)=O
BZoO8_DNP	12	12	17	2	O=C(OOC1(O)C(=CC2(OOC1C2O)N(=O)=O)N(=O)=O)C(O)C(O)C1OC1C(OO)=O
BZoO8_C2c	8	10	11	0	O=C(OO(C(=O)CO))C(O)C(O)C1OC1C(OO)=O
BZoO8_C5a	12	12	13	0	O=C(OO(C(=O)C=CC(=O)C(C=O)O))C(O)C(O)C1OC1C(OO)=O
BZoO8_C4a	11	10	12	0	O=C(OOC(=O)C=CC(=O)C=O)C(O)C(O)C1OC1C(OO)=O
BZoO10_BZoO10	12	14	20	0	O=C(OO)C(O)C(O)C(=O)C(OO(C(C(=O)OO)C(=O)C(O)C(O)C(OO)=O))C(=O)OO
BZoO10_BZeoO5	12	14	15	0	O=C(OO)C(O)C(O)C(=O)C(OOC1C2OC2C3OOC1C3(O))C(=O)OO
BZoO10_BZeoO7	12	14	17	0	O=C(OO)C(O)C(O)C(=O)C(OO(C(=O)C(O)C(O)C(OO)C=C=O))C(=O)OO
BZoO10_BZeoO9	12	14	19	0	O=C(OO)C(O)C(O)C(=O)C(OO(C(C(O)C(=O)CC(OO)C(=O)OO)=O))C(=O)OO
BZoO10_C5eO5	11	14	15	0	O=C(OO)C(O)C(O)C(=O)C(OO(C(C(O)C(O)C=O)C=O))C(=O)OO
BZoO10_C5O6	11	14	16	0	O=C(OO)C(O)C(O)C(=O)C(OO(C(=O)C(O)C(O)C=COO))C(=O)OO
BZoO10_C5eO7	11	14	17	0	O=C(OO)C(O)C(O)C(=O)C(OO(C(=O)C(O)C(O)C(OO)C=O))C(=O)OO
BZoO10_C5O8	11	14	18	0	O=C(OO)C(O)C(O)C(=O)C(OO(C(C(O)C(O)C(O)C(=O)OO)=O))C(=O)OO
BZoO10_C5eO9	11	14	19	0	O=C(OO)C(O)C(O)C(=O)C(OO(C(C(OO)C(O)C(O)C(OO)=O)=O))C(=O)OO
BZoO10_BZBI	12	14	14	0	O=C(OO)C(O)C(O)C(=O)C(OOC1C=CC2OOC1C2O)C(=O)OO
BZoO10_BZMUa	12	12	14	0	O=C(OO)C(O)C(O)C(=O)C(OOC(=O)C1OC1C=CC=O)C(=O)OO
BZoO10_BZMUb	12	14	15	0	O=C(OO)C(O)C(O)C(=O)C(OO(C(C1OC1C=O)C(O)C=O))C(=O)OO
BZoO10_C5DI	11	12	13	0	O=C(OO)C(O)C(O)C(=O)C(OO(C(C=O)C=CC=O))C(=O)OO
BZoO10_NPHa	12	13	17	1	O=C(OO)C(O)C(O)C(=O)C(OOC1(O)C=CC2OOC1C2ON(=O)=O)C(=O)OO
BZoO10_PHEN	12	14	15	0	O=C(OO)C(O)C(O)C(=O)C(OOC1(O)C=CC2OOC1C2O)C(=O)OO
BZoO10_MALa	10	10	13	0	O=C(OO)C(O)C(O)C(=O)C(OOC(=O)C=CC=O)C(=O)OO
BZoO10_EPX	10	10	14	0	O=C(OO)C(O)C(O)C(=O)C(OOC(=O)C1OC1C=O)C(=O)OO
BZoO10_C3DI	9	10	13	0	O=C(OO)C(O)C(O)C(=O)C(OOC(C=O)C=O)C(=O)OO
BZoO10_MALb	10	12	14	0	O=C(OO)C(O)C(O)C(=O)C(OOC(C=O)C(O)C=O)C(=O)OO
BZoO10_C6a	12	12	11	0	O=C(OO)C(O)C(O)C(=O)C(OOc1ccccc1)C(=O)OO
BZoO10_NBZa	10	11	16	1	O=C(OO)C(O)C(O)C(=O)C(OOC1C(=O)OCC1ON(=O)=O)C(=O)OO
BZoO10_BZFU	10	12	14	0	O=C(OO)C(O)C(O)C(=O)C(OOC1C(O)COC1=O)C(=O)OO
BZoO10_C3a	9	10	14	0	O=C(OO)C(O)C(O)C(=O)C(OO(C(=O)C(C=O)O))C(=O)OO
BZoO10_CATE	12	12	12	0	O=C(OO)C(O)C(O)C(=O)C(OOc1ccccc1O)C(=O)OO
BZoO10_C2a	8	8	13	0	O=C(OO)C(O)C(O)C(=O)C(OOC(=O)C=O)C(=O)OO
BZoO10_C2b	8	10	12	0	O=C(OO)C(O)C(O)C(=O)C(OOCC=O)C(=O)OO
BZoO10_NPHb	12	11	13	1	O=C(OO)C(O)C(O)C(=O)C(OOc1ccccc1N(=O)=O)C(=O)OO
BZoO10_NNC	12	12	20	2	O=C(OO)C(O)C(O)C(=O)C(OOC1(O)C(=CC2(OOC1C2ON(=O)=O)N(=O)=O)O)C(=O)OO
BZoO10_NCAT	12	13	18	1	O=C(OO)C(O)C(O)C(=O)C(OOC1(O)C(=CC2(OOC1C2O)N(=O)=O)O)C(=O)OO
BZoO10_NBZb	12	11	16	1	O=C(OO)C(O)C(O)C(=O)C(OOC1C(=O)C=CC(=O)C1ON(=O)=O)C(=O)OO
BZoO10_PBZ	12	12	14	0	O=C(OO)C(O)C(O)C(=O)C(OOC1C(=O)C=CC(=O)C1O)C(=O)OO
BZoO10_MALc	10	10	15	0	O=C(OO)C(O)C(O)C(=O)C(OOC1C(=O)OC(=O)C1O)C(=O)OO
BZoO10_NDN	12	11	21	3	O=C(OO)C(O)C(O)C(=O)C(OOC1(O)C(=CC2(OOC1C2ON(=O)=O)N(=O)=O)N(=O)=O)C(=O)OO
BZoO10_DNP	12	12	19	2	O=C(OO)C(O)C(O)C(=O)C(OOC1(O)C(=CC2(OOC1C2O)N(=O)=O)N(=O)=O)C(=O)OO
BZoO10_C2c	8	10	13	0	O=C(OO)C(O)C(O)C(=O)C(OO(C(=O)CO))C(=O)OO
BZoO10_C5a	12	12	15	0	O=C(OO)C(O)C(O)C(=O)C(OO(C(=O)C=CC(=O)C(C=O)O))C(=O)OO
BZoO10_C4a	11	10	14	0	O=C(OO)C(O)C(O)C(=O)C(OOC(=O)C=CC(=O)C=O)C(=O)OO
BZeoO5_BZeoO5	12	14	10	0	O(C1C2OC2C3OOC1C3(O))OC1C2OC2C3OOC1C3(O)
BZeoO5_BZeoO7	12	14	12	0	O=C=CC(OO)C(O)C(O)C(=O)(OOC1C2OC2C3OOC1C3(O))
BZeoO5_BZeoO9	12	14	14	0	O=C(OOC1C2OC2C3OOC1C3(O))C(O)C(=O)CC(OO)C(=O)OO
BZeoO5_C5eO5	11	14	10	0	O=CC(OOC1C2OC2C3OOC1C3(O))C(O)C(O)C=O
BZeoO5_C5O6	11	14	11	0	OOC=CC(O)C(O)C(=O)(OOC1C2OC2C3OOC1C3(O))
BZeoO5_C5eO7	11	14	12	0	O=CC(OO)C(O)C(O)C(=O)(OOC1C2OC2C3OOC1C3(O))
BZeoO5_C5O8	11	14	13	0	O=C(OOC1C2OC2C3OOC1C3(O))C(O)C(O)C(O)C(=O)OO
BZeoO5_C5eO9	11	14	14	0	O=C(OOC1C2OC2C3OOC1C3(O))C(OO)C(O)C(O)C(OO)=O
BZeoO5_BZBI	12	14	9	0	O(C1C2OC2C3OOC1C3(O))OC1C=CC2OOC1C2O
BZeoO5_BZMUa	12	12	9	0	O(C1C2OC2C3OOC1C3(O))OC(=O)C1OC1C=CC=O
BZeoO5_BZMUb	12	14	10	0	O=CC(O)C(OOC1C2OC2C3OOC1C3(O))C1OC1C=O
BZeoO5_C5DI	11	12	8	0	O=CC=CC(OOC1C2OC2C3OOC1C3(O))C=O
BZeoO5_NPHa	12	13	12	1	O(C1C2OC2C3OOC1C3(O))OC1(O)C=CC2OOC1C2ON(=O)=O
BZeoO5_PHEN	12	14	10	0	O(C1C2OC2C3OOC1C3(O))OC1(O)C=CC2OOC1C2O
BZeoO5_MALa	10	10	8	0	O(C1C2OC2C3OOC1C3(O))OC(=O)C=CC=O
BZeoO5_EPX	10	10	9	0	O(C1C2OC2C3OOC1C3(O))OC(=O)C1OC1C=O
BZeoO5_C3DI	9	10	8	0	O(C1C2OC2C3OOC1C3(O))OC(C=O)C=O
BZeoO5_MALb	10	12	9	0	O(C1C2OC2C3OOC1C3(O))OC(C=O)C(O)C=O
BZeoO5_C6a	12	12	6	0	O(C1C2OC2C3OOC1C3(O))Oc1ccccc1
BZeoO5_NBZa	10	11	11	1	O(C1C2OC2C3OOC1C3(O))OC1C(=O)OCC1ON(=O)=O
BZeoO5_BZFU	10	12	9	0	O(C1C2OC2C3OOC1C3(O))OC1C(O)COC1=O
BZeoO5_C3a	9	10	9	0	OC(C=O)C(=O)(OOC1C2OC2C3OOC1C3(O))
BZeoO5_CATE	12	12	7	0	O(C1C2OC2C3OOC1C3(O))Oc1ccccc1O
BZeoO5_C2a	8	8	8	0	O(C1C2OC2C3OOC1C3(O))OC(=O)C=O
BZeoO5_C2b	8	10	7	0	O(C1C2OC2C3OOC1C3(O))OCC=O

BZeoO5_NPHb	12	11	8	1	O(C1C2OC2C3OOC1C3(O))Oc1ccccc1N(=O)=O
BZeoO5_NNC	12	12	15	2	O(C1C2OC2C3OOC1C3(O))OC1(O)C(=CC2(OOC1C2ON(=O)=O)N(=O)=O)O
BZeoO5_NCAT	12	13	13	1	O(C1C2OC2C3OOC1C3(O))OC1(O)C(=CC2(OOC1C2O)N(=O)=O)O
BZeoO5_NBZb	12	11	11	1	O(C1C2OC2C3OOC1C3(O))OC1C(=O)C=CC(=O)C1ON(=O)=O
BZeoO5_PBZ	12	12	9	0	O(C1C2OC2C3OOC1C3(O))OC1C(=O)C=CC(=O)C1O
BZeoO5_MALc	10	10	10	0	O(C1C2OC2C3OOC1C3(O))OC1C(=O)OC(=O)C1O
BZeoO5_NDN	12	11	16	3	O(C1C2OC2C3OOC1C3(O))OC1(O)C(=CC2(OOC1C2ON(=O)=O)N(=O)=O)N(=O)=O
BZeoO5_DNP	12	12	14	2	O(C1C2OC2C3OOC1C3(O))OC1(O)C(=CC2(OOC1C2O)N(=O)=O)N(=O)=O
BZeoO5_C2c	8	10	8	0	OCC(=O)(OOC1C2OC2C3OOC1C3(O))
BZeoO5_C5a	12	12	10	0	OC(C=O)C(=O)C=CC(=O)(OOC1C2OC2C3OOC1C3(O))
BZeoO5_C4a	11	10	9	0	O(C1C2OC2C3OOC1C3(O))OC(=O)C=CC(=O)C=O
BZeoO7_BZeoO7	12	14	14	0	O=C=CC(OO)C(O)C(O)C(=O)(OO(C(=O)C(O)C(O)C(OO)C=C=O))
BZeoO7_BZeoO9	12	14	16	0	O=C=CC(OO)C(O)C(O)C(=O)(OO(C(C(O)C(=O)CC(OO)C(=O)OO)=O))
BZeoO7_C5eO5	11	14	12	0	O=C=CC(OO)C(O)C(O)C(=O)(OO(C(C(O)C(O)C=O)C=O))
BZeoO7_C5O6	11	14	13	0	O=C=CC(OO)C(O)C(O)C(=O)(OO(C(=O)C(O)C(O)C=COO))
BZeoO7_C5eO7	11	14	14	0	O=C=CC(OO)C(O)C(O)C(=O)(OO(C(=O)C(O)C(O)C(OO)C=O))
BZeoO7_C5O8	11	14	15	0	O=C=CC(OO)C(O)C(O)C(=O)(OO(C(C(O)C(O)C(O)C(=O)OO)=O))
BZeoO7_C5eO9	11	14	16	0	O=C=CC(OO)C(O)C(O)C(=O)(OO(C(C(OO)C(O)C(O)C(OO)=O)=O))
BZeoO7_BZBI	12	14	11	0	O=C=CC(OO)C(O)C(O)C(=O)(OOC1C=CC2OOC1C2O)
BZeoO7_BZMUa	12	12	11	0	O=C=CC(OO)C(O)C(O)C(=O)(OOC(=O)C1OC1C=CC=O)
BZeoO7_BZMUb	12	14	12	0	O=C=CC(OO)C(O)C(O)C(=O)(OO(C(C1OC1C=O)C(O)C=O))
BZeoO7_C5DI	11	12	10	0	O=C=CC(OO)C(O)C(O)C(=O)(OO(C(C=O)C=CC=O))
BZeoO7_NPHa	12	13	14	1	O=C=CC(OO)C(O)C(O)C(=O)(OOC1(O)C=CC2OOC1C2ON(=O)=O)
BZeoO7_PHEN	12	14	12	0	O=C=CC(OO)C(O)C(O)C(=O)(OOC1(O)C=CC2OOC1C2O)
BZeoO7_MALa	10	10	10	0	O=C=CC(OO)C(O)C(O)C(=O)(OOC(=O)C=CC=O)
BZeoO7_EPX	10	10	11	0	O=C=CC(OO)C(O)C(O)C(=O)(OOC(=O)C1OC1C=O)
BZeoO7_C3DI	9	10	10	0	O=C=CC(OO)C(O)C(O)C(=O)(OOC(C=O)C=O)
BZeoO7_MALb	10	12	11	0	O=C=CC(OO)C(O)C(O)C(=O)(OOC(C=O)C(O)C=O)
BZeoO7_C6a	12	12	8	0	O=C=CC(OO)C(O)C(O)C(=O)(OOc1ccccc1)
BZeoO7_NBZa	10	11	13	1	O=C=CC(OO)C(O)C(O)C(=O)(OOC1C(=O)OCC1ON(=O)=O)
BZeoO7_BZFU	10	12	11	0	O=C=CC(OO)C(O)C(O)C(=O)(OOC1C(O)COC1=O)
BZeoO7_C3a	9	10	11	0	O=C=CC(OO)C(O)C(O)C(=O)(OO(C(=O)C(C=O)O))
BZeoO7_CATE	12	12	9	0	O=C=CC(OO)C(O)C(O)C(=O)(OOc1ccccc1O)
BZeoO7_C2a	8	8	10	0	O=C=CC(OO)C(O)C(O)C(=O)(OOC(=O)C=O)
BZeoO7_C2b	8	10	9	0	O=C=CC(OO)C(O)C(O)C(=O)(OOCC=O)
BZeoO7_NPHb	12	11	10	1	O=C=CC(OO)C(O)C(O)C(=O)(OOc1ccccc1N(=O)=O)
BZeoO7_NNC	12	12	17	2	O=C=CC(OO)C(O)C(O)C(=O)(OOC1(O)C(=CC2(OOC1C2ON(=O)=O)N(=O)=O)O)
BZeoO7_NCAT	12	13	15	1	O=C=CC(OO)C(O)C(O)C(=O)(OOC1(O)C(=CC2(OOC1C2O)N(=O)=O)O)
BZeoO7_NBZb	12	11	13	1	O=C=CC(OO)C(O)C(O)C(=O)(OOC1C(=O)C=CC(=O)C1ON(=O)=O)
BZeoO7_PBZ	12	12	11	0	O=C=CC(OO)C(O)C(O)C(=O)(OOC1C(=O)C=CC(=O)C1O)
BZeoO7_MALc	10	10	12	0	O=C=CC(OO)C(O)C(O)C(=O)(OOC1C(=O)OC(=O)C1O)
BZeoO7_NDN	12	11	18	3	O=C=CC(OO)C(O)C(O)C(=O)(OOC1(O)C(=CC2(OOC1C2ON(=O)=O)N(=O)=O)N(=O)=O)
BZeoO7_DNP	12	12	16	2	O=C=CC(OO)C(O)C(O)C(=O)(OOC1(O)C(=CC2(OOC1C2O)N(=O)=O)N(=O)=O)
BZeoO7_C2c	8	10	10	0	O=C=CC(OO)C(O)C(O)C(=O)(OO(C(=O)CO))
BZeoO7_C5a	12	12	12	0	O=C=CC(OO)C(O)C(O)C(=O)(OO(C(=O)C=CC(=O)C(C=O)O))
BZeoO7_C4a	11	10	11	0	O=C=CC(OO)C(O)C(O)C(=O)(OOC(=O)C=CC(=O)C=O)
BZeoO9_BZeoO9	12	14	18	0	O=C(OO(C(C(O)C(=O)CC(OO)C(=O)OO)=O))C(O)C(=O)CC(OO)C(=O)OO
BZeoO9_C5eO5	11	14	14	0	O=C(OO(C(C(O)C(O)C=O)C=O))C(O)C(=O)CC(OO)C(=O)OO
BZeoO9_C5O6	11	14	15	0	O=C(OO(C(=O)C(O)C(O)C=COO))C(O)C(=O)CC(OO)C(=O)OO
BZeoO9_C5eO7	11	14	16	0	O=C(OO(C(=O)C(O)C(O)C(OO)C=O))C(O)C(=O)CC(OO)C(=O)OO
BZeoO9_C5O8	11	14	17	0	O=C(OO(C(C(O)C(O)C(O)C(=O)OO)=O))C(O)C(=O)CC(OO)C(=O)OO
BZeoO9_C5eO9	11	14	18	0	O=C(OO(C(C(OO)C(O)C(O)C(OO)=O)=O))C(O)C(=O)CC(OO)C(=O)OO
BZeoO9_BZBI	12	14	13	0	O=C(OOC1C=CC2OOC1C2O)C(O)C(=O)CC(OO)C(=O)OO
BZeoO9_BZMUa	12	12	13	0	O=C(OOC(=O)C1OC1C=CC=O)C(O)C(=O)CC(OO)C(=O)OO
BZeoO9_BZMUb	12	14	14	0	O=C(OO(C(C1OC1C=O)C(O)C=O))C(O)C(=O)CC(OO)C(=O)OO
BZeoO9_C5DI	11	12	12	0	O=C(OO(C(C=O)C=CC=O))C(O)C(=O)CC(OO)C(=O)OO
BZeoO9_NPHa	12	13	16	1	O=C(OOC1(O)C=CC2OOC1C2ON(=O)=O)C(O)C(=O)CC(OO)C(=O)OO
BZeoO9_PHEN	12	14	14	0	O=C(OOC1(O)C=CC2OOC1C2O)C(O)C(=O)CC(OO)C(=O)OO
BZeoO9_MALa	10	10	12	0	O=C(OOC(=O)C=CC=O)C(O)C(=O)CC(OO)C(=O)OO
BZeoO9_EPX	10	10	13	0	O=C(OOC(=O)C1OC1C=O)C(O)C(=O)CC(OO)C(=O)OO
BZeoO9_C3DI	9	10	12	0	O=C(OOC(C=O)C=O)C(O)C(=O)CC(OO)C(=O)OO
BZeoO9_MALb	10	12	13	0	O=C(OOC(C=O)C(O)C=O)C(O)C(=O)CC(OO)C(=O)OO
BZeoO9_C6a	12	12	10	0	O=C(OOc1ccccc1)C(O)C(=O)CC(OO)C(=O)OO
BZeoO9_NBZa	10	11	15	1	O=C(OOC1C(=O)OCC1ON(=O)=O)C(O)C(=O)CC(OO)C(=O)OO
BZeoO9_BZFU	10	12	13	0	O=C(OOC1C(O)COC1=O)C(O)C(=O)CC(OO)C(=O)OO
BZeoO9_C3a	9	10	13	0	O=C(OO(C(=O)C(C=O)O))C(O)C(=O)CC(OO)C(=O)OO
BZeoO9_CATE	12	12	11	0	O=C(OOc1ccccc1O)C(O)C(=O)CC(OO)C(=O)OO
BZeoO9_C2a	8	8	12	0	O=C(OOC(=O)C=O)C(O)C(=O)CC(OO)C(=O)OO
BZeoO9_C2b	8	10	11	0	O=C(OOCC=O)C(O)C(=O)CC(OO)C(=O)OO
BZeoO9_NPHb	12	11	12	1	O=C(OOc1ccccc1N(=O)=O)C(O)C(=O)CC(OO)C(=O)OO
BZeoO9_NNC	12	12	19	2	O=C(OOC1(O)C(=CC2(OOC1C2ON(=O)=O)N(=O)=O)O)C(O)C(=O)CC(OO)C(=O)OO
BZeoO9_NCAT	12	13	17	1	O=C(OOC1(O)C(=CC2(OOC1C2O)N(=O)=O)O)C(O)C(=O)CC(OO)C(=O)OO
BZeoO9_NBZb	12	11	15	1	O=C(OOC1C(=O)C=CC(=O)C1ON(=O)=O)C(O)C(=O)CC(OO)C(=O)OO
BZeoO9_PBZ	12	12	13	0	O=C(OOC1C(=O)C=CC(=O)C1O)C(O)C(=O)CC(OO)C(=O)OO
BZeoO9_MALc	10	10	14	0	O=C(OOC1C(=O)OC(=O)C1O)C(O)C(=O)CC(OO)C(=O)OO

BZeoO9_NDN	12	11	20	3	O=C(OOC1(O)C(=CC2(OOC1C2ON(=O)=O)N(=O)=O)N(=O)=O)C(O)C(=O)CC(OO)C(=O)OO
BZeoO9_DNP	12	12	18	2	O=C(OOC1(O)C(=CC2(OOC1C2O)N(=O)=O)N(=O)=O)C(O)C(=O)CC(OO)C(=O)OO
BZeoO9_C2c	8	10	12	0	O=C(OO(C(=O)CO))C(O)C(=O)CC(OO)C(=O)OO
BZeoO9_C5a	12	12	14	0	O=C(OO(C(=O)C=CC(=O)C(C=O)O))C(O)C(=O)CC(OO)C(=O)OO
BZeoO9_C4a	11	10	13	0	O=C(OOC(=O)C=CC(=O)C=O)C(O)C(=O)CC(OO)C(=O)OO
C5eO5_C5eO5	10	14	10	0	O=CC(OO(C(C(O)C(O)C=O)C=O))C(O)C(O)C=O
C5eO5_C5O6	10	14	11	0	O=CC(OO(C(=O)C(O)C(O)C=COO))C(O)C(O)C=O
C5eO5_C5eO7	10	14	12	0	O=CC(OO(C(=O)C(O)C(O)C(OO)C=O))C(O)C(O)C=O
C5eO5_C5O8	10	14	13	0	O=CC(OO(C(C(O)C(O)C(O)C(=O)OO)=O))C(O)C(O)C=O
C5eO5_C5eO9	10	14	14	0	O=CC(OO(C(C(OO)C(O)C(O)C(OO)=O)=O))C(O)C(O)C=O
C5eO5_BZBI	11	14	9	0	O=CC(OOC1C=CC2OOC1C2O)C(O)C(O)C=O
C5eO5_BZMUa	11	12	9	0	O=CC(OOC(=O)C1OC1C=CC=O)C(O)C(O)C=O
C5eO5_BZMUb	11	14	10	0	O=CC(OO(C(C1OC1C=O)C(O)C=O))C(O)C(O)C=O
C5eO5_C5DI	10	12	8	0	O=CC(OO(C(C=O)C=CC=O))C(O)C(O)C=O
C5eO5_NPHa	11	13	12	1	O=CC(OOC1(O)C=CC2OOC1C2ON(=O)=O)C(O)C(O)C=O
C5eO5_PHEN	11	14	10	0	O=CC(OOC1(O)C=CC2OOC1C2O)C(O)C(O)C=O
C5eO5_MALa	9	10	8	0	O=CC(OOC(=O)C=CC=O)C(O)C(O)C=O
C5eO5_EPX	9	10	9	0	O=CC(OOC(=O)C1OC1C=O)C(O)C(O)C=O
C5eO5_C3DI	8	10	8	0	O=CC(OOC(C=O)C=O)C(O)C(O)C=O
C5eO5_MALb	9	12	9	0	O=CC(OOC(C=O)C(O)C=O)C(O)C(O)C=O
C5eO5_C6a	11	12	6	0	O=CC(OOc1ccccc1)C(O)C(O)C=O
C5eO5_NBZa	9	11	11	1	O=CC(OOC1C(=O)OCC1ON(=O)=O)C(O)C(O)C=O
C5eO5_BZFU	9	12	9	0	O=CC(OOC1C(O)COC1=O)C(O)C(O)C=O
C5eO5_C3a	8	10	9	0	O=CC(OO(C(=O)C(C=O)O))C(O)C(O)C=O
C5eO5_CATE	11	12	7	0	O=CC(OOc1ccccc1O)C(O)C(O)C=O
C5eO5_C2a	7	8	8	0	O=CC(OOC(=O)C=O)C(O)C(O)C=O
C5eO5_C2b	7	10	7	0	O=CC(OOCC=O)C(O)C(O)C=O
C5eO5_NPHb	11	11	8	1	O=CC(OOc1ccccc1N(=O)=O)C(O)C(O)C=O
C5eO5_NNC	11	12	15	2	O=CC(OOC1(O)C(=CC2(OOC1C2ON(=O)=O)N(=O)=O)O)C(O)C(O)C=O
C5eO5_NCAT	11	13	13	1	O=CC(OOC1(O)C(=CC2(OOC1C2O)N(=O)=O)O)C(O)C(O)C=O
C5eO5_NBZb	11	11	11	1	O=CC(OOC1C(=O)C=CC(=O)C1ON(=O)=O)C(O)C(O)C=O
C5eO5_PBZ	11	12	9	0	O=CC(OOC1C(=O)C=CC(=O)C1O)C(O)C(O)C=O
C5eO5_MALc	9	10	10	0	O=CC(OOC1C(=O)OC(=O)C1O)C(O)C(O)C=O
C5eO5_NDN	11	11	16	3	O=CC(OOC1(O)C(=CC2(OOC1C2ON(=O)=O)N(=O)=O)N(=O)=O)C(O)C(O)C=O
C5eO5_DNP	11	12	14	2	O=CC(OOC1(O)C(=CC2(OOC1C2O)N(=O)=O)N(=O)=O)C(O)C(O)C=O
C5eO5_C2c	7	10	8	0	O=CC(OO(C(=O)CO))C(O)C(O)C=O
C5eO5_C5a	11	12	10	0	O=CC(OO(C(=O)C=CC(=O)C(C=O)O))C(O)C(O)C=O
C5eO5_C4a	10	10	9	0	O=CC(OOC(=O)C=CC(=O)C=O)C(O)C(O)C=O
C5O6_C5O6	10	14	12	0	OOC=CC(O)C(O)C(=O)(OO(C(=O)C(O)C(O)C=COO))
C5O6_C5eO7	10	14	13	0	OOC=CC(O)C(O)C(=O)(OO(C(=O)C(O)C(O)C(OO)C=O))
C5O6_C5O8	10	14	14	0	OOC=CC(O)C(O)C(=O)(OO(C(C(O)C(O)C(O)C(=O)OO)=O))
C5O6_C5eO9	10	14	15	0	OOC=CC(O)C(O)C(=O)(OO(C(C(OO)C(O)C(O)C(OO)=O)=O))
C5O6_BZBI	11	14	10	0	OOC=CC(O)C(O)C(=O)(OOC1C=CC2OOC1C2O)
C5O6_BZMUa	11	12	10	0	OOC=CC(O)C(O)C(=O)(OOC(=O)C1OC1C=CC=O)
C5O6_BZMUb	11	14	11	0	OOC=CC(O)C(O)C(=O)(OO(C(C1OC1C=O)C(O)C=O))
C5O6_C5DI	10	12	9	0	OOC=CC(O)C(O)C(=O)(OO(C(C=O)C=CC=O))
C5O6_NPHa	11	13	13	1	OOC=CC(O)C(O)C(=O)(OOC1(O)C=CC2OOC1C2ON(=O)=O)
C5O6_PHEN	11	14	11	0	OOC=CC(O)C(O)C(=O)(OOC1(O)C=CC2OOC1C2O)
C5O6_MALa	9	10	9	0	OOC=CC(O)C(O)C(=O)(OOC(=O)C=CC=O)
C5O6_EPX	9	10	10	0	OOC=CC(O)C(O)C(=O)(OOC(=O)C1OC1C=O)
C5O6_C3DI	8	10	9	0	OOC=CC(O)C(O)C(=O)(OOC(C=O)C=O)
C5O6_MALb	9	12	10	0	OOC=CC(O)C(O)C(=O)(OOC(C=O)C(O)C=O)
C5O6_C6a	11	12	7	0	OOC=CC(O)C(O)C(=O)(OOc1ccccc1)
C5O6_NBZa	9	11	12	1	OOC=CC(O)C(O)C(=O)(OOC1C(=O)OCC1ON(=O)=O)
C5O6_BZFU	9	12	10	0	OOC=CC(O)C(O)C(=O)(OOC1C(O)COC1=O)
C5O6_C3a	8	10	10	0	OOC=CC(O)C(O)C(=O)(OO(C(=O)C(C=O)O))
C5O6_CATE	11	12	8	0	OOC=CC(O)C(O)C(=O)(OOc1ccccc1O)
C5O6_C2a	7	8	9	0	OOC=CC(O)C(O)C(=O)(OOC(=O)C=O)
C5O6_C2b	7	10	8	0	OOC=CC(O)C(O)C(=O)(OOCC=O)
C5O6_NPHb	11	11	9	1	OOC=CC(O)C(O)C(=O)(OOc1ccccc1N(=O)=O)
C5O6_NNC	11	12	16	2	OOC=CC(O)C(O)C(=O)(OOC1(O)C(=CC2(OOC1C2ON(=O)=O)N(=O)=O)O)
C5O6_NCAT	11	13	14	1	OOC=CC(O)C(O)C(=O)(OOC1(O)C(=CC2(OOC1C2O)N(=O)=O)O)
C5O6_NBZb	11	11	12	1	OOC=CC(O)C(O)C(=O)(OOC1C(=O)C=CC(=O)C1ON(=O)=O)
C5O6_PBZ	11	12	10	0	OOC=CC(O)C(O)C(=O)(OOC1C(=O)C=CC(=O)C1O)
C5O6_MALc	9	10	11	0	OOC=CC(O)C(O)C(=O)(OOC1C(=O)OC(=O)C1O)
C5O6_NDN	11	11	17	3	OOC=CC(O)C(O)C(=O)(OOC1(O)C(=CC2(OOC1C2ON(=O)=O)N(=O)=O)N(=O)=O)
C5O6_DNP	11	12	15	2	OOC=CC(O)C(O)C(=O)(OOC1(O)C(=CC2(OOC1C2O)N(=O)=O)N(=O)=O)
C5O6_C2c	7	10	9	0	OOC=CC(O)C(O)C(=O)(OO(C(=O)CO))
C5O6_C5a	11	12	11	0	OOC=CC(O)C(O)C(=O)(OO(C(=O)C=CC(=O)C(C=O)O))
C5O6_C4a	10	10	10	0	OOC=CC(O)C(O)C(=O)(OOC(=O)C=CC(=O)C=O)
C5eO7_C5eO7	10	14	14	0	O=CC(OO)C(O)C(O)C(=O)(OO(C(=O)C(O)C(O)C(OO)C=O))
C5eO7_C5O8	10	14	15	0	O=CC(OO)C(O)C(O)C(=O)(OO(C(C(O)C(O)C(O)C(=O)OO)=O))
C5eO7_C5eO9	10	14	16	0	O=CC(OO)C(O)C(O)C(=O)(OO(C(C(OO)C(O)C(O)C(OO)=O)=O))
C5eO7_BZBI	11	14	11	0	O=CC(OO)C(O)C(O)C(=O)(OOC1C=CC2OOC1C2O)
C5eO7_BZMUa	11	12	11	0	O=CC(OO)C(O)C(O)C(=O)(OOC(=O)C1OC1C=CC=O)

C5eO7_BZMUb	11	14	12	0	O=CC(OO)C(O)C(O)C(=O)(OO(C(C1OC1C=O)C(O)C=O))
C5eO7_C5DI	10	12	10	0	O=CC(OO)C(O)C(O)C(=O)(OO(C(C=O)C=CC=O))
C5eO7_NPHa	11	13	14	1	O=CC(OO)C(O)C(O)C(=O)(OOC1(O)C=CC2OOC1C2ON(=O)=O)
C5eO7_PHEN	11	14	12	0	O=CC(OO)C(O)C(O)C(=O)(OOC1(O)C=CC2OOC1C2O)
C5eO7_MALa	9	10	10	0	O=CC(OO)C(O)C(O)C(=O)(OOC(=O)C=CC=O)
C5eO7_EPX	9	10	11	0	O=CC(OO)C(O)C(O)C(=O)(OOC(=O)C1OC1C=O)
C5eO7_C3DI	8	10	10	0	O=CC(OO)C(O)C(O)C(=O)(OOC(C=O)C=O)
C5eO7_MALb	9	12	11	0	O=CC(OO)C(O)C(O)C(=O)(OOC(C=O)C(O)C=O)
C5eO7_C6a	11	12	8	0	O=CC(OO)C(O)C(O)C(=O)(OOc1ccccc1)
C5eO7_NBZa	9	11	13	1	O=CC(OO)C(O)C(O)C(=O)(OOC1C(=O)OCC1ON(=O)=O)
C5eO7_BZFU	9	12	11	0	O=CC(OO)C(O)C(O)C(=O)(OOC1C(O)COC1=O)
C5eO7_C3a	8	10	11	0	O=CC(OO)C(O)C(O)C(=O)(OO(C(=O)C(C=O)O))
C5eO7_CATE	11	12	9	0	O=CC(OO)C(O)C(O)C(=O)(OOc1ccccc1O)
C5eO7_C2a	7	8	10	0	O=CC(OO)C(O)C(O)C(=O)(OOC(=O)C=O)
C5eO7_C2b	7	10	9	0	O=CC(OO)C(O)C(O)C(=O)(OOCC=O)
C5eO7_NPHb	11	11	10	1	O=CC(OO)C(O)C(O)C(=O)(OOc1ccccc1N(=O)=O)
C5eO7_NNC	11	12	17	2	O=CC(OO)C(O)C(O)C(=O)(OOC1(O)C(=CC2(OOC1C2ON(=O)=O)N(=O)=O)O)
C5eO7_NCAT	11	13	15	1	O=CC(OO)C(O)C(O)C(=O)(OOC1(O)C(=CC2(OOC1C2O)N(=O)=O)O)
C5eO7_NBZb	11	11	13	1	O=CC(OO)C(O)C(O)C(=O)(OOC1C(=O)C=CC(=O)C1ON(=O)=O)
C5eO7_PBZ	11	12	11	0	O=CC(OO)C(O)C(O)C(=O)(OOC1C(=O)C=CC(=O)C1O)
C5eO7_MALc	9	10	12	0	O=CC(OO)C(O)C(O)C(=O)(OOC1C(=O)OC(=O)C1O)
C5eO7_NDN	11	11	18	3	O=CC(OO)C(O)C(O)C(=O)(OOC1(O)C(=CC2(OOC1C2ON(=O)=O)N(=O)=O)N(=O)=O)
C5eO7_DNP	11	12	16	2	O=CC(OO)C(O)C(O)C(=O)(OOC1(O)C(=CC2(OOC1C2O)N(=O)=O)N(=O)=O)
C5eO7_C2c	7	10	10	0	O=CC(OO)C(O)C(O)C(=O)(OO(C(=O)CO))
C5eO7_C5a	11	12	12	0	O=CC(OO)C(O)C(O)C(=O)(OO(C(=O)C=CC(=O)C(C=O)O))
C5eO7_C4a	10	10	11	0	O=CC(OO)C(O)C(O)C(=O)(OOC(=O)C=CC(=O)C=O)
C5O8_C5O8	10	14	16	0	O=C(OO(C(C(O)C(O)C(O)C(=O)OO)=O))C(O)C(O)C(O)C(=O)OO
C5O8_C5eO9	10	14	17	0	O=C(OO(C(C(OO)C(O)C(O)C(OO)=O)=O))C(O)C(O)C(O)C(=O)OO
C5O8_BZBI	11	14	12	0	O=C(OOC1C=CC2OOC1C2O)C(O)C(O)C(O)C(=O)OO
C5O8_BZMUa	11	12	12	0	O=C(OOC(=O)C1OC1C=CC=O)C(O)C(O)C(O)C(=O)OO
C5O8_BZMUb	11	14	13	0	O=C(OO(C(C1OC1C=O)C(O)C=O))C(O)C(O)C(O)C(=O)OO
C5O8_C5DI	10	12	11	0	O=C(OO(C(C=O)C=CC=O))C(O)C(O)C(O)C(=O)OO
C5O8_NPHa	11	13	15	1	O=C(OOC1(O)C=CC2OOC1C2ON(=O)=O)C(O)C(O)C(O)C(=O)OO
C5O8_PHEN	11	14	13	0	O=C(OOC1(O)C=CC2OOC1C2O)C(O)C(O)C(O)C(=O)OO
C5O8_MALa	9	10	11	0	O=C(OOC(=O)C=CC=O)C(O)C(O)C(O)C(=O)OO
C5O8_EPX	9	10	12	0	O=C(OOC(=O)C1OC1C=O)C(O)C(O)C(O)C(=O)OO
C5O8_C3DI	8	10	11	0	O=C(OOC(C=O)C=O)C(O)C(O)C(O)C(=O)OO
C5O8_MALb	9	12	12	0	O=C(OOC(C=O)C(O)C=O)C(O)C(O)C(O)C(=O)OO
C5O8_C6a	11	12	9	0	O=C(OOc1ccccc1)C(O)C(O)C(O)C(=O)OO
C5O8_NBZa	9	11	14	1	O=C(OOC1C(=O)OCC1ON(=O)=O)C(O)C(O)C(O)C(=O)OO
C5O8_BZFU	9	12	12	0	O=C(OOC1C(O)COC1=O)C(O)C(O)C(O)C(=O)OO
C5O8_C3a	8	10	12	0	O=C(OO(C(=O)C(C=O)O))C(O)C(O)C(O)C(=O)OO
C5O8_CATE	11	12	10	0	O=C(OOc1ccccc1O)C(O)C(O)C(O)C(=O)OO
C5O8_C2a	7	8	11	0	O=C(OOC(=O)C=O)C(O)C(O)C(O)C(=O)OO
C5O8_C2b	7	10	10	0	O=C(OOCC=O)C(O)C(O)C(O)C(=O)OO
C5O8_NPHb	11	11	11	1	O=C(OOc1ccccc1N(=O)=O)C(O)C(O)C(O)C(=O)OO
C5O8_NNC	11	12	18	2	O=C(OOC1(O)C(=CC2(OOC1C2ON(=O)=O)N(=O)=O)O)C(O)C(O)C(O)C(=O)OO
C5O8_NCAT	11	13	16	1	O=C(OOC1(O)C(=CC2(OOC1C2O)N(=O)=O)O)C(O)C(O)C(O)C(=O)OO
C5O8_NBZb	11	11	14	1	O=C(OOC1C(=O)C=CC(=O)C1ON(=O)=O)C(O)C(O)C(O)C(=O)OO
C5O8_PBZ	11	12	12	0	O=C(OOC1C(=O)C=CC(=O)C1O)C(O)C(O)C(O)C(=O)OO
C5O8_MALc	9	10	13	0	O=C(OOC1C(=O)OC(=O)C1O)C(O)C(O)C(O)C(=O)OO
C5O8_NDN	11	11	19	3	O=C(OOC1(O)C(=CC2(OOC1C2ON(=O)=O)N(=O)=O)N(=O)=O)C(O)C(O)C(O)C(=O)OO
C5O8_DNP	11	12	17	2	O=C(OOC1(O)C(=CC2(OOC1C2O)N(=O)=O)N(=O)=O)C(O)C(O)C(O)C(=O)OO
C5O8_C2c	7	10	11	0	O=C(OO(C(=O)CO))C(O)C(O)C(O)C(=O)OO
C5O8_C5a	11	12	13	0	O=C(OO(C(=O)C=CC(=O)C(C=O)O))C(O)C(O)C(O)C(=O)OO
C5O8_C4a	10	10	12	0	O=C(OOC(=O)C=CC(=O)C=O)C(O)C(O)C(O)C(=O)OO
C5eO9_C5eO9	10	14	18	0	O=C(OO(C(C(OO)C(O)C(O)C(OO)=O)=O))C(OO)C(O)C(O)C(OO)=O
C5eO9_BZBI	11	14	13	0	O=C(OOC1C=CC2OOC1C2O)C(OO)C(O)C(O)C(OO)=O
C5eO9_BZMUa	11	12	13	0	O=C(OOC(=O)C1OC1C=CC=O)C(OO)C(O)C(O)C(OO)=O
C5eO9_BZMUb	11	14	14	0	O=C(OO(C(C1OC1C=O)C(O)C=O))C(OO)C(O)C(O)C(OO)=O
C5eO9_C5DI	10	12	12	0	O=C(OO(C(C=O)C=CC=O))C(OO)C(O)C(O)C(OO)=O
C5eO9_NPHa	11	13	16	1	O=C(OOC1(O)C=CC2OOC1C2ON(=O)=O)C(OO)C(O)C(O)C(OO)=O
C5eO9_PHEN	11	14	14	0	O=C(OOC1(O)C=CC2OOC1C2O)C(OO)C(O)C(O)C(OO)=O
C5eO9_MALa	9	10	12	0	O=C(OOC(=O)C=CC=O)C(OO)C(O)C(O)C(OO)=O
C5eO9_EPX	9	10	13	0	O=C(OOC(=O)C1OC1C=O)C(OO)C(O)C(O)C(OO)=O
C5eO9_C3DI	8	10	12	0	O=C(OOC(C=O)C=O)C(OO)C(O)C(O)C(OO)=O
C5eO9_MALb	9	12	13	0	O=C(OOC(C=O)C(O)C=O)C(OO)C(O)C(O)C(OO)=O
C5eO9_C6a	11	12	10	0	O=C(OOc1ccccc1)C(OO)C(O)C(O)C(OO)=O
C5eO9_NBZa	9	11	15	1	O=C(OOC1C(=O)OCC1ON(=O)=O)C(OO)C(O)C(O)C(OO)=O
C5eO9_BZFU	9	12	13	0	O=C(OOC1C(O)COC1=O)C(OO)C(O)C(O)C(OO)=O
C5eO9_C3a	8	10	13	0	O=C(OO(C(=O)C(C=O)O))C(OO)C(O)C(O)C(OO)=O
C5eO9_CATE	11	12	11	0	O=C(OOc1ccccc1O)C(OO)C(O)C(O)C(OO)=O
C5eO9_C2a	7	8	12	0	O=C(OOC(=O)C=O)C(OO)C(O)C(O)C(OO)=O
C5eO9_C2b	7	10	11	0	O=C(OOCC=O)C(OO)C(O)C(O)C(OO)=O
C5eO9_NPHb	11	11	12	1	O=C(OOc1ccccc1N(=O)=O)C(OO)C(O)C(O)C(OO)=O

C5eO9_NNC	11	12	19	2	O=C(OOC1(O)C(=CC2(OOC1C2ON(=O)=O)N(=O)=O)O)C(OO)C(O)C(O)C(OO)=O
C5eO9_NCAT	11	13	17	1	O=C(OOC1(O)C(=CC2(OOC1C2O)N(=O)=O)O)C(OO)C(O)C(O)C(OO)=O
C5eO9_NBZb	11	11	15	1	O=C(OOC1C(=O)C=CC(=O)C1ON(=O)=O)C(OO)C(O)C(O)C(OO)=O
C5eO9_PBZ	11	12	13	0	O=C(OOC1C(=O)C=CC(=O)C1O)C(OO)C(O)C(O)C(OO)=O
C5eO9_MALc	9	10	14	0	O=C(OOC1C(=O)OC(=O)C1O)C(OO)C(O)C(O)C(OO)=O
C5eO9_NDN	11	11	20	3	O=C(OOC1(O)C(=CC2(OOC1C2ON(=O)=O)N(=O)=O)N(=O)=O)C(OO)C(O)C(O)C(OO)=O
C5eO9_DNP	11	12	18	2	O=C(OOC1(O)C(=CC2(OOC1C2O)N(=O)=O)N(=O)=O)C(OO)C(O)C(O)C(OO)=O
C5eO9_C2c	7	10	12	0	O=C(OO(C(=O)CO))C(OO)C(O)C(O)C(OO)=O
C5eO9_C5a	11	12	14	0	O=C(OO(C(=O)C=CC(=O)C(C=O)O))C(OO)C(O)C(O)C(OO)=O
C5eO9_C4a	10	10	13	0	O=C(OOC(=O)C=CC(=O)C=O)C(OO)C(O)C(O)C(OO)=O
BZBI_BZBI	12	14	8	0	O(C1C=CC2OOC1C2O)OC1C=CC2OOC1C2O
BZBI_BZMUa	12	12	8	0	O(C1C=CC2OOC1C2O)OC(=O)C1OC1C=CC=O
BZBI_BZMUb	12	14	9	0	O=CC(O)C(OOC1C=CC2OOC1C2O)C1OC1C=O
BZBI_C5DI	11	12	7	0	O=CC=CC(OOC1C=CC2OOC1C2O)C=O
BZBI_NPHa	12	13	11	1	O(C1C=CC2OOC1C2O)OC1(O)C=CC2OOC1C2ON(=O)=O
BZBI_PHEN	12	14	9	0	O(C1C=CC2OOC1C2O)OC1(O)C=CC2OOC1C2O
BZBI_MALa	10	10	7	0	O(C1C=CC2OOC1C2O)OC(=O)C=CC=O
BZBI_EPX	10	10	8	0	O(C1C=CC2OOC1C2O)OC(=O)C1OC1C=O
BZBI_C3DI	9	10	7	0	O(C1C=CC2OOC1C2O)OC(C=O)C=O
BZBI_MALb	10	12	8	0	O(C1C=CC2OOC1C2O)OC(C=O)C(O)C=O
BZBI_C6a	12	12	5	0	O(C1C=CC2OOC1C2O)Oc1ccccc1
BZBI_NBZa	10	11	10	1	O(C1C=CC2OOC1C2O)OC1C(=O)OCC1ON(=O)=O
BZBI_BZFU	10	12	8	0	O(C1C=CC2OOC1C2O)OC1C(O)COC1=O
BZBI_C3a	9	10	8	0	OC(C=O)C(=O)(OOC1C=CC2OOC1C2O)
BZBI_CATE	12	12	6	0	O(C1C=CC2OOC1C2O)Oc1ccccc1O
BZBI_C2a	8	8	7	0	O(C1C=CC2OOC1C2O)OC(=O)C=O
BZBI_C2b	8	10	6	0	O(C1C=CC2OOC1C2O)OCC=O
BZBI_NPHb	12	11	7	1	O(C1C=CC2OOC1C2O)Oc1ccccc1N(=O)=O
BZBI_NNC	12	12	14	2	O(C1C=CC2OOC1C2O)OC1(O)C(=CC2(OOC1C2ON(=O)=O)N(=O)=O)O
BZBI_NCAT	12	13	12	1	O(C1C=CC2OOC1C2O)OC1(O)C(=CC2(OOC1C2O)N(=O)=O)O
BZBI_NBZb	12	11	10	1	O(C1C=CC2OOC1C2O)OC1C(=O)C=CC(=O)C1ON(=O)=O
BZBI_PBZ	12	12	8	0	O(C1C=CC2OOC1C2O)OC1C(=O)C=CC(=O)C1O
BZBI_MALc	10	10	9	0	O(C1C=CC2OOC1C2O)OC1C(=O)OC(=O)C1O
BZBI_NDN	12	11	15	3	O(C1C=CC2OOC1C2O)OC1(O)C(=CC2(OOC1C2ON(=O)=O)N(=O)=O)N(=O)=O
BZBI_DNP	12	12	13	2	O(C1C=CC2OOC1C2O)OC1(O)C(=CC2(OOC1C2O)N(=O)=O)N(=O)=O
BZBI_C2c	8	10	7	0	OCC(=O)(OOC1C=CC2OOC1C2O)
BZBI_C5a	12	12	9	0	OC(C=O)C(=O)C=CC(=O)(OOC1C=CC2OOC1C2O)
BZBI_C4a	11	10	8	0	O(C1C=CC2OOC1C2O)OC(=O)C=CC(=O)C=O
BZMUa_BZMUa	12	10	8	0	O(C(=O)C1OC1C=CC=O)OC(=O)C1OC1C=CC=O
BZMUa_BZMUb	12	12	9	0	O=CC(O)C(OOC(=O)C1OC1C=CC=O)C1OC1C=O
BZMUa_C5DI	11	10	7	0	O=CC=CC(OOC(=O)C1OC1C=CC=O)C=O
BZMUa_NPHa	12	11	11	1	O(C(=O)C1OC1C=CC=O)OC1(O)C=CC2OOC1C2ON(=O)=O
BZMUa_PHEN	12	12	9	0	O(C(=O)C1OC1C=CC=O)OC1(O)C=CC2OOC1C2O
BZMUa_MALa	10	8	7	0	O(C(=O)C1OC1C=CC=O)OC(=O)C=CC=O
BZMUa_EPX	10	8	8	0	O(C(=O)C1OC1C=CC=O)OC(=O)C1OC1C=O
BZMUa_C3DI	9	8	7	0	O(C(=O)C1OC1C=CC=O)OC(C=O)C=O
BZMUa_MALb	10	10	8	0	O(C(=O)C1OC1C=CC=O)OC(C=O)C(O)C=O
BZMUa_C6a	12	10	5	0	O(C(=O)C1OC1C=CC=O)Oc1ccccc1
BZMUa_NBZa	10	9	10	1	O(C(=O)C1OC1C=CC=O)OC1C(=O)OCC1ON(=O)=O
BZMUa_BZFU	10	10	8	0	O(C(=O)C1OC1C=CC=O)OC1C(O)COC1=O
BZMUa_C3a	9	8	8	0	OC(C=O)C(=O)(OOC(=O)C1OC1C=CC=O)
BZMUa_CATE	12	10	6	0	O(C(=O)C1OC1C=CC=O)Oc1ccccc1O
BZMUa_C2a	8	6	7	0	O(C(=O)C1OC1C=CC=O)OC(=O)C=O
BZMUa_C2b	8	8	6	0	O(C(=O)C1OC1C=CC=O)OCC=O
BZMUa_NPHb	12	9	7	1	O(C(=O)C1OC1C=CC=O)Oc1ccccc1N(=O)=O
BZMUa_NNC	12	10	14	2	O(C(=O)C1OC1C=CC=O)OC1(O)C(=CC2(OOC1C2ON(=O)=O)N(=O)=O)O
BZMUa_NCAT	12	11	12	1	O(C(=O)C1OC1C=CC=O)OC1(O)C(=CC2(OOC1C2O)N(=O)=O)O
BZMUa_NBZb	12	9	10	1	O(C(=O)C1OC1C=CC=O)OC1C(=O)C=CC(=O)C1ON(=O)=O
BZMUa_PBZ	12	10	8	0	O(C(=O)C1OC1C=CC=O)OC1C(=O)C=CC(=O)C1O
BZMUa_MALc	10	8	9	0	O(C(=O)C1OC1C=CC=O)OC1C(=O)OC(=O)C1O
BZMUa_NDN	12	9	15	3	O(C(=O)C1OC1C=CC=O)OC1(O)C(=CC2(OOC1C2ON(=O)=O)N(=O)=O)N(=O)=O
BZMUa_DNP	12	10	13	2	O(C(=O)C1OC1C=CC=O)OC1(O)C(=CC2(OOC1C2O)N(=O)=O)N(=O)=O
BZMUa_C2c	8	8	7	0	OCC(=O)(OOC(=O)C1OC1C=CC=O)
BZMUa_C5a	12	10	9	0	OC(C=O)C(=O)C=CC(=O)(OOC(=O)C1OC1C=CC=O)
BZMUa_C4a	11	8	8	0	O(C(=O)C1OC1C=CC=O)OC(=O)C=CC(=O)C=O
BZMUb_BZMUb	12	14	10	0	O=CC(O)C(OO(C(C1OC1C=O)C(O)C=O))C1OC1C=O
BZMUb_C5DI	11	12	8	0	O=CC(O)C(OO(C(C=O)C=CC=O))C1OC1C=O
BZMUb_NPHa	12	13	12	1	O=CC(O)C(OOC1(O)C=CC2OOC1C2ON(=O)=O)C1OC1C=O
BZMUb_PHEN	12	14	10	0	O=CC(O)C(OOC1(O)C=CC2OOC1C2O)C1OC1C=O
BZMUb_MALa	10	10	8	0	O=CC(O)C(OOC(=O)C=CC=O)C1OC1C=O
BZMUb_EPX	10	10	9	0	O=CC(O)C(OOC(=O)C1OC1C=O)C1OC1C=O
BZMUb_C3DI	9	10	8	0	O=CC(O)C(OOC(C=O)C=O)C1OC1C=O
BZMUb_MALb	10	12	9	0	O=CC(O)C(OOC(C=O)C(O)C=O)C1OC1C=O
BZMUb_C6a	12	12	6	0	O=CC(O)C(OOc1ccccc1)C1OC1C=O
BZMUb_NBZa	10	11	11	1	O=CC(O)C(OOC1C(=O)OCC1ON(=O)=O)C1OC1C=O

BZMUb_BZFU	10	12	9	0	O=CC(O)C(OOC1C(O)COC1=O)C1OC1C=O
BZMUb_C3a	9	10	9	0	O=CC(O)C(OO(C(=O)C(C=O)O))C1OC1C=O
BZMUb_CATE	12	12	7	0	O=CC(O)C(OOc1ccccc1O)C1OC1C=O
BZMUb_C2a	8	8	8	0	O=CC(O)C(OOC(=O)C=O)C1OC1C=O
BZMUb_C2b	8	10	7	0	O=CC(O)C(OOCC=O)C1OC1C=O
BZMUb_NPHb	12	11	8	1	O=CC(O)C(OOc1ccccc1N(=O)=O)C1OC1C=O
BZMUb_NNC	12	12	15	2	O=CC(O)C(OOC1(O)C(=CC2(OOC1C2ON(=O)=O)N(=O)=O)O)C1OC1C=O
BZMUb_NCAT	12	13	13	1	O=CC(O)C(OOC1(O)C(=CC2(OOC1C2O)N(=O)=O)O)C1OC1C=O
BZMUb_NBZb	12	11	11	1	O=CC(O)C(OOC1C(=O)C=CC(=O)C1ON(=O)=O)C1OC1C=O
BZMUb_PBZ	12	12	9	0	O=CC(O)C(OOC1C(=O)C=CC(=O)C1O)C1OC1C=O
BZMUb_MALc	10	10	10	0	O=CC(O)C(OOC1C(=O)OC(=O)C1O)C1OC1C=O
BZMUb_NDN	12	11	16	3	O=CC(O)C(OOC1(O)C(=CC2(OOC1C2ON(=O)=O)N(=O)=O)N(=O)=O)C1OC1C=O
BZMUb_DNP	12	12	14	2	O=CC(O)C(OOC1(O)C(=CC2(OOC1C2O)N(=O)=O)N(=O)=O)C1OC1C=O
BZMUb_C2c	8	10	8	0	O=CC(O)C(OO(C(=O)CO))C1OC1C=O
BZMUb_C5a	12	12	10	0	O=CC(O)C(OO(C(=O)C=CC(=O)C(C=O)O))C1OC1C=O
BZMUb_C4a	11	10	9	0	O=CC(O)C(OOC(=O)C=CC(=O)C=O)C1OC1C=O
C5DI_C5DI	10	10	6	0	O=CC=CC(OO(C(C=O)C=CC=O))C=O
C5DI_NPHa	11	11	10	1	O=CC=CC(OOC1(O)C=CC2OOC1C2ON(=O)=O)C=O
C5DI_PHEN	11	12	8	0	O=CC=CC(OOC1(O)C=CC2OOC1C2O)C=O
C5DI_MALa	9	8	6	0	O=CC=CC(OOC(=O)C=CC=O)C=O
C5DI_EPX	9	8	7	0	O=CC=CC(OOC(=O)C1OC1C=O)C=O
C5DI_C3DI	8	8	6	0	O=CC=CC(OOC(C=O)C=O)C=O
C5DI_MALb	9	10	7	0	O=CC=CC(OOC(C=O)C(O)C=O)C=O
C5DI_C6a	11	10	4	0	O=CC=CC(OOc1ccccc1)C=O
C5DI_NBZa	9	9	9	1	O=CC=CC(OOC1C(=O)OCC1ON(=O)=O)C=O
C5DI_BZFU	9	10	7	0	O=CC=CC(OOC1C(O)COC1=O)C=O
C5DI_C3a	8	8	7	0	O=CC=CC(OO(C(=O)C(C=O)O))C=O
C5DI_CATE	11	10	5	0	O=CC=CC(OOc1ccccc1O)C=O
C5DI_C2a	7	6	6	0	O=CC=CC(OOC(=O)C=O)C=O
C5DI_C2b	7	8	5	0	O=CC=CC(OOCC=O)C=O
C5DI_NPHb	11	9	6	1	O=CC=CC(OOc1ccccc1N(=O)=O)C=O
C5DI_NNC	11	10	13	2	O=CC=CC(OOC1(O)C(=CC2(OOC1C2ON(=O)=O)N(=O)=O)O)C=O
C5DI_NCAT	11	11	11	1	O=CC=CC(OOC1(O)C(=CC2(OOC1C2O)N(=O)=O)O)C=O
C5DI_NBZb	11	9	9	1	O=CC=CC(OOC1C(=O)C=CC(=O)C1ON(=O)=O)C=O
C5DI_PBZ	11	10	7	0	O=CC=CC(OOC1C(=O)C=CC(=O)C1O)C=O
C5DI_MALc	9	8	8	0	O=CC=CC(OOC1C(=O)OC(=O)C1O)C=O
C5DI_NDN	11	9	14	3	O=CC=CC(OOC1(O)C(=CC2(OOC1C2ON(=O)=O)N(=O)=O)N(=O)=O)C=O
C5DI_DNP	11	10	12	2	O=CC=CC(OOC1(O)C(=CC2(OOC1C2O)N(=O)=O)N(=O)=O)C=O
C5DI_C2c	7	8	6	0	O=CC=CC(OO(C(=O)CO))C=O
C5DI_C5a	11	10	8	0	O=CC=CC(OO(C(=O)C=CC(=O)C(C=O)O))C=O
C5DI_C4a	10	8	7	0	O=CC=CC(OOC(=O)C=CC(=O)C=O)C=O
NPHa_NPHa	12	12	14	2	O(C1(O)C=CC2OOC1C2ON(=O)=O)OC1(O)C=CC2OOC1C2ON(=O)=O
NPHa_PHEN	12	13	12	1	O(C1(O)C=CC2OOC1C2ON(=O)=O)OC1(O)C=CC2OOC1C2O
NPHa_MALa	10	9	10	1	O(C1(O)C=CC2OOC1C2ON(=O)=O)OC(=O)C=CC=O
NPHa_EPX	10	9	11	1	O(C1(O)C=CC2OOC1C2ON(=O)=O)OC(=O)C1OC1C=O
NPHa_C3DI	9	9	10	1	O(C1(O)C=CC2OOC1C2ON(=O)=O)OC(C=O)C=O
NPHa_MALb	10	11	11	1	O(C1(O)C=CC2OOC1C2ON(=O)=O)OC(C=O)C(O)C=O
NPHa_C6a	12	11	8	1	O(C1(O)C=CC2OOC1C2ON(=O)=O)Oc1ccccc1
NPHa_NBZa	10	10	13	2	O(C1(O)C=CC2OOC1C2ON(=O)=O)OC1C(=O)OCC1ON(=O)=O
NPHa_BZFU	10	11	11	1	O(C1(O)C=CC2OOC1C2ON(=O)=O)OC1C(O)COC1=O
NPHa_C3a	9	9	11	1	OC(C=O)C(=O)(OOC1(O)C=CC2OOC1C2ON(=O)=O)
NPHa_CATE	12	11	9	1	O(C1(O)C=CC2OOC1C2ON(=O)=O)Oc1ccccc1O
NPHa_C2a	8	7	10	1	O(C1(O)C=CC2OOC1C2ON(=O)=O)OC(=O)C=O
NPHa_C2b	8	9	9	1	O(C1(O)C=CC2OOC1C2ON(=O)=O)OCC=O
NPHa_NPHb	12	10	10	2	O(C1(O)C=CC2OOC1C2ON(=O)=O)Oc1ccccc1N(=O)=O
NPHa_NNC	12	11	17	3	O(C1(O)C=CC2OOC1C2ON(=O)=O)OC1(O)C(=CC2(OOC1C2ON(=O)=O)N(=O)=O)O
NPHa_NCAT	12	12	15	2	O(C1(O)C=CC2OOC1C2ON(=O)=O)OC1(O)C(=CC2(OOC1C2O)N(=O)=O)O
NPHa_NBZb	12	10	13	2	O(C1(O)C=CC2OOC1C2ON(=O)=O)OC1C(=O)C=CC(=O)C1ON(=O)=O
NPHa_PBZ	12	11	11	1	O(C1(O)C=CC2OOC1C2ON(=O)=O)OC1C(=O)C=CC(=O)C1O
NPHa_MALc	10	9	12	1	O(C1(O)C=CC2OOC1C2ON(=O)=O)OC1C(=O)OC(=O)C1O
NPHa_NDN	12	10	18	4	O(C1(O)C=CC2OOC1C2ON(=O)=O)OC1(O)C(=CC2(OOC1C2ON(=O)=O)N(=O)=O)N(=O)=O
NPHa_DNP	12	11	16	3	O(C1(O)C=CC2OOC1C2ON(=O)=O)OC1(O)C(=CC2(OOC1C2O)N(=O)=O)N(=O)=O
NPHa_C2c	8	9	10	1	OCC(=O)(OOC1(O)C=CC2OOC1C2ON(=O)=O)
NPHa_C5a	12	11	12	1	OC(C=O)C(=O)C=CC(=O)(OOC1(O)C=CC2OOC1C2ON(=O)=O)
NPHa_C4a	11	9	11	1	O(C1(O)C=CC2OOC1C2ON(=O)=O)OC(=O)C=CC(=O)C=O
PHEN_PHEN	12	14	10	0	O(C1(O)C=CC2OOC1C2O)OC1(O)C=CC2OOC1C2O
PHEN_MALa	10	10	8	0	O(C1(O)C=CC2OOC1C2O)OC(=O)C=CC=O
PHEN_EPX	10	10	9	0	O(C1(O)C=CC2OOC1C2O)OC(=O)C1OC1C=O
PHEN_C3DI	9	10	8	0	O(C1(O)C=CC2OOC1C2O)OC(C=O)C=O
PHEN_MALb	10	12	9	0	O(C1(O)C=CC2OOC1C2O)OC(C=O)C(O)C=O
PHEN_C6a	12	12	6	0	O(C1(O)C=CC2OOC1C2O)Oc1ccccc1
PHEN_NBZa	10	11	11	1	O(C1(O)C=CC2OOC1C2O)OC1C(=O)OCC1ON(=O)=O
PHEN_BZFU	10	12	9	0	O(C1(O)C=CC2OOC1C2O)OC1C(O)COC1=O
PHEN_C3a	9	10	9	0	OC(C=O)C(=O)(OOC1(O)C=CC2OOC1C2O)
PHEN_CATE	12	12	7	0	O(C1(O)C=CC2OOC1C2O)Oc1ccccc1O

PHEN_C2a	8	8	8	0	O(C1(O)C=CC2OOC1C2O)OC(=O)C=O
PHEN_C2b	8	10	7	0	O(C1(O)C=CC2OOC1C2O)OCC=O
PHEN_NPHb	12	11	8	1	O(C1(O)C=CC2OOC1C2O)Oc1ccccc1N(=O)=O
PHEN_NNC	12	12	15	2	O(C1(O)C=CC2OOC1C2O)OC1(O)C(=CC2(OOC1C2ON(=O)=O)N(=O)=O)O
PHEN_NCAT	12	13	13	1	O(C1(O)C=CC2OOC1C2O)OC1(O)C(=CC2(OOC1C2O)N(=O)=O)O
PHEN_NBZb	12	11	11	1	O(C1(O)C=CC2OOC1C2O)OC1C(=O)C=CC(=O)C1ON(=O)=O
PHEN_PBZ	12	12	9	0	O(C1(O)C=CC2OOC1C2O)OC1C(=O)C=CC(=O)C1O
PHEN_MALc	10	10	10	0	O(C1(O)C=CC2OOC1C2O)OC1C(=O)OC(=O)C1O
PHEN_NDN	12	11	16	3	O(C1(O)C=CC2OOC1C2O)OC1(O)C(=CC2(OOC1C2ON(=O)=O)N(=O)=O)N(=O)=O
PHEN_DNP	12	12	14	2	O(C1(O)C=CC2OOC1C2O)OC1(O)C(=CC2(OOC1C2O)N(=O)=O)N(=O)=O
PHEN_C2c	8	10	8	0	OCC(=O)(OOC1(O)C=CC2OOC1C2O)
PHEN_C5a	12	12	10	0	OC(C=O)C(=O)C=CC(=O)(OOC1(O)C=CC2OOC1C2O)
PHEN_C4a	11	10	9	0	O(C1(O)C=CC2OOC1C2O)OC(=O)C=CC(=O)C=O
MALa_MALa	8	6	6	0	O(C(=O)C=CC=O)OC(=O)C=CC=O
MALa_EPX	8	6	7	0	O(C(=O)C=CC=O)OC(=O)C1OC1C=O
MALa_C3DI	7	6	6	0	O(C(=O)C=CC=O)OC(C=O)C=O
MALa_MALb	8	8	7	0	O(C(=O)C=CC=O)OC(C=O)C(O)C=O
MALa_C6a	10	8	4	0	O(C(=O)C=CC=O)Oc1ccccc1
MALa_NBZa	8	7	9	1	O(C(=O)C=CC=O)OC1C(=O)OCC1ON(=O)=O
MALa_BZFU	8	8	7	0	O(C(=O)C=CC=O)OC1C(O)COC1=O
MALa_C3a	7	6	7	0	OC(C=O)C(=O)(OOC(=O)C=CC=O)
MALa_CATE	10	8	5	0	O(C(=O)C=CC=O)Oc1ccccc1O
MALa_C2a	6	4	6	0	O(C(=O)C=CC=O)OC(=O)C=O
MALa_C2b	6	6	5	0	O(C(=O)C=CC=O)OCC=O
MALa_NPHb	10	7	6	1	O(C(=O)C=CC=O)Oc1ccccc1N(=O)=O
MALa_NNC	10	8	13	2	O(C(=O)C=CC=O)OC1(O)C(=CC2(OOC1C2ON(=O)=O)N(=O)=O)O
MALa_NCAT	10	9	11	1	O(C(=O)C=CC=O)OC1(O)C(=CC2(OOC1C2O)N(=O)=O)O
MALa_NBZb	10	7	9	1	O(C(=O)C=CC=O)OC1C(=O)C=CC(=O)C1ON(=O)=O
MALa_PBZ	10	8	7	0	O(C(=O)C=CC=O)OC1C(=O)C=CC(=O)C1O
MALa_MALc	8	6	8	0	O(C(=O)C=CC=O)OC1C(=O)OC(=O)C1O
MALa_NDN	10	7	14	3	O(C(=O)C=CC=O)OC1(O)C(=CC2(OOC1C2ON(=O)=O)N(=O)=O)N(=O)=O
MALa_DNP	10	8	12	2	O(C(=O)C=CC=O)OC1(O)C(=CC2(OOC1C2O)N(=O)=O)N(=O)=O
MALa_C2c	6	6	6	0	OCC(=O)(OOC(=O)C=CC=O)
MALa_C5a	10	8	8	0	OC(C=O)C(=O)C=CC(=O)(OOC(=O)C=CC=O)
MALa_C4a	9	6	7	0	O(C(=O)C=CC=O)OC(=O)C=CC(=O)C=O
EPX_EPX	8	6	8	0	O(C(=O)C1OC1C=O)OC(=O)C1OC1C=O
EPX_C3DI	7	6	7	0	O(C(=O)C1OC1C=O)OC(C=O)C=O
EPX_MALb	8	8	8	0	O(C(=O)C1OC1C=O)OC(C=O)C(O)C=O
EPX_C6a	10	8	5	0	O(C(=O)C1OC1C=O)Oc1ccccc1
EPX_NBZa	8	7	10	1	O(C(=O)C1OC1C=O)OC1C(=O)OCC1ON(=O)=O
EPX_BZFU	8	8	8	0	O(C(=O)C1OC1C=O)OC1C(O)COC1=O
EPX_C3a	7	6	8	0	OC(C=O)C(=O)(OOC(=O)C1OC1C=O)
EPX_CATE	10	8	6	0	O(C(=O)C1OC1C=O)Oc1ccccc1O
EPX_C2a	6	4	7	0	O(C(=O)C1OC1C=O)OC(=O)C=O
EPX_C2b	6	6	6	0	O(C(=O)C1OC1C=O)OCC=O
EPX_NPHb	10	7	7	1	O(C(=O)C1OC1C=O)Oc1ccccc1N(=O)=O
EPX_NNC	10	8	14	2	O(C(=O)C1OC1C=O)OC1(O)C(=CC2(OOC1C2ON(=O)=O)N(=O)=O)O
EPX_NCAT	10	9	12	1	O(C(=O)C1OC1C=O)OC1(O)C(=CC2(OOC1C2O)N(=O)=O)O
EPX_NBZb	10	7	10	1	O(C(=O)C1OC1C=O)OC1C(=O)C=CC(=O)C1ON(=O)=O
EPX_PBZ	10	8	8	0	O(C(=O)C1OC1C=O)OC1C(=O)C=CC(=O)C1O
EPX_MALc	8	6	9	0	O(C(=O)C1OC1C=O)OC1C(=O)OC(=O)C1O
EPX_NDN	10	7	15	3	O(C(=O)C1OC1C=O)OC1(O)C(=CC2(OOC1C2ON(=O)=O)N(=O)=O)N(=O)=O
EPX_DNP	10	8	13	2	O(C(=O)C1OC1C=O)OC1(O)C(=CC2(OOC1C2O)N(=O)=O)N(=O)=O
EPX_C2c	6	6	7	0	OCC(=O)(OOC(=O)C1OC1C=O)
EPX_C5a	10	8	9	0	OC(C=O)C(=O)C=CC(=O)(OOC(=O)C1OC1C=O)
EPX_C4a	9	6	8	0	O(C(=O)C1OC1C=O)OC(=O)C=CC(=O)C=O
C3DI_C3DI	6	6	6	0	O(C(C=O)C=O)OC(C=O)C=O
C3DI_MALb	7	8	7	0	O(C(C=O)C=O)OC(C=O)C(O)C=O
C3DI_C6a	9	8	4	0	O(C(C=O)C=O)Oc1ccccc1
C3DI_NBZa	7	7	9	1	O(C(C=O)C=O)OC1C(=O)OCC1ON(=O)=O
C3DI_BZFU	7	8	7	0	O(C(C=O)C=O)OC1C(O)COC1=O
C3DI_C3a	6	6	7	0	OC(C=O)C(=O)(OOC(C=O)C=O)
C3DI_CATE	9	8	5	0	O(C(C=O)C=O)Oc1ccccc1O
C3DI_C2a	5	4	6	0	O(C(C=O)C=O)OC(=O)C=O
C3DI_C2b	5	6	5	0	O(C(C=O)C=O)OCC=O
C3DI_NPHb	9	7	6	1	O(C(C=O)C=O)Oc1ccccc1N(=O)=O
C3DI_NNC	9	8	13	2	O(C(C=O)C=O)OC1(O)C(=CC2(OOC1C2ON(=O)=O)N(=O)=O)O
C3DI_NCAT	9	9	11	1	O(C(C=O)C=O)OC1(O)C(=CC2(OOC1C2O)N(=O)=O)O
C3DI_NBZb	9	7	9	1	O(C(C=O)C=O)OC1C(=O)C=CC(=O)C1ON(=O)=O
C3DI_PBZ	9	8	7	0	O(C(C=O)C=O)OC1C(=O)C=CC(=O)C1O
C3DI_MALc	7	6	8	0	O(C(C=O)C2=O)OC1C(=O)OC(=O)C1O
C3DI_NDN	9	7	14	3	O(C(C=O)C=O)OC1(O)C(=CC2(OOC1C2ON(=O)=O)N(=O)=O)N(=O)=O
C3DI_DNP	9	8	12	2	O(C(C=O)C=O)OC1(O)C(=CC2(OOC1C2O)N(=O)=O)N(=O)=O
C3DI_C2c	5	6	6	0	OCC(=O)(OOC(C=O)C=O)
C3DI_C5a	9	8	8	0	OC(C=O)C(=O)C=CC(=O)(OOC(C=O)C=O)

C3DI_C4a	8	6	7	0	O(C(C=O)C=O)OC(=O)C=CC(=O)C=O
MALb_MALb	8	10	8	0	O(C(C=O)C(O)C=O)OC(C=O)C(O)C=O
MALb_C6a	10	10	5	0	O(C(C=O)C(O)C=O)Oc1ccccc1
MALb_NBZa	8	9	10	1	O(C(C=O)C(O)C=O)OC1C(=O)OCC1ON(=O)=O
MALb_BZFU	8	10	8	0	O(C(C=O)C(O)C=O)OC1C(O)COC1=O
MALb_C3a	7	8	8	0	OC(C=O)C(=O)(OOC(C=O)C(O)C=O)
MALb_CATE	10	10	6	0	O(C(C=O)C(O)C=O)Oc1ccccc1O
MALb_C2a	6	6	7	0	O(C(C=O)C(O)C=O)OC(=O)C=O
MALb_C2b	6	8	6	0	O(C(C=O)C(O)C=O)OCC=O
MALb_NPHb	10	9	7	1	O(C(C=O)C(O)C=O)Oc1ccccc1N(=O)=O
MALb_NNC	10	10	14	2	O(C(C=O)C(O)C=O)OC1(O)C(=CC2(OOC1C2ON(=O)=O)N(=O)=O)O
MALb_NCAT	10	11	12	1	O(C(C=O)C(O)C=O)OC1(O)C(=CC2(OOC1C2O)N(=O)=O)O
MALb_NBZb	10	9	10	1	O(C(C=O)C(O)C=O)OC1C(=O)C=CC(=O)C1ON(=O)=O
MALb_PBZ	10	10	8	0	O(C(C=O)C(O)C=O)OC1C(=O)C=CC(=O)C1O
MALb_MALc	8	8	9	0	O(C(C=O)C(O)C=O)OC1C(=O)OC(=O)C1O
MALb_NDN	10	9	15	3	O(C(C=O)C(O)C=O)OC1(O)C(=CC2(OOC1C2ON(=O)=O)N(=O)=O)N(=O)=O
MALb_DNP	10	10	13	2	O(C(C=O)C(O)C=O)OC1(O)C(=CC2(OOC1C2O)N(=O)=O)N(=O)=O
MALb_C2c	6	8	7	0	OCC(=O)(OOC(C=O)C(O)C=O)
MALb_C5a	10	10	9	0	OC(C=O)C(=O)C=CC(=O)(OOC(C=O)C(O)C=O)
MALb_C4a	9	8	8	0	O(C(C=O)C(O)C=O)OC(=O)C=CC(=O)C=O
C6a_C6a	12	10	2	0	O(c1ccccc1)Oc1ccccc1
C6a_NBZa	10	9	7	1	O(c1ccccc1)OC1C(=O)OCC1ON(=O)=O
C6a_BZFU	10	10	5	0	O(c1ccccc1)OC1C(O)COC1=O
C6a_C3a	9	8	5	0	OC(C=O)C(=O)(OOc1ccccc1)
C6a_CATE	12	10	3	0	O(c1ccccc1)Oc1ccccc1O
C6a_C2a	8	6	4	0	O(c1ccccc1)OC(=O)C=O
C6a_C2b	8	8	3	0	O(c1ccccc1)OCC=O
C6a_NPHb	12	9	4	1	O(c1ccccc1)Oc1ccccc1N(=O)=O
C6a_NNC	12	10	11	2	O(c1ccccc1)OC1(O)C(=CC2(OOC1C2ON(=O)=O)N(=O)=O)O
C6a_NCAT	12	11	9	1	O(c1ccccc1)OC1(O)C(=CC2(OOC1C2O)N(=O)=O)O
C6a_NBZb	12	9	7	1	O(c1ccccc1)OC1C(=O)C=CC(=O)C1ON(=O)=O
C6a_PBZ	12	10	5	0	O(c1ccccc1)OC1C(=O)C=CC(=O)C1O
C6a_MALc	10	8	6	0	O(c1ccccc1)OC1C(=O)OC(=O)C1O
C6a_NDN	12	9	12	3	O(c1ccccc1)OC1(O)C(=CC2(OOC1C2ON(=O)=O)N(=O)=O)N(=O)=O
C6a_DNP	12	10	10	2	O(c1ccccc1)OC1(O)C(=CC2(OOC1C2O)N(=O)=O)N(=O)=O
C6a_C2c	8	8	4	0	OCC(=O)(OOc1ccccc1)
C6a_C5a	12	10	6	0	OC(C=O)C(=O)C=CC(=O)(OOc1ccccc1)
C6a_C4a	11	8	5	0	O(c1ccccc1)OC(=O)C=CC(=O)C=O
NBZa_NBZa	8	8	12	2	O(C1C(=O)OCC1ON(=O)=O)OC1C(=O)OCC1ON(=O)=O
NBZa_BZFU	8	9	10	1	O(C1C(=O)OCC1ON(=O)=O)OC1C(O)COC1=O
NBZa_C3a	7	7	10	1	OC(C=O)C(=O)(OOC1C(=O)OCC1ON(=O)=O)
NBZa_CATE	10	9	8	1	O(C1C(=O)OCC1ON(=O)=O)Oc1ccccc1O
NBZa_C2a	6	5	9	1	O(C1C(=O)OCC1ON(=O)=O)OC(=O)C=O
NBZa_C2b	6	7	8	1	O(C1C(=O)OCC1ON(=O)=O)OCC=O
NBZa_NPHb	10	8	9	2	O(C1C(=O)OCC1ON(=O)=O)Oc1ccccc1N(=O)=O
NBZa_NNC	10	9	16	3	O(C1C(=O)OCC1ON(=O)=O)OC1(O)C(=CC2(OOC1C2ON(=O)=O)N(=O)=O)O
NBZa_NCAT	10	10	14	2	O(C1C(=O)OCC1ON(=O)=O)OC1(O)C(=CC2(OOC1C2O)N(=O)=O)O
NBZa_NBZb	10	8	12	2	O(C1C(=O)OCC1ON(=O)=O)OC1C(=O)C=CC(=O)C1ON(=O)=O
NBZa_PBZ	10	9	10	1	O(C1C(=O)OCC1ON(=O)=O)OC1C(=O)C=CC(=O)C1O
NBZa_MALc	8	7	11	1	O(C1C(=O)OCC1ON(=O)=O)OC1C(=O)OC(=O)C1O
NBZa_NDN	10	8	17	4	O(C1C(=O)OCC1ON(=O)=O)OC1(O)C(=CC2(OOC1C2ON(=O)=O)N(=O)=O)N(=O)=O
NBZa_DNP	10	9	15	3	O(C1C(=O)OCC1ON(=O)=O)OC1(O)C(=CC2(OOC1C2O)N(=O)=O)N(=O)=O
NBZa_C2c	6	7	9	1	OCC(=O)(OOC1C(=O)OCC1ON(=O)=O)
NBZa_C5a	10	9	11	1	OC(C=O)C(=O)C=CC(=O)(OOC1C(=O)OCC1ON(=O)=O)
NBZa_C4a	9	7	10	1	O(C1C(=O)OCC1ON(=O)=O)OC(=O)C=CC(=O)C=O
BZFU_BZFU	8	10	8	0	O(C1C(O)COC1=O)OC1C(O)COC1=O
BZFU_C3a	7	8	8	0	OC(C=O)C(=O)(OOC1C(O)COC1=O)
BZFU_CATE	10	10	6	0	O(C1C(O)COC1=O)Oc1ccccc1O
BZFU_C2a	6	6	7	0	O(C1C(O)COC1=O)OC(=O)C=O
BZFU_C2b	6	8	6	0	O(C1C(O)COC1=O)OCC=O
BZFU_NPHb	10	9	7	1	O(C1C(O)COC1=O)Oc1ccccc1N(=O)=O
BZFU_NNC	10	10	14	2	O(C1C(O)COC1=O)OC1(O)C(=CC2(OOC1C2ON(=O)=O)N(=O)=O)O
BZFU_NCAT	10	11	12	1	O(C1C(O)COC1=O)OC1(O)C(=CC2(OOC1C2O)N(=O)=O)O
BZFU_NBZb	10	9	10	1	O(C1C(O)COC1=O)OC1C(=O)C=CC(=O)C1ON(=O)=O
BZFU_PBZ	10	10	8	0	O(C1C(O)COC1=O)OC1C(=O)C=CC(=O)C1O
BZFU_MALc	8	8	9	0	O(C1C(O)COC1=O)OC1C(=O)OC(=O)C1O
BZFU_NDN	10	9	15	3	O(C1C(O)COC1=O)OC1(O)C(=CC2(OOC1C2ON(=O)=O)N(=O)=O)N(=O)=O
BZFU_DNP	10	10	13	2	O(C1C(O)COC1=O)OC1(O)C(=CC2(OOC1C2O)N(=O)=O)N(=O)=O
BZFU_C2c	6	8	7	0	OCC(=O)(OOC1C(O)COC1=O)
BZFU_C5a	10	10	9	0	OC(C=O)C(=O)C=CC(=O)(OOC1C(O)COC1=O)
BZFU_C4a	9	8	8	0	O(C1C(O)COC1=O)OC(=O)C=CC(=O)C=O
C3a_C3a	6	6	8	0	OC(C=O)C(=O)(OO(C(=O)C(C=O)O))
C3a_CATE	9	8	6	0	OC(C=O)C(=O)(OOc1ccccc1O)
C3a_C2a	5	4	7	0	OC(C=O)C(=O)(OOC(=O)C=O)
C3a_C2b	5	6	6	0	OC(C=O)C(=O)(OOCC=O)

C3a_NPHb	9	7	7	1	OC(C=O)C(=O)(OOc1ccccc1N(=O)=O)
C3a_NNC	9	8	14	2	OC(C=O)C(=O)(OOC1(O)C(=CC2(OOC1C2ON(=O)=O)N(=O)=O)O)
C3a_NCAT	9	9	12	1	OC(C=O)C(=O)(OOC1(O)C(=CC2(OOC1C2O)N(=O)=O)O)
C3a_NBZb	9	7	10	1	OC(C=O)C(=O)(OOC1C(=O)C=CC(=O)C1ON(=O)=O)
C3a_PBZ	9	8	8	0	OC(C=O)C(=O)(OOC1C(=O)C=CC(=O)C1O)
C3a_MALc	7	6	9	0	OC(C=O)C(=O)(OOC1C(=O)OC(=O)C1O)
C3a_NDN	9	7	15	3	OC(C=O)C(=O)(OOC1(O)C(=CC2(OOC1C2ON(=O)=O)N(=O)=O)N(=O)=O)
C3a_DNP	9	8	13	2	OC(C=O)C(=O)(OOC1(O)C(=CC2(OOC1C2O)N(=O)=O)N(=O)=O)
C3a_C2c	5	6	7	0	OC(C=O)C(=O)(OO(C(=O)CO))
C3a_C5a	9	8	9	0	OC(C=O)C(=O)(OO(C(=O)C=CC(=O)C(C=O)O))
C3a_C4a	8	6	8	0	OC(C=O)C(=O)(OOC(=O)C=CC(=O)C=O)
CATE_CATE	12	10	4	0	O(c1ccccc1O)Oc1ccccc1O
CATE_C2a	8	6	5	0	O(c1ccccc1O)OC(=O)C=O
CATE_C2b	8	8	4	0	O(c1ccccc1O)OCC=O
CATE_NPHb	12	9	5	1	O(c1ccccc1O)Oc1ccccc1N(=O)=O
CATE_NNC	12	10	12	2	O(c1ccccc1O)OC1(O)C(=CC2(OOC1C2ON(=O)=O)N(=O)=O)O
CATE_NCAT	12	11	10	1	O(c1ccccc1O)OC1(O)C(=CC2(OOC1C2O)N(=O)=O)O
CATE_NBZb	12	9	8	1	O(c1ccccc1O)OC1C(=O)C=CC(=O)C1ON(=O)=O
CATE_PBZ	12	10	6	0	O(c1ccccc1O)OC1C(=O)C=CC(=O)C1O
CATE_MALc	10	8	7	0	O(c1ccccc1O)OC1C(=O)OC(=O)C1O
CATE_NDN	12	9	13	3	O(c1ccccc1O)OC1(O)C(=CC2(OOC1C2ON(=O)=O)N(=O)=O)N(=O)=O
CATE_DNP	12	10	11	2	O(c1ccccc1O)OC1(O)C(=CC2(OOC1C2O)N(=O)=O)N(=O)=O
CATE_C2c	8	8	5	0	OCC(=O)(OOc1ccccc1O)
CATE_C5a	12	10	7	0	OC(C=O)C(=O)C=CC(=O)(OOc1ccccc1O)
CATE_C4a	11	8	6	0	O(c1ccccc1O)OC(=O)C=CC(=O)C=O
C2a_C2a	4	2	6	0	O(C(=O)C=O)OC(=O)C=O
C2a_C2b	4	4	5	0	O(C(=O)C=O)OCC=O
C2a_NPHb	8	5	6	1	O(C(=O)C=O)Oc1ccccc1N(=O)=O
C2a_NNC	8	6	13	2	O(C(=O)C=O)OC1(O)C(=CC2(OOC1C2ON(=O)=O)N(=O)=O)O
C2a_NCAT	8	7	11	1	O(C(=O)C=O)OC1(O)C(=CC2(OOC1C2O)N(=O)=O)O
C2a_NBZb	8	5	9	1	O(C(=O)C=O)OC1C(=O)C=CC(=O)C1ON(=O)=O
C2a_PBZ	8	6	7	0	O(C(=O)C=O)OC1C(=O)C=CC(=O)C1O
C2a_MALc	6	4	8	0	O(C(=O)C=O)OC1C(=O)OC(=O)C1O
C2a_NDN	8	5	14	3	O(C(=O)C=O)OC1(O)C(=CC2(OOC1C2ON(=O)=O)N(=O)=O)N(=O)=O
C2a_DNP	8	6	12	2	O(C(=O)C=O)OC1(O)C(=CC2(OOC1C2O)N(=O)=O)N(=O)=O
C2a_C2c	4	4	6	0	OCC(=O)(OOC(=O)C=O)
C2a_C5a	8	6	8	0	OC(C=O)C(=O)C=CC(=O)(OOC(=O)C=O)
C2a_C4a	7	4	7	0	O(C(=O)C=O)OC(=O)C=CC(=O)C=O
C2b_C2b	4	6	4	0	O(CC=O)OCC=O
C2b_NPHb	8	7	5	1	O(CC=O)Oc1ccccc1N(=O)=O
C2b_NNC	8	8	12	2	O(CC=O)OC1(O)C(=CC2(OOC1C2ON(=O)=O)N(=O)=O)O
C2b_NCAT	8	9	10	1	O(CC=O)OC1(O)C(=CC2(OOC1C2O)N(=O)=O)O
C2b_NBZb	8	7	8	1	O(CC=O)OC1C(=O)C=CC(=O)C1ON(=O)=O
C2b_PBZ	8	8	6	0	O(CC=O)OC1C(=O)C=CC(=O)C1O
C2b_MALc	6	6	7	0	O(CC=O)OC1C(=O)OC(=O)C1O
C2b_NDN	8	7	13	3	O(CC=O)OC1(O)C(=CC2(OOC1C2ON(=O)=O)N(=O)=O)N(=O)=O
C2b_DNP	8	8	11	2	O(CC=O)OC1(O)C(=CC2(OOC1C2O)N(=O)=O)N(=O)=O
C2b_C2c	4	6	5	0	OCC(=O)(OOCC=O)
C2b_C5a	8	8	7	0	OC(C=O)C(=O)C=CC(=O)(OOCC=O)
C2b_C4a	7	6	6	0	O(CC=O)OC(=O)C=CC(=O)C=O
NPHb_NPHb	12	8	6	2	O(c1ccccc1N(=O)=O)Oc1ccccc1N(=O)=O
NPHb_NNC	12	9	13	3	O(c1ccccc1N(=O)=O)OC1(O)C(=CC2(OOC1C2ON(=O)=O)N(=O)=O)O
NPHb_NCAT	12	10	11	2	O(c1ccccc1N(=O)=O)OC1(O)C(=CC2(OOC1C2O)N(=O)=O)O
NPHb_NBZb	12	8	9	2	O(c1ccccc1N(=O)=O)OC1C(=O)C=CC(=O)C1ON(=O)=O
NPHb_PBZ	12	9	7	1	O(c1ccccc1N(=O)=O)OC1C(=O)C=CC(=O)C1O
NPHb_MALc	10	7	8	1	O(c1ccccc1N(=O)=O)OC1C(=O)OC(=O)C1O
NPHb_NDN	12	8	14	4	O(c1ccccc1N(=O)=O)OC1(O)C(=CC2(OOC1C2ON(=O)=O)N(=O)=O)N(=O)=O
NPHb_DNP	12	9	12	3	O(c1ccccc1N(=O)=O)OC1(O)C(=CC2(OOC1C2O)N(=O)=O)N(=O)=O
NPHb_C2c	8	7	6	1	OCC(=O)(OOc1ccccc1N(=O)=O)
NPHb_C5a	12	9	8	1	OC(C=O)C(=O)C=CC(=O)(OOc1ccccc1N(=O)=O)
NPHb_C4a	11	7	7	1	O(c1ccccc1N(=O)=O)OC(=O)C=CC(=O)C=O
NNC_NNC	12	10	20	4	O(C1(O)C(=CC2(OOC1C2ON(=O)=O)N(=O)=O)O)OC1(O)C(=CC2(OOC1C2ON(=O)=O)N(=O)=O)O
NNC_NCAT	12	11	18	3	O(C1(O)C(=CC2(OOC1C2ON(=O)=O)N(=O)=O)O)OC1(O)C(=CC2(OOC1C2O)N(=O)=O)O
NNC_NBZb	12	9	16	3	O(C1(O)C(=CC2(OOC1C2ON(=O)=O)N(=O)=O)O)OC1C(=O)C=CC(=O)C1ON(=O)=O
NNC_PBZ	12	10	14	2	O(C1(O)C(=CC2(OOC1C2ON(=O)=O)N(=O)=O)O)OC1C(=O)C=CC(=O)C1O
NNC_MALc	10	8	15	2	O(C1(O)C(=CC2(OOC1C2ON(=O)=O)N(=O)=O)O)OC1C(=O)OC(=O)C1O
NNC_NDN	12	9	21	5	O(C1(O)C(=CC2(OOC1C2ON(=O)=O)N(=O)=O)O)OC1(O)C(=CC2(OOC1C2ON(=O)=O)N(=O)=O)N(=O)=O
NNC_DNP	12	10	19	4	O(C1(O)C(=CC2(OOC1C2ON(=O)=O)N(=O)=O)O)OC1(O)C(=CC2(OOC1C2O)N(=O)=O)N(=O)=O
NNC_C2c	8	8	13	2	OCC(=O)(OOC1(O)C(=CC2(OOC1C2ON(=O)=O)N(=O)=O)O)
NNC_C5a	12	10	15	2	OC(C=O)C(=O)C=CC(=O)(OOC1(O)C(=CC2(OOC1C2ON(=O)=O)N(=O)=O)O)
NNC_C4a	11	8	14	2	O(C1(O)C(=CC2(OOC1C2ON(=O)=O)N(=O)=O)O)OC(=O)C=CC(=O)C=O
NCAT_NCAT	12	12	16	2	O(C1(O)C(=CC2(OOC1C2O)N(=O)=O)O)OC1(O)C(=CC2(OOC1C2O)N(=O)=O)O
NCAT_NBZb	12	10	14	2	O(C1(O)C(=CC2(OOC1C2O)N(=O)=O)O)OC1C(=O)C=CC(=O)C1ON(=O)=O
NCAT_PBZ	12	11	12	1	O(C1(O)C(=CC2(OOC1C2O)N(=O)=O)O)OC1C(=O)C=CC(=O)C1O
NCAT_MALc	10	9	13	1	O(C1(O)C(=CC2(OOC1C2O)N(=O)=O)O)OC1C(=O)OC(=O)C1O

NCAT_NDN	12	10	19	4	O(C1(O)C(=CC2(OOC1C2O)N(=O)=O)O)OC1(O)C(=CC2(OOC1C2ON(=O)=O)N(=O)=O)N(=O)=O
NCAT_DNP	12	11	17	3	O(C1(O)C(=CC2(OOC1C2O)N(=O)=O)O)OC1(O)C(=CC2(OOC1C2O)N(=O)=O)N(=O)=O
NCAT_C2c	8	9	11	1	OCC(=O)(OOC1(O)C(=CC2(OOC1C2O)N(=O)=O)O)
NCAT_C5a	12	11	13	1	OC(C=O)C(=O)C=CC(=O)(OOC1(O)C(=CC2(OOC1C2O)N(=O)=O)O)
NCAT_C4a	11	9	12	1	O(C1(O)C(=CC2(OOC1C2O)N(=O)=O)O)OC(=O)C=CC(=O)C=O
NBZb_NBZb	12	8	12	2	O(C1C(=O)C=CC(=O)C1ON(=O)=O)OC1C(=O)C=CC(=O)C1ON(=O)=O
NBZb_PBZ	12	9	10	1	O(C1C(=O)C=CC(=O)C1ON(=O)=O)OC1C(=O)C=CC(=O)C1O
NBZb_MALc	10	7	11	1	O(C1C(=O)C=CC(=O)C1ON(=O)=O)OC1C(=O)OC(=O)C1O
NBZb_NDN	12	8	17	4	O(C1C(=O)C=CC(=O)C1ON(=O)=O)OC1(O)C(=CC2(OOC1C2ON(=O)=O)N(=O)=O)N(=O)=O
NBZb_DNP	12	9	15	3	O(C1C(=O)C=CC(=O)C1ON(=O)=O)OC1(O)C(=CC2(OOC1C2O)N(=O)=O)N(=O)=O
NBZb_C2c	8	7	9	1	OCC(=O)(OOC1C(=O)C=CC(=O)C1ON(=O)=O)
NBZb_C5a	12	9	11	1	OC(C=O)C(=O)C=CC(=O)(OOC1C(=O)C=CC(=O)C1ON(=O)=O)
NBZb_C4a	11	7	10	1	O(C1C(=O)C=CC(=O)C1ON(=O)=O)OC(=O)C=CC(=O)C=O
PBZ_PBZ	12	10	8	0	O(C1C(=O)C=CC(=O)C1O)OC1C(=O)C=CC(=O)C1O
PBZ_MALc	10	8	9	0	O(C1C(=O)C=CC(=O)C1O)OC1C(=O)OC(=O)C1O
PBZ_NDN	12	9	15	3	O(C1C(=O)C=CC(=O)C1O)OC1(O)C(=CC2(OOC1C2ON(=O)=O)N(=O)=O)N(=O)=O
PBZ_DNP	12	10	13	2	O(C1C(=O)C=CC(=O)C1O)OC1(O)C(=CC2(OOC1C2O)N(=O)=O)N(=O)=O
PBZ_C2c	8	8	7	0	OCC(=O)(OOC1C(=O)C=CC(=O)C1O)
PBZ_C5a	12	10	9	0	OC(C=O)C(=O)C=CC(=O)(OOC1C(=O)C=CC(=O)C1O)
PBZ_C4a	11	8	8	0	O(C1C(=O)C=CC(=O)C1O)OC(=O)C=CC(=O)C=O
MALc_MALc	8	6	10	0	O(C1C(=O)OC(=O)C1O)OC1C(=O)OC(=O)C1O
MALc_NDN	10	7	16	3	O(C1C(=O)OC(=O)C1O)OC1(O)C(=CC2(OOC1C2ON(=O)=O)N(=O)=O)N(=O)=O
MALc_DNP	10	8	14	2	O(C1C(=O)OC(=O)C1O)OC1(O)C(=CC2(OOC1C2O)N(=O)=O)N(=O)=O
MALc_C2c	6	6	8	0	OCC(=O)(OOC1C(=O)OC(=O)C1O)
MALc_C5a	10	8	10	0	OC(C=O)C(=O)C=CC(=O)(OOC1C(=O)OC(=O)C1O)
MALc_C4a	9	6	9	0	O(C1C(=O)OC(=O)C1O)OC(=O)C=CC(=O)C=O
NDN_NDN	12	8	22	6	O(C1(O)C(=CC2(OOC1C2ON(=O)=O)N(=O)=O)N(=O)=O)OC1(O)C(=CC2(OOC1C2ON(=O)=O)N(=O)=O)N(=O)=O
NDN_DNP	12	9	20	5	O(C1(O)C(=CC2(OOC1C2ON(=O)=O)N(=O)=O)N(=O)=O)OC1(O)C(=CC2(OOC1C2O)N(=O)=O)N(=O)=O
NDN_C2c	8	7	14	3	OCC(=O)(OOC1(O)C(=CC2(OOC1C2ON(=O)=O)N(=O)=O)N(=O)=O)
NDN_C5a	12	9	16	3	OC(C=O)C(=O)C=CC(=O)(OOC1(O)C(=CC2(OOC1C2ON(=O)=O)N(=O)=O)N(=O)=O)
NDN_C4a	11	7	15	3	O(C1(O)C(=CC2(OOC1C2ON(=O)=O)N(=O)=O)N(=O)=O)OC(=O)C=CC(=O)C=O
DNP_DNP	12	10	18	4	O(C1(O)C(=CC2(OOC1C2O)N(=O)=O)N(=O)=O)OC1(O)C(=CC2(OOC1C2O)N(=O)=O)N(=O)=O
DNP_C2c	8	8	12	2	OCC(=O)(OOC1(O)C(=CC2(OOC1C2O)N(=O)=O)N(=O)=O)
DNP_C5a	12	10	14	2	OC(C=O)C(=O)C=CC(=O)(OOC1(O)C(=CC2(OOC1C2O)N(=O)=O)N(=O)=O)
DNP_C4a	11	8	13	2	O(C1(O)C(=CC2(OOC1C2O)N(=O)=O)N(=O)=O)OC(=O)C=CC(=O)C=O
C2c_C2c	4	6	6	0	OCC(=O)(OO(C(=O)CO))
C2c_C5a	8	8	8	0	OCC(=O)(OO(C(=O)C=CC(=O)C(C=O)O))
C2c_C4a	7	6	7	0	OCC(=O)(OOC(=O)C=CC(=O)C=O)
C5a_C5a	12	10	10	0	OC(C=O)C(=O)C=CC(=O)(OO(C(=O)C=CC(=O)C(C=O)O))
C5a_C4a	11	8	9	0	OC(C=O)C(=O)C=CC(=O)(OOC(=O)C=CC(=O)C=O)
C4a_C4a	10	6	8	0	O(C(=O)C=CC(=O)C=O)OC(=O)C=CC(=O)C=O
BZo_O5_OOH	6	8	7	0	O=CC(O)C(O)C1OC1C(OO)=O
BZo_O7_OOH	6	8	9	0	O=C(OO)C(O)C(O)C1OC1C(OO)=O
BZo_O9_OOH	6	8	11	0	O=C(OO)C(O)C(O)C(=O)C(OO)C(=O)OO
BZeo_O4_OOH	6	8	6	0	OOC1C2OC2C3OOC1C3(O)
BZeo_O6_OOH	6	8	8	0	O=C=CC(OO)C(O)C(O)C(=O)OO
BZeo_O8_OOH	6	8	10	0	O=C(OO)C(O)C(=O)CC(OO)C(=O)OO
C5e_O4_OOH	5	8	6	0	O=CC(OO)C(O)C(O)C=O
C5_O5_OOH	5	8	7	0	OOC=CC(O)C(O)C(=O)OO
C5e_O6_OOH	5	8	8	0	O=CC(OO)C(O)C(O)C(OO)=O
C5_O7_OOH	5	8	9	0	O=C(OO)C(O)C(O)C(O)C(=O)OO
C5e_O8_OOH	5	8	10	0	O=C(OO)C(OO)C(O)C(O)C(OO)=O
BZo_O5_NO3	6	7	8	1	O=CC(O)C(O)C1OC1C(ON(=O)=O)=O
BZo_O7_NO3	6	7	10	1	O=C(ON(=O)=O)C(O)C(O)C1OC1C(OO)=O
BZo_O9_NO3	6	7	12	1	O=C(OO)C(O)C(O)C(=O)C(ON(=O)=O)C(=O)OO
BZeo_O4_NO3	6	7	7	1	O=N(=O)OC1C2OC2C3OOC1C3(O)
BZeo_O6_NO3	6	7	9	1	O=C=CC(OO)C(O)C(O)C(=O)ON(=O)=O
BZeo_O8_NO3	6	7	11	1	O=C(ON(=O)=O)C(O)C(=O)CC(OO)C(=O)OO
C5e_O4_NO3	5	7	7	1	O=CC(ON(=O)=O)C(O)C(O)C=O
C5_O5_NO3	5	7	8	1	OOC=CC(O)C(O)C(=O)ON(=O)=O
C5e_O6_NO3	5	7	9	1	O=CC(OO)C(O)C(O)C(ON(=O)=O)=O
C5_O7_NO3	5	7	10	1	O=C(ON(=O)=O)C(O)C(O)C(O)C(=O)OO
C5e_O8_NO3	5	7	11	1	O=C(ON(=O)=O)C(OO)C(O)C(O)C(OO)=O
ROCS_O5_O	6	6	6	0	O=CC(=O)C(O)C1OC1C(O)=O
ROCS_O7_O	6	6	8	0	O=C(O)C(=O)C(O)C1OC1C(OO)=O
ROCS_O9_O	6	6	10	0	O=C(OO)C(O)C(O)C(=O)C(=O)C(=O)OO
ROCS_O4_O	6	6	5	0	O=C1C2OC2C3OOC1C3(O)
ROCS_O6_O	6	6	7	0	O=C=CC(OO)C(=O)C(O)C(=O)O
ROCS_O8_O	6	6	9	0	O=C(O)C(=O)C(=O)CC(OO)C(=O)OO

Table ST7: basic properties of the autoAPRAM species for benzene, including species name, atomic composition and the molecular structure in SMILES format.

References SI tables:

1 D. R. Cocker, R. C. Flagan and J. H. Seinfeld, State-of-the-Art Chamber Facility for Studying Atmospheric Aerosol Chemistry, *Environ. Sci. Technol.*, 2001, **35**, 2594–2601.
